# Abstracts from the Bone and Muscle Interactions: The Mechanical and Beyond Meeting August 2019

**DOI:** 10.1002/jbm4.10257

**Published:** 2019-12-20

**Authors:** 

## Meeting Keynote


**Thomas Rando**


Conference Keynote Speaker

Stanford University, Stanford, CA, USA

## Coordination of Cellular Responses for Tissue Regeneration

### Thomas A Rando

#### Stanford University, Stanford, CA, USA

Tissue regeneration requires a coordinated response of cells that are essential for effective tissue repair after injury. In skeletal muscle, the cellular constituents of the regenerative response includes the initial immune cells that infiltrate the area of injury, followed by an expansion not only of the key muscle stem cells (MuSCs) that are essential for muscle regeneration but also of a diverse population of cells that contribute to effective regeneration. These include endothelial cells necessary for restoration of the tissue vasculature and mesenchymal cells, including fibroadipogenic progenitors (FAPs), essential for the restoration of the interstitial architecture of the tissue.

Much of our work on MuSCs has focused on the biology of the quiescent state and changes in that state that influence the effectiveness of the ability of MuSCs to engage in tissue repair. We have identified key regulators of MuSC quiescence, including the Notch signaling pathway and a microRNA (miR489) pathway. More recently, our studies have focused on dynamics of the quiescent state, identifying states that are either more or less “deeply” quiescent that influence the regenerative responses. One such state, which we termed G_Alert_, poises MuSCs for more rapid and effective tissue repair. Ongoing studies are examining the effects of exercise on MuSC quiescence and the impact on regeneration in aged muscle. We have identified a cyclin D1‐TGFβ axis that appears to mediate the beneficial effects of exercise on aged muscle repair. We have also begun to dissect the in vivo transcriptome of quiescent stem cells to develop a more accurate description of stem cell quiescence. These studies have revealed an unexpected level of transcriptional activity in the quiescent state, as well as some of the initial changes that occur in the transcriptome in response to activating stimuli.

We have also explored the pleiotropic actions of FAPs as mediators of either effective regeneration or as contributors to aberrant outcomes, particularly fibrosis and adiposis, in impaired regeneration as is found with age and in muscle disorders such as the muscular dystrophies. We have identified a key regulatory pathway of PDGFRα signaling that involves intronic polyadenylation that either promotes normal regeneration or enhanced fibrosis by altering the fate of FAPs during the regenerative process. More recently, we have discovered the role of a microRNA (miR206, previously thought to be muscle‐specific) in regulating the propensity of FAPs to adopt an adipogenic fate, leading to an impairment of the muscle regenerative response.

The long‐term goal of our studies is to understand the dynamic state of stem cell populations during homeostasis, to characterize the complex environment of regenerating tissue and how that influences the fate and function of stem and progenitor populations, and to discover targets that will allow restoration of normal regenerative responses in conditions such as aging and disease when regeneration is impaired.


**Meeting Session Keynote Speakers**



**Paul Robbins, PhD**


Session 1. Muscle‐Bone Interactions During Aging

University of Minnesota, Minneapolis, MN, USA

DOI: 10.002/jbm4.10257


## Cell Autonomous and Non‐autonomous Mechanisms of Musculoskeletal Aging

### Paul D Robbins, Lei Zhang, Matthew J Yousefzadeh, Kayla Lee, Tianpeng Zhang, Rafael Flores, Jing Zhao, Fernando Santiago, Luise Angelini, and Laura J Niedernhofer

#### Institute on the Biology of Aging and Metabolism, Department of Biochemistry, Molecular Biology and Biophysics, University of Minnesota, Minneapolis, MN, USA

With aging there is progressive loss of tissue homeostasis and functional reserve, leading to an impaired response to stress and an increased risk of morbidity and mortality. The loss of tissue homeostasis is generally accepted to arise as a consequence of the time‐dependent accumulation of cellular damage that can drive cellular senescence and stem cell dysfunction. Senescence is a programmed cell fate in response to numerous types of cellular stress. Senescent cells are known to play a causal role in numerous age‐related diseases and aging itself. They do so largely via their senescence‐associated secretory phenotype (SASP), which disrupts tissue homeostasis locally and drives chronic sterile inflammation systemically. Mice expressing reduced levels of the DNA repair endonuclease ERCC1‐XPF (*Ercc1*
^−/∆^ mice) accumulate oxidative DNA lesions (endogenous genotoxic stress) and thereby senescent cells ~6× faster than wild‐type (WT) mice. This causes accelerated aging. Remarkably, the location and level of senescence markers are nearly identical between *Ercc1*
^−/∆^ and aged WT mice. Treating *Ercc1*
^−/∆^ or aged WT mice with novel senolytic drugs to eradicate senescent cells attenuated age‐related organ dysfunction and histopathology, including bone, intervertebral disc, and muscle pathology. To determine which senescent cell types most potently drive aging and age‐related disease, we generated multiple tissue‐specific *Ercc1* mutant mouse strains. Deletion of *Ercc1* in a single tissue or cell type typically resulted in accelerated accumulation of senescent cells in the targeted organ and premature loss of organ function. This approach enabled us to identify which senescent cell types are most potent at driving senescence and aging in *trans*, and therefore are most critical to target with senolytic drugs. An update regarding what cell types drive systemic aging will be presented.

A key mediator of the cellular response to damage and stress is the transcription factor NF‐κB. The activity of NF‐κB is upregulated in response to different types of cellular stress and in tissues of aged organisms, making it an excellent candidate mediator of senescence, SASP, and age‐related degenerative changes. We have demonstrated previously that NF‐κB transcriptional activity is upregulated in a variety of tissues, including muscle with both natural and accelerated aging. To determine what activates NF‐κB with aging as well as to examine the role of NF‐κB activation in senescence and aging, we generated mice with accelerated aging: (1) heterozygous for NF‐κB subunit p65(RelA) and an upstream activator of IKK/NF‐κB (ATM); (2) carrying a mutation in the NEMO/IKKγ subunit unable to activated by DNA damage; and (3) deficient in TNF and IL‐1/TLR signaling. The effect of these genetic changes as well as pharmacologic inhibition of IKK/NF‐κB on senescence, stem cell function, and aging will be presented.


**Jürg A Gasser, PhD**


Session 3. Exercise‐Mediated Muscle‐Bone Transducers Novartis Institutes for BioMedical Research, Musculoskeletal Diseases, Basel, Switzerland

DOI: 10.002/jbm4.10258


## Physical Exercise, the ‘Missing Link’ Between Drug Treatment‐Induced Muscle Hypertrophy and Its Conversion into Functional Improvement?

### Jürg A Gasser PhD

#### Novartis Institutes for BioMedical Research, Musculoskeletal Diseases, Basel, Switzerland

Sarcopenia affects 2% to 5% of older adults aged 70 years and older, described as age‐associated loss of muscle mass that results in impaired muscle strength and power, adversely affecting an older person's functional capability. Typical hallmarks include slowed walking speed and difficulty with basic movements (rising from a seated position, climbing stairs, and continuous walking). The physical consequences of sarcopenia put a person at risk for falls and fractures, hospitalization, loss of independent living, and death. A substantial body of literature demonstrate the benefits of exercise, primarily resistance training, and physical activity on muscle mass, strength, and function in older adults of various levels of baseline physical function. Similarly, data showing the efficacy of increased dietary protein and other nutrients that result in the maintenance of physical function have led to revised dietary recommendations for protein and other nutrients in older people. Since not all individuals are willing or able to participate in an exercise program, there is room for pharmacological therapy in the treatment of sarcopenia.

The first generation of muscle drugs directly address the original defining characteristic of sarcopenia, the loss of muscle mass, with the expectation that a resulting muscle hypertrophy would translate to an increase in muscle strength and improved function. Muscle anabolic agents that were clinically tested include selective androgen receptor modulators, as well as myostatin, activin, and ActRII pathway antagonists. One of these treatments, bimagrumab (BYM338), is a fully human monoclonal antibody that binds activin type II receptor (ActRIIA and ActRIIB), preventing binding to their natural ligands, which negatively regulate muscle growth, including myostatin, growth and development factor 11, and activin. In clinical studies, a single dose of bimagrumab caused an increase in thigh muscle volume, measured by MRI, of ~6% after 10 weeks in healthy lean adults compared with placebo and reduced fat mass to a similar extent. A single dose of bimagrumab increases muscle mass in healthy young men similar to that achieved with 12 weeks of high‐intensity resistance training, and in sedentary middle‐aged adults, equivalent to that achieved with 9 months of jogging 12 to 20 miles per week. Reversal of atrophy in elderly people in their 70s and in a single leg casted model in healthy young men was also demonstrated.

Results from clinical trials with various muscle anabolic agents have consistently shown a range of measurable muscle hypertrophy, with limited or no success for improving muscle strength or patient physical function. It is the translation of muscle mass to improved patient function that remains the major challenge for current experimental drugs. Why does this happen? Skeletal muscle contractions require synaptic input from motor neurons and are dependent on energy metabolism. Sarcopenia is a constellation of multiple factors involving the aging neuromuscular machinery (declining motor unit number and efficiency, muscle architecture and orientation, fiber type distribution, excitation‐contraction coupling), reduced anabolic hormone levels, muscle disuse, and inflammation, driven by environmental, genetic, and behavioral factors. Blocking activin/myostatin signaling with bimagrumab induces muscle fiber hypertrophy only, in slow‐ and fast‐twitch muscles in young mice, but does not change the fiber type pattern or fiber number and has no effect on the number of motor neuron units or energy metabolism. In animals, bimagrumab worked well in the prevention of glucocorticoid‐induced muscle atrophy. It improved the recovery of skeletal muscle mass associated with steroid use and immobilization‐induced atrophy conducted in a leg‐casting model. However, bimagrumab did not prevent disuse atrophy resulting from limb casting or denervation, models where muscle contractions are very limited or absent. Taken together, nonclinical and clinical results suggest that physical exercise is critically important to translate the beneficial effect of muscle hypertrophy agents such as bimagrumab into an improvement of physical function.


**Gustavo Duque, MD, PhD, FRACP, FGSA**


Session 4. Role of Muscle and Bone Factors in Energetics and Metabolism

University of Melbourne, Melbourne, Australia

DOI: 10.002/jbm4.10259


## Beyond Energy Regulation: New Insights into Fat, Muscle, and Bone Interactions

### Gustavo Duque, MD, PhD, FRACP, FGSA

#### Australian Institute for Musculoskeletal Science (AIMSS), The University of Melbourne and Western Health, Melbourne, Australia

In older persons, the combination of osteopenia/osteoporosis and sarcopenia—known as osteosarcopenia—has been proposed as a subset of frailer individuals at higher risk of institutionalization, falls, and fractures. The pathophysiology of osteosarcopenia is the consequence of a complex set of interactions between bone, muscle, and fat. Osteosarcopenic patients have very particular clinical, biochemical, diagnostic, and functional characteristics that could be identified in clinical practice. In addition, new therapies targeting both muscle and bone, which involve fat as a new target, are being developed. In this session, the pathophysiology of osteosarcopenia will be reviewed. In addition, a clinical definition of osteosarcopenia aiming to describe the clinical, functional, and biochemical features that are unique to these patients will be presented. The use of imaging combined with functional assessments for the diagnosis of osteosarcopenia will be discussed, including novel methods to quantify bone marrow and intramuscular fat. In addition, we will analyze preventive measures and therapeutic interventions that can benefit both muscle and bone simultaneously. We intend to go over the translational aspects of sarcopenia and osteoporosis research and highlight expected outcomes from different interventions for both conditions.


**Céline Colnot**


Session 5. Muscle‐Bone Interactions in Orthopedics

Imagine Institute, Paris Descartes University, Paris, France

DOI: 10.002/jbm4.10260


## Bone‐Muscle Interactions in Bone Repair and Musculoskeletal Trauma: Role of Skeletal Muscle Mesenchymal Progenitors

### Anais Julien,^1^ Anuya Kanagalingam,^1^ Jérome Megret,^2^ Marine Luka,^1^ Mickaël Ménager, Frédéric Relaix,^3^ and Céline Colnot^1^


#### 
^1^INSERM U1163, Imagine Institute, Paris Descartes University, Paris, France; ^2^INSERM US24 ‐ CNRS UMS3633 Cytometry Platform, Paris Descartes University, Paris, France; ^3^INSERM IMRB U955, Paris Est‐Créteil University, Créteil, France

Skeletal muscle and bone exhibit great capacities to regenerate due to tissue‐specific stem cells, ie, satellite cells and skeletal stem cells from periosteum.^(1,2)^ However, bone fails to heal properly in 10% of bone injuries, and delayed healing is increased to 40% in patients with soft tissue damage associated with bone fracture. The role of skeletal muscle in bone repair is well recognized clinically, but the underlying cellular and molecular mechanisms are poorly understood. Muscle regulates the inflammatory environment of fracture, and muscle satellite cells are providing a source of growth factors for bone repair.^(3,4)^ Here we characterized skeletal muscle mesenchymal progenitors that are actively recruited at the fracture site from the adjacent skeletal muscle in response to bone injury and give rise to cartilage and bone in the fracture callus. These skeletal muscle mesenchymal progenitors are derived from a common mesenchymal lineage marked by Prx1 with bone marrow stromal/stem cells and periosteal cells. Single‐cell RNA‐seq analyses identified five subpopulations of skeletal muscle mesenchymal progenitors and their response to injury. In a new murine model of musculoskeletal trauma where tibial fractures were induced and combined with a mechanical injury to skeletal muscles surrounding the tibia, the contribution of skeletal muscle mesenchymal progenitors is decreased. Further single‐cell RNA‐seq analyses revealed an impaired fibrotic response of skeletal muscle mesenchymal progenitors during the early stage of repair in the polytrauma environment compared with fracture alone. This is followed by abnormal callus organization with the presence of unresorbed cartilage and fibrosis leading to nonunion. Fibrotic tissue accumulating within the callus after polytrauma is produced by skeletal muscle mesenchymal progenitors and can be reduced by treating mice with the pan‐kinase inhibitor Imatinib. Skeletal muscle thus plays a central role during bone repair as a source of mesenchymal progenitors producing cartilage and bone required for repair and as a mediator of initial fibrotic response and fibrotic remodeling. The findings suggest that new pharmacological and cell‐based approaches can be developed to improve musculoskeletal regeneration by targeting skeletal muscle adjacent to bone.

## References


Lepper C, et al. Development. 2011;138(17):3639–46.Duchamp de Lageneste O, et al. Nat Commun. 2018;22;9(1):773.Abou‐Khalil R, et al. JBMR. 2014;29(2):304–15.Abou‐Khalil R, et al. Stem Cell. 2015;33(5):1501–11.



**Silvia Salinas Blemker**


Session 6. Biomechanical Relationships Between Bone and Muscle

University of Virginia, Charlottesville, VA, USA

DOI: 10.002/jbm4.10261


## The Complex Interplay Between Musculoskeletal Biomechanics and Physiology: Insights Gained From Coupling Experiments With Multi‐Scale Computational Models

### Silvia Salinas Blemker

#### University of Virginia, Charlottesville, VA, USA

From a basic science perspective, there as a vast and deep body of literature describing the underpinnings of the biology and mechanics of the musculoskeletal system. However, the translation of these basic understandings to medicine is highly limited because it is challenging to intuit how all findings from physiology and mechanics relate and interact, which hinders innovation and improvement in treatment approaches. The goal of the Multi‐Scale Muscle Mechanophysiology (“M3”) Lab's research is to develop and experimentally validate multi‐scale computational models of the musculoskeletal system and apply these models to answering questions related to a variety of clinical problems. In this presentation, I will describe these approaches and provide some recent examples of how computational models of muscle have led to new insights into the interplay between biomechanics and physiology.


**Jose Millan, PhD**


Session Keynote Speaker

Session 7. Muscle‐Bone Interactions in Genetic Diseases

Sanford Burnham Prebys, Medical Discovery Institute, La Jolla, CA, USA

DOI: 10.002/jbm4.10262


## New Insights into the Pathophysiology of Hypophosphatasia

### José Luis Millán, PhD

#### Sanford Burnham Prebys Medical Discovery Institute, La Jolla, CA, USA

Hypophosphatasia (HPP) is the heritable rare disease that results from *ALPL* gene mutations leading to deficient activity of the tissue‐nonspecific alkaline phosphatase isozyme (TNAP). HPP features rickets or osteomalacia and early loss of teeth. These skeletal and dental manifestations are caused by the accumulation of extracellular inorganic pyrophosphate (PP_i_), a physiological substrate of TNAP and a potent mineralization inhibitor. Additionally, phosphorylated osteopontin (OPN), another potent mineralization inhibitor, also accumulates, further restricting the degree of extracellular matrix mineralization. Understanding this pathophysiology has provided the rationale for the current therapeutic intervention for HPP using recombinant mineral‐targeted TNAP for enzyme replacement. Severely affected HPP patients, as well as *Alpl*
^*−/−*^ mice, suffer from severe seizures that herald a lethal outcome. The seizures are partly explained by inadequate availability of pyridoxal phosphate, a physiological substrate of TNAP that is a co‐factor in the synthesis of neurotransmitters by neuronal cells, but aberrant P2X7 signaling in the central nervous system is also part of the pathophysiology. Other features of HPP are not yet well understood, such as what pathophysiological mechanisms lead to the development of craniosynostosis, nephrocalcinosis, muscle weakness, inflammation, and pain. During my presentation, I will argue that these poorly understood manifestations of HPP are caused at least in part by local changes in the ATP/adenosine ratio and levels of endotoxins (lipopolysaccharides) as a result of deficient TNAP activity, leading to affected cell behavior and tissue homeostasis.


**Sarah Dallas**


Session Keynote Speaker

Session 8. Role of Soluble Factors in Muscle‐Bone Interactions

University of Missouri–Kansas City

Kansas City, MO, USA

DOI: 10.002/jbm4.10263


## Extracellular Vesicle‐Mediated Cell–Cell Communication between Osteocytes and Osteoblasts and Potential Role in Muscle‐Bone Cross‐Talk

### Sarah L Dallas

#### University of Missouri, Kansas City, MO, USA

Accumulating evidence suggests that signaling cross‐talk occurs between bone and muscle via circulating and local mediators. This type of cross‐talk may coordinate the beneficial effects of exercise in both tissues and also the degenerative changes in muscle and bone that occur with aging. A recent paradigm in cell–cell communication involves the shedding of exosomes/extracellular vesicles (EV) from cells that deliver their cargo of proteins, mRNAs, and miRNAs to target cells, thereby altering their function. EV can work in a paracrine fashion but can also be shed into the circulation to modulate the function of cells at distant sites. Their potential role as mediators of muscle‐bone cross‐talk is not well defined.

Using primary bone cell cultures from mice expressing a membrane‐targeted GFP in osteocytes (Dmp1‐mGFP), we showed that embedding osteocytes shed EV, some of which are incorporated into the extracellular matrix and some of which may signal to nearby osteoblasts. Intravital imaging in Dmp1‐mGFP mice injected intravenously with fluorescent dextran showed osteocytes extending dendrites toward blood vessels and releasing EV near the lumen. GFP‐positive EV were detected in blood from these mice, suggesting that osteocytes release EV into the circulation and indicating their potential to affect distant organs. To examine the role of osteocyte EV in regulating osteoblast function, IDG‐SW3 cells were used as a model of late osteocyte differentiation for EV isolation. Western blotting and proteomic analysis of EV from day 28 IDG‐SW3 cells (osteocyte‐enriched) revealed an EV proteome of >2000 proteins that was enriched for known exosome markers and contained osteocyte markers E11, PHEX, MEPE, Dmp1, sclerostin, and RANKL and proteins involved with membrane fusion/exocytosis, motility/neurite outgrowth, mineralization, and ECM assembly. Interestingly, the composition of the EV was altered by PTH treatment, including downregulation of sclerostin and upregulation of RANKL. Profiling of miRNAs (Affymetrix 4.0) in control and PTH‐treated IDG‐SW3 cells identified >500 miRNAs in the EV and >650 in the cell layer with 105 miRNAs increased in PTH versus control EV. Principal component analysis showed differential miRNA partitioning between the EV and cell layer. Treatment of early undifferentiated IDG‐SW3 cells (osteoblast‐like) with EV from late differentiated IDG‐SW3 cells (osteocyte‐enriched) induced expression of Dmp1 and RANKL and induced mineralization, suggesting promotion of osteoblast‐to‐osteocyte transition. Interestingly, treatment with EV from PTH‐treated IDG‐SW3 cells downregulated SOST expression in “naïve” cells that were not exposed to PTH, suggesting that EV can propagate PTH responses to other cells. Next, the potential role of EV in muscle‐bone cross‐talk was examined. Live‐cell imaging showed EV release by C2C12 myogenic cells, with C2C12 myoblasts releasing twofold more EV than C2C12 myotubes. Treatment with C2C12 myotube but not myoblast EV increased Wnt/β‐catenin signaling in MLO‐Y4 osteocyte‐like cells, which has a known role in maintaining osteocyte viability. Confocal microscopy showed internalization of C2C12‐EVs by MLO‐Y4 cells, which altered their gene expression and conferred expression of muscle‐related mRNAs MYH, MyoG, and MyoD. Together, these data support an important role for osteocyte EV in regulation of bone cell function and suggest a role for EV in muscle‐bone cross‐talk.


**Roger Fielding, PhD**


Session Keynote Speaker

Session 9. Nutritional Mediators of Muscle‐Bone Interactions

Tufts University, Boston, MA, USA

DOI: 10.002/jbm4.10264


## Nutritional Mediators of Muscle‐Bone Interactions

### Roger A Fielding, PhD

#### Associate Center Director, Jean Mayer USDA Human Nutrition Research Center on Aging at Tufts University; Director and Senior Scientist, Nutrition, Exercise Physiology, and Sarcopenia Laboratory; Professor of Nutrition and Medicine; Friedman School of Nutrition Science and Policy, Tufts University School of Medicine; Associate Director, Boston Claude D Pepper Older Americans Independence Center

The age‐related loss of skeletal muscle mass and function, sarcopenia, is associated with well‐characterized functional limitations, physical disability, and distal clinically relevant outcomes such as falls, fractures, and death. Underlying these age‐related changes are physiological changes in the force/power‐generating capacity of skeletal muscle that appear to be driven by changes in skeletal contractile protein function, metabolic derangements, and alterations in neuromuscular activation. Biologically relevant age‐associated changes in skeletal muscle include alterations in gene transcription, mitochondrial stability, anabolic capacity, and metabolic flexibility. Underlying molecular targets have been identified in skeletal muscle that are potential sites for the development of therapeutic interventions. Data from observational studies suggest that both adequate nutrition and increased physical activity appear to attenuate or reverse several of the age‐related changes in skeletal muscle function. However, the data on nutritional and physical activity interventions on muscle function in mobility‐limited older adults are more complex. In my presentation, I will review the current literature examining the potential mechanisms by which nutritional supplementation (dietary protein, vitamin D, omega‐3 fatty acids) may improve skeletal muscle function and metabolism. I will also provide data from our recent trials that have addressed the influence of physical activity and nutrition on age‐related changes in skeletal muscle performance and physical functioning/disability in mobility‐limited older adults. Emerging evidence suggests that some dietary factors interact with the anabolic stimulus of increased physical activity to alter skeletal muscle mass, bone mass, and body fat distribution. These findings have important implications for restoring and improving physical functioning among older adults.


**Mary Leonard, PhD**


Session Keynote Speaker

Session 10. Muscle‐Bone Interactions in Pediatrics

Stanford University, Stanford, CA, USA

DOI: 10.002/jbm4.10265


## Bone‐Muscle Interactions in Healthy Children and Those With Chronic Disease

### Mary Leonard, PhD

#### Arline and Pete Harman Professor, Chair of the Department of Pediatrics at Stanford University School of Medicine and the Adalyn Jay Physician in Chief at Lucile Packard Children's Hospital Stanford. Stanford University, Stanford, CA, USA

Skeletal development is characterized by sex‐, race‐ and maturation‐specific increases in trabecular bone volume fraction (BV/TC), cortical bone mineral density (BMD), cortical dimensions, and bone failure load. Modeling on the periosteal and endosteal surfaces produce changes in cortical geometry that impact lifelong fracture risk. As muscle mass and strength increase during growth, bones adapt by increasing cortical dimensions and strength. Similarly, greater physical activity is associated with greater gains in trabecular BV/TV and periosteal circumference. The capacity of trabecular and cortical bone to respond to mechanical loading is greatest during childhood. We've demonstrated that adjustment for sex and race differences in muscle size attenuated but did not eliminate sex and race differences in cortical dimensions in children and young adults. The associations between muscle and bone outcomes did not differ according to sex or race, suggesting similar mechanostat set points. Last, in otherwise healthy adolescents, obesity was associated with advanced skeletal maturity, greater muscle mass, and markedly greater cortical section modulus in the tibia. In multivariate models, greater tibia cortical section modulus in obese adolescents was attributable to advanced skeletal maturation, greater muscle area, and greater strength, whereas less moderate to vigorous physical activities offset the positive effects of these covariates. The impact of obesity was less evident in the nonweight‐bearing radius.

Given the strong associations between muscle mass and bone strength, investigators have advocated for the assessment of bone relative to muscle in children with chronic diseases. We've demonstrated that chronic inflammatory diseases (Crohn's disease, juvenile inflammatory arthritis, and bone marrow transplantation) were associated with significant deficits in muscle mass and cortical dimensions. In these cross‐sectional studies, adjustment for muscle mass significantly attenuated the diseases’ effects on cortical dimensions compared with healthy controls. More recent longitudinal studies suggested that the relations between changes in muscle mass and changes in cortical dimensions varied across diseases. For example, in pediatric renal transplant recipients, deficits in muscle mass resolved within months after transplantation; however, cortical bone deficits persisted. This may be explained by poor muscle quality (less power relative to muscle mass) in chronic kidney disease. In contrast, children with Crohn's disease demonstrated marked gains in calf muscle mass and cortical bone after treatment with anti‐TNF‐α biologics and the gains in cortical area relative to gains in muscle area did not differ between Crohn's disease patients and healthy controls. Importantly, our 12‐month randomized double‐blind placebo controlled trial of 10 minutes’ daily exposure to low‐magnitude mechanical stimuli in children with Crohn's disease demonstrated a modest effect on quantitative CT (QCT) measures of spine BMD but no effect on any DXA or peripheral QCT measures of bone or body composition.

Differences in the skeletal response to loading may be due to disease effects on muscle force relative to muscle mass (muscle‐specific effects), decreased physical activity, adverse cytokine or disease effects on the mechanosensing osteocytes, abnormalities in insulin‐like growth factor 1 (IGF‐1), or glucocorticoid effects to inhibit bone formation. Future studies are needed to determine if physical activity or biomechanical interventions will increase muscle mass and bone density and dimensions in children with chronic diseases and to identify diseases with the greatest potential to respond. Speakers in alphabetical order.


**Abdullah Alshudukhi, PhD (c)**


Wright State University, Dayton, OH, USA

Relevant Session: Role of Muscle and Bone Factors in Energetics and Metabolism

DOI: 10.002/jbm4.10266


## Lipin‐1 Regulates Bnip3–Mediated Mitophagy in Glycolytic Muscle

### AA Alshudukhi,^1^ D Huang,^1^ A Jama,^1^ H Ren,^1^ JD Smith,^2^ J Zhu,^2^ QJ Wang,^2^ and KA Esser^3^


#### 
^1^Department of Biochemistry and Molecular Biology, Wright State University, Dayton, OH, USA; ^2^Cardiovascular Research Center, Department of Biosystems and Agricultural Engineering, and Department of Ophthalmology and Visual Sciences, University of Kentucky, Lexington, KY, USA; ^3^Myology Institute, College of Medicine, University of Florida, Gainesville, FL, USA

Autophagy of mitochondria (mitophagy) is essential for maintaining muscle mass and healthy skeletal muscle. Patients with heritable phosphatidic acid phosphatase lipin‐1–null mutations present with severe rhabdomyolysis and muscle atrophy in glycolytic muscle fibers, which are accompanied with mitochondrial aggregates and reduced mitochondrial cytochrome c oxidase activity. However, the underlying mechanisms leading to muscle atrophy as a result of lipin‐1 deficiency are still not clear. In this study, we found that lipin‐1 deficiency in mice is associated with a marked accumulation of abnormal mitochondria and autophagic vacuoles in glycolytic muscle fibers. Our studies using lipin‐1–deficient myoblasts suggest that lipin‐1 participates in B‐cell leukemia (BCL)‐2 adenovirus E1B 19 kDa protein–interacting protein 3 (Bnip3)–regulated mitophagy by interacting with microtubule associated protein 1A/1B‐light chain (LC)3,which is an important step in the recruitment of mitochondria to nascent autophagosomes. The requirement of lipin‐1 for Bnip3–mediated mitophagy was further verified in vivo in lipin‐1–deficient green fluorescent protein‐LC3 transgenic mice (lipin‐12/2‐GFP‐LC3). Finally, we showed that lipin‐1 deficiency in mice resulted in defective mitochondrial adaptation to starvation‐induced metabolic stress and impaired contractile muscle force in glycolytic muscle fibers. In summary, our study suggests that deregulated mitophagy arising from lipin‐1 deficiency is associated with impaired muscle function and may contribute to muscle rhabdomyolysis in humans.


**Ahmed Al Saedi, PhD**



**Early Investigator Awardee*


Oral Presentation – see Session 7 for abstract.

DOI: 10.002/jbm4.10267


## Severely Decreased Bone Formation and Muscle Quality in the Winnie Mouse Model of Inflammatory Bowel Disease (IBD)

### Ahmed Al Saedi,^1,2^ Shilpa Sharma,^1,2^ Lulu Chen,^4^ Ebrahim Bani Hassan,^1,2^ Rajaraman Eri,^5^ Kulmira Nurgali,^1,2,3^ and Gustavo Duque^1,2^


#### 
^1^Department of Medicine–Western Health, The University of Melbourne, St. Albans, Australia; ^2^Australian Institute for Musculoskeletal Science (AIMSS), The University of Melbourne and Western Health, St. Albans, Australia; ^3^Institute for Health and Sport, Victoria University, Melbourne, Australia; ^4^Department of Anatomy, Histology, and Embryology, Nanjing Medical University, Nanjing, Jiangsu, People's Republic of China; ^5^School of Health Sciences, University of Tasmania, Launceston, Australia

Although osteoporosis and sarcopenia commonly afflict patients with inflammatory bowel disease, the mechanisms of bone/muscle loss in these subjects remain poorly understood. A major limitation to investigate those changes in bone mass and muscle mass has been the lack of an appropriate animal model for IBD. In this study, we characterized the bone phenotype and muscle analysis of the Winnie mouse model, which carries a mutation in the Muc2 gene and closely replicates the symptoms and pathophysiology of IBD and produces high levels of gut‐derived serotonin (GDS), a potent inhibitor osteoblastogenesis. Six‐, 14‐, and 21‐week‐old Winnie mice were compared with age‐ and sex‐matched control C57BL/6 mice (WT). We assessed bone quality properties by static and dynamic bone histomorphometry and microCT analyses and muscle staining and analysis. Despite similar body weight, bone formation in Winnie mice was severely decreased in trabecular surfaces at 14 and 21 weeks, respectively, compared with WT (bone formation rate/bone surface −20%, −28%, *p* < 0.05) and mineral apposition rate (MAR; 44% at 14 weeks, 46% at 21 weeks, μm/d, *p* < 0.05). Osteoblast number (N.Ob) was significantly lower in Winnie mice compared with WT (−42% at 14 weeks, −54% at 21 weeks, *p* < 0.001). Similarly, total collagen BV/TV (−17% at 14 weeks, −19% at 21 weeks) and collagen‐I (−9% at 14 weeks, −7% at 21 weeks) were significantly reduced in the Winnie group. In contrast, osteoclast number (N.Oc) was significantly higher compared with WT mice (+59.9% at 14 weeks, +38% at 21 weeks, *p* < 0.001). Osteoid volume/bone surface OV/BS was significantly lower in Winnie mice compared with WT (28% at 14 weeks, 23.2% at 21 weeks, *p* < 0.01). Furthermore, 3‐point bending showed lower mean failure force (MN) in Winnie mice (−20% at 14 weeks, −49% at 21 weeks, *p* < 0.05). Similarly, with yield strength (MPa) (21.3 at 14 weeks, 27.5 at 21 weeks, *p* < 0.05). No differences in these parameters were noticed in Winnie mice versus WT at 6 weeks. Furthermore, microCT analysis of the distal femoral metaphysis showed that Winnie mice had significantly lower bone content (−23%), total bone density, cortical and trabecular bone content, cortical bone area, and periosteal and endocortical circumferences compared with WT at 14 weeks and 21 weeks. Skeletal muscle phenotyping showed the total proportion of oxidative fibers in the muscle was greater in WT compared with Winnies (18% at 14 weeks, 27.5 at 21 weeks, *p* < 0.05). In summary, this is the first study performing a full bone phenotyping and muscle analysis in a mouse model of IBD, which could open avenues for understanding the mechanisms involved in IBD‐related bone/muscle loss. The predominant compromise of bone/muscle activity is indicative of mechanisms other than inflammation, which could involve high levels of GDS, thus providing therapeutic potentials for bone disorders and muscle atrophy in this population.


**Keith Avin, PhD**


Indiana University, Indianapolis, IN, USA

Relevant Session: Musculoskeletal Health in Aging and Disease

DOI: 10.002/jbm4.10268


## Voluntary Wheel Running Has Beneficial Effects in a Rat Model of CKD‐Mineral Bone Disorder (CKD‐MBD)

### KG Avin, MR Allen, NX Chen, S Srinivasan, KD O'Neill, AD Troutman, G Mast, EA Swallow, MB Brown, JM Wallace, TA Zimmers, SJ Warden, and SM Moe

#### 
^1^Division of Nephrology, Indiana University School of Medicine, Indianapolis, IN, USA; ^2^Department of Physical Therapy, Indiana University School of Health and Human Sciences, Indianapolis, IN, USA; ^3^Roudebush Veterans Affairs Medical Center, Indianapolis, IN, USA; ^4^Department of Anatomy and Cell Biology, Indiana University School of Medicine, Indianapolis, IN, USA; ^5^Department of General Surgery, Indiana University School of Medicine, Indianapolis, IN, USA

Individuals with chronic kidney disease (CKD) exhibit impaired musculoskeletal health, which contributes to their elevated morbidity and mortality. We tested the hypothesis that voluntary wheel running would improve musculoskeletal health in a CKD rat model. Cy/+ (CKD) rats with spontaneous progressive cystic kidney disease and normal littermates (NL) at 25 weeks of age (stage 2 to 3 CKD) had access to a voluntary running wheel or standard cage conditions for 10 weeks. Outcomes included serum biochemistry, tissue weight, voluntary grip strength, maximal aerobic capacity (VO2max), body composition, bone micro‐CT and mechanics. Wheel running improved serum biochemistry (decreased creatinine, phosphorous, and parathyroid hormone); improved muscle strength; increased time‐to‐fatigue (for VO2max); reduced cortical porosity and improved bone microarchitecture; reduced kidney cystic weight; and reduced left ventricular mass index in CKD rats. Voluntary wheel running resulted in multiple beneficial systemic effects in CKD rats and improved their physical function. Studies examining exercise interventions in patients with CKD are warranted.


**Julian Andres Balanta Melo, PhD (c)**



**Early Investigator Awardee*


Universidad de Chile, Recoleta, Santiago, Chile

Relevant Session: Biomechanical Relationships Between Muscle and Bone

DOI: 10.002/jbm4.10269


## Masseter Muscle Atrophy Leads to Osteocyte Apoptosis and Loss of Bone Mass in the Mandibular Condyle in Mice Treated with Botulinum Toxin Type A

### Julián Balanta‐Melo,^1,2,6^ Viviana Toro‐Ibacache,^1,3,4^ María Torres‐Quintana,^5^ Kornelius Kupczik,^3,6^ Lilian Plotkin,^7^ and Sonja Buvinic^1^


#### 
^1^Institute for Research in Dental Sciences, Faculty of Dentistry, Universidad de Chile, Chile; ^2^School of Dentistry, Universidad del Valle, Colombia; ^3^Center for Quantitative Analysis in Dental Anthropology, Faculty of Dentistry, Universidad de Chile, Chile; ^4^Department of Human Evolution, Max Planck Institute for Evolutionary Anthropology, Germany; ^5^Department of Pathology and Oral Medicine, Faculty of Dentistry, Universidad de Chile, Chile; ^6^Max Planck Weizmann Center for Integrative Archaeology and Anthropology, Max Planck Institute for Evolutionary Anthropology, Germany; ^7^Department of Anatomy and Cell Biology, Roudebush Veterans Administration Medical Center, and Indiana Center for Musculoskeletal Health, Indiana University School of Medicine, USA

Osteocytes are bone cells specialized in mechanotransduction. These cells drive the local bone remodeling through their apoptosis during altered biomechanical conditions. In adult mice, the unilateral masseter atrophy induced by botulinum toxin type A (BoNTA) modifies the masticatory muscles balance, resulting in mandibular condyle bone loss after 14 days. Here we hypothesize that BoNTA‐induced masseter atrophy leads to osteocyte apoptosis, which precedes the reduction in mandibular condyle bone quality 14 days after intervention. Thirty‐eight adult male BALB/c mice were used. At day 0, all mice received one BoNTA injection in the right masseter and saline in the opposite side. IG9402, a bisphosphonate analog that does not inhibit osteoclast activity, served as osteocyte apoptosis inhibitor (0.6 mg/kg/d) and its vehicle as control. Mice were randomly distributed: BoNTA/2d (*n* = 5), BoNTA/7d (*n* = 5), BoNTA/14d + vehicle (*n* = 15) and BoNTA/14d + IG9402 (*n* = 13). All animals were euthanized after 2 days, 7 days, or 14 days. Immunohistochemistry for cleaved‐caspase‐3 or RANKL and TRAP staining were performed in condyles from BoNTA/2d and BoNTA/7d groups. The condyles from BoNTA/14d groups were analyzed with microCT using four bone histomorphometric parameters: bone volume fraction (BV/TV), trabecular thickness (Tb.Th), specific bone surface (BS/BV), and bone mineral density (BMD). All procedures under ethical committee approval. In the experimental condyles from BoNTA/7d, the total number of osteocytes was 20% lower and the proportion of apoptotic osteocytes were 11.5% higher than those from BoNTA/2d. Also, at day 7, the number of osteoclasts (*p* < 0.001) and the osteoclast surface/bone surface (*p* < 0.01) were significantly increased. No difference in the RANKL‐positive osteocytes was found. At day 14, the experimental condyles from BoNTA/14d + IG9402 exhibited larger Tb.Th (*p* < 0.01), higher BMD (*p* < 0.01), and lower BS/BV (*p* < 0.05) than samples from BoNTA/14d + vehicle. Additionally, the difference between BoNTA and saline sides were significantly reduced for all bone parameters in the IG9402‐injected mice compared with vehicle‐treated mice (*p* < 0.05). Our results suggest that osteocyte apoptosis precedes the bone loss in the mature mandibular condyle during the BoNTA‐induced masseter atrophy, characterized 14 days after intervention.


**Jessica Berthiaume, PhD**


Indiana University, Indianapolis, IN, USA

Relevant Session: Muscle‐Bone Interactions in Genetic Diseases

DOI: 10.002/jbm4.10270


## Musculoskeletal Alterations in a Mouse Model of Alzheimer's Disease

### Jessica M Berthiaume, Vidyani Suryadevara, Michael Kluppel, Ruben Vidal, and Monte S. Willis

#### Department of Pathology and Laboratory Medicine, Indiana Center for Musculoskeletal Health, Indiana University, Indianapolis, IN, USA

Alzheimer's disease (AD) is a progressive neurodegenerative disease clinically manifested as a loss of cognitive function. At the cellular level, AD is characterized by β‐amyloid (Aβ) plaques (misfolded proteins that aggregate in the extracellular space) and intracellular tau accumulation. Together, these proteins and their aggregates drive neuronal dysfunction, synapse loss, and cell death. Neuronal Aβ accumulation can occur asymptomatically in AD patients for years, but the cognitive decline appears to closely parallel tau deposition. Protein‐driven pathologies are a recognized phenomenon in various organ systems, and recent retrospective studies have identified an elevated risk for heart failure and accelerated sarcopenia in AD patients compared with age‐matched controls. Pathology involving the heart, skeletal muscle, and bone have not been systematically investigated in patients or AD mouse models. To address this, we assessed a mouse model with a tau (MAPT) point mutation P301S (MAPT P301S, under the control of the prion promoter) that exhibits neurodegeneration by 8 to 10 months of age. We hypothesized that these mice would exhibit musculoskeletal defects. Whole‐body analysis of MAPT P301S mice by DXA demonstrated significant alterations in lean and fat mass compared with wild‐type controls (19.4 versus 23.9 g, and 2.7 versus 6.0 g, respectively, *p* < 0.05). In agreement with diminished lean mass, gravimetric measurement of the gastrocnemius muscle in MAPT P301S mice showed a significant decrease compared with wild‐type (7.5 versus 9.1 normalized to tibia length, *p* < 0.05). However, MAPT P301S mice did not have altered bone density (0.05 versus 0.05 g/cm^2^) or content (0.47 versus 0.46 g) as assessed by DXA. Conscious echocardiographic analysis of MAPT P301S mice identified a significant decrease in systolic function compared with wild‐type mice (74.9 versus 83.9 ejection fraction %, *p* < 0.05) in addition to reduced ventricular mass and wall thickness. Together, these findings illustrate that the heart, skeletal muscle, and fat are pathological targets in the MAPT P301S mouse and this model can be used to study the mechanisms of disease in AD and further delineate musculoskeletal effects.


**Andrea Bonetto, PhD**


Indiana University, Indianapolis, IN, USA

Relevant Session: Muscle‐Bone Interactions in Cancer

DOI: 10.002/jbm4.10271


## ACVR2B Antagonism Restores Skeletal Muscle Mass and Cardiac Function in Metastatic Colorectal Cancer Cachexia

### Joshua R Huot,^1^ Leah J Novinger,^2^ Fabrizio Pin,^3^ Alyson L Essex,^3^ Alexander J Jones,^2^ Monte S Willis,^3,4,5,6^ and Andrea Bonetto^1,2,4,5,6^


#### 
^1^Department of Surgery; ^2^Department of Otolaryngology ‐ Head and Neck Surgery; ^3^Department of Anatomy and Cell Biology; ^4^Department of Pathology; ^5^Indiana Center for Musculoskeletal Health; ^6^Simon Cancer Center, Indiana University School of Medicine, Indianapolis, IN, USA

Advanced colorectal cancer (CRC) is frequently accompanied by the development of liver metastases and muscle depletion, ie, cachexia. Cachexia also associates with cardiac malfunctioning and this condition was found to predict increased morbidity and mortality in patients with cancer. The signaling mediated by the activin receptor type 2B (ACVR2B) has been associated with muscle wasting in several disease states, and its inhibition has proven beneficial in restoring muscle mass and prolonging survival in cachexia, as well as in preserving cardiac function in aging. Unfortunately, cachexia remains understudied and currently has no cure. Here we aimed to generate and characterize a new model of CRC and to investigate whether systemic blockade of the ACVR2B signaling could preserve skeletal muscle mass and cardiac function. To this extent, NSG male mice (8 weeks old) were injected intrasplenically with 2.5x105 HCT116 human CRC cells to mimic hepatic dissemination of cancer, while sham‐operated animals received saline (*n* = 5–8/group). Sham and tumor‐bearing mice were administered ACVR2B/Fc (10 mg/Kg), a synthetic peptide inhibitor of ACVR2B, once weekly, intraperitoneally. Significant loss of body weight (−7%, *p* < 0.05), as well as marked reductions in skeletal muscle mass (quadriceps: −23%, *p* < 0.001) and muscle strength (−21%, *p* < 0.01) were observed in HCT116 hosts versus sham. Tumor hosts also showed decreased heart size (−11%, *p* < 0.05), along with impaired cardiac function (EF%: 86% in sham, 72% in HCT116, *p* < 0.001; FS%: 54% in sham, 40% in HCT116, *p* < 0.001). On the other hand, administration of ACVR2B/Fc completely preserved skeletal muscle mass (quadriceps: +31%, *p* < 0.001) and strength (+29%, *p* < 0.001) in HCT116 hosts. Despite no beneficial effects on heart size, cardiac function was also maintained in the HCT116‐bearing mice receiving ACVR2B/Fc (EF%: 86% in HCT116, *p* < 0.001; FS%: 53% in HCT116, *p* < 0.001). Interestingly, bone mass was only modestly affected by tumor growth and ACVR2B/Fc promoted overall increase in bone mass. Here we showed that ACVR2B antagonism by ACVR2B/Fc treatment fully preserves skeletal muscle mass and strength, as well as cardiac function in a new model of metastatic CRC. Our observations further consolidate the idea that ACVR2B signaling represents a promising therapeutic target for the treatment of muscle and cardiac deficits in cancer cachexia.


**Iris Boraschi‐Diaz, PhD**



**Early Investigator Awardee*


Shriners Hospital for Children, Montreal, Canada

DOI: 10.002/jbm4.10272


## Combination Treatment of Novel ActRIIB Ligand Trap and Zolendronate Improves Bone‐Muscle Proprieties in Osteogenesis Imperfecta

### Iris Boraschi‐Diaz and Frank Rauch

#### Shriners Hospital for Children‐Canada, Montreal, Canada

Osteogenesis imperfecta (OI), caused by mutations disturbing the production or processing of the collagen type I protein, is characterized by fragile bones and low muscle mass and function. Activin A and myostatin, members of the TGF‐β superfamily, are involved in an important role in the control of muscle mass and in muscle‐bone communication. We investigated activin A/myostatin signaling in a mouse model of severe dominant OI, Col1a1Jrt/+ mouse (*n* = 8 mice/group) and the effect of activin A/myostatin inhibition by a soluble activin receptor IIB trap, ACE‐2494 (10 mg/kg twice a week), in combination with zolendronate (0.05 mg/kg three times per week), on bones and muscles in 4‐week‐old male mice. Previously our group has shown that compared with wild‐type mice, Col1a1Jrt/+ mice had elevated TGF‐β signaling in bone and muscle tissue. ACE‐2494 treatment of Col1a1Jrt/+ mice resulted in 80% increase in muscle mass (*p* < 0.0001), bone length was increased 2% to 4%, but cortical thickness and the mechanical proprieties of the femur were not improved. Therefore, to improve these results we decided to combine this therapy with zolendronate. The combination treatment resulted in the observed gain in muscle mass and significant improvement in bone length but also in an improvement in cortical thickness of 4% (*p* < 0.0001) and bone mass by 200% (*p* < 0.0001). Therefore, we can conclude that activin A/myostatin ligand trap ACE‐2494 is effective in stimulating muscle mass and bone length diaphyseal, and in combination with zolendronate, we can improve in addition the bone mass and the cortical thickness phenotype observed in dominant OI.


**Marco Brotto, PhD**


University of Texas at Arlington, Arlington, TX, USA

DOI: 10.002/jbm4.10273


## Lipidomic and Metabolomic Profiling and Quantification in Women with Low and High BMD: Probing for Early Serum Metabolic Biomarkers for Osteoporosis Risk

### Marco Brotto, PhD

#### University of Texas at Arlington, Arlington, TX, USA

Osteoporosis is a metabolic bone disease with reduced bone mineral density (BMD) to result in increased risk of bone fragility and fractures. The peak BMD achieved and maintained by individuals at ages of 20 to 40 years has been reported as a powerful predictor of postmenopausal (PM) osteoporosis. The purpose of this study was to compare the lipidomic and metabolomic profiles of young white women with low and high BMD levels and to identify potential biomarkers that may enable early diagnosis for the risk of PM osteoporosis. Serum samples from 136 white women aged 21 to 41 years, with low and high *T*‐score (hip) values, were enrolled in this study. Since a strong positive correlation was observed between BMI levels and BMD levels (Spearman's correlation 0.638, *p* = 6.5 × 10–17), these participants were further grouped as normal BMI (18.5–24.9 kg/m^2^, 82 subjects) and high BMI (≥25.0 kg/m^2^, 51 subjects). Liquid chromatography‐mass spectrometry methods, including a method for profiling a total of 158 essential polyunsaturated fatty acids–derived lipid mediators (LMs) and newly developed methods for quantifying 18 key individual LMs and 5 isomeric aminobutyric acids, including γ‐aminobutyric acid (GABA) and β‐aminoisobutyric acid (BAIBA), were employed in analysis. In normal BMI subjects, serum concentrations of 8‐HDoHE were 194.3 μM and 147.1 μM in high and low BMD group, respectively, suggesting a significantly reduced 8‐hydroxyl metabolite of docosahexaenoic acid (DHA) as bone mineral density decreases (*p* = 0.020). Similar results were also observed with two other DHA metabolites, 10,17‐DiHDoHE and 7‐HDoHE. The concentration of endocannabinoid‐like compound oleoylethanolamine (OEA), which can activate peroxisome proliferator‐activated receptors, was significantly higher in the low BMD group (1.5 μM in high BMD versus 2.3 μM in low BMD, *p* = 0.0098), potentially suggesting that OEA is associated with bone formation. Moreover, correlation analysis showed a positive correlation between both GABA (*p* = 0.0055) and BAIBA (*p* = 0.017) and physical activity in the female subjects studied. To our knowledge, our study is the first one to implicate that both circulating bioactive lipids and amino acid metabolites have important implications for bone health and disease and could be applicable for the early detection of risk of osteoporosis development. Understanding their exact function could lead to earlier diagnosis and improved treatment for bone related diseases.


**Evan Buettmann, PhD**



**Early Investigator Awardee*


Virginia Commonwealth University, Richmond, VA, USA

DOI: 10.002/jbm4.10274


## Osteocytes Exposed to Simulated Microgravity Promote Myoblast Differentiation

### Evan Buettmann,^1^ Divya Krishna,^1^ Michael Friedman,^1^ Camilla Maroni,^1,2^ Chris Brunkhorst,^1^ Yue Zhang,^1^ and Henry Donahue^1^


#### 
^1^Virginia Commonwealth University, Richmond, VA, USA; ^2^D'Annunzio University, Chieti, Italy

**Figure 1 jbm410257-fig-0001:**
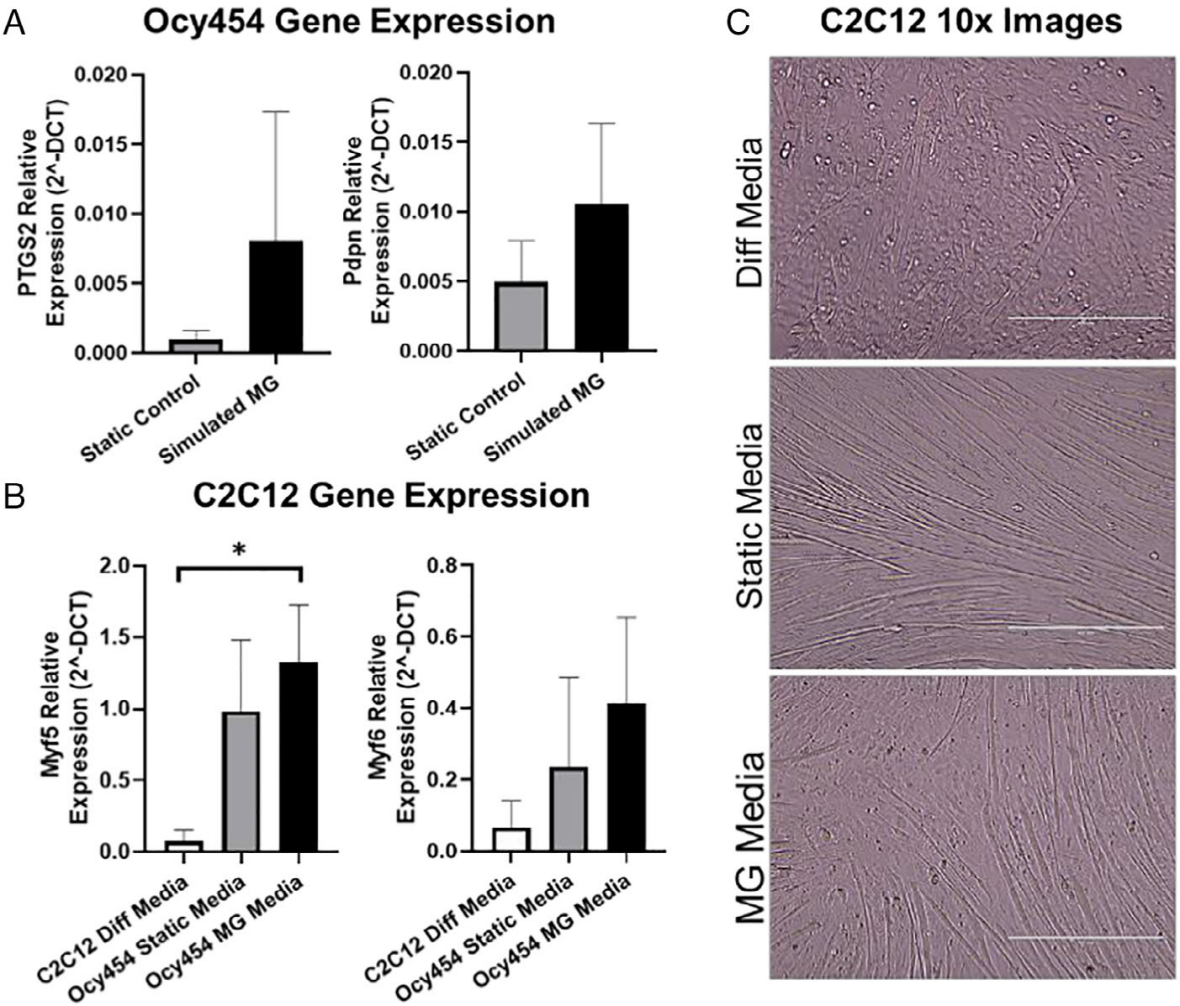
(A) Gene expression from Ocy454 cells undergoing either simulated microgravity or static ground conditions for 24 hours. Gene abbreviations: PTGS2 = prostaglandin‐endoperoxide synthase 2; Pdpn = podoplanin. (B) Gene expression from C2C12 cells cultured for 2 days in differentiation or Ocy454 conditioned media (static or microgravity [MG]). Gene abbreviations: Myf5 = myogenic factor 5; Myf6 = myogenic factor 6. *p < 0.05 by Tukey post hoc test. (C) representative C2C12 bright‐field images at day 2 of differentiation. Both Ocy454 conditioned static and MG media show increased myotube density versus C2C12 differentiation media.

Numerous osteocyte‐derived factors have been shown to influence muscle mass and function.^(1)^ Unloading of bone leads to bone loss via dysregulation of bone remodeling, a process primarily regulated by osteocytes.^(2,3)^ However, the molecular mechanisms regulating the osteocytic response to unloading and its downstream effects on muscle cells are unknown. We hypothesized osteocytes exposed to simulated microgravity (MG) secrete factors that alter muscle cell differentiation. Osteocytic Ocy454 cells (differentiated for 12 days at 37°C) were seeded on collagen 1 coated microcarrier beads and placed in a Rotary Cell Culture System. Disks were rotated at 15 to 17 rpm for 24 hours, simulating MG.^(4)^ Non‐rotating (static) disks were used as ground controls (*n* = 3/group). After 24 hours, mRNA was harvested from Ocy454 cells. Ocy454 conditioned media from disks (static or MG) or differentiation media (high glucose DMEM; 2% horse serum) was used to differentiate C2C12 myoblasts at 37°C (*n* = 3/media type). After 2 days of differentiation, images were taken, and mRNA was collected from C2C12 cells. After 24 hours of simulated MG, Ocy454 cells showed a nearly eightfold and twofold increase in Ptgs2 (encodes prostaglandin‐endoperoxide synthase 2) and Pdpn (encodes Podoplanin) gene expression, respectively, compared with static controls (Fig. 1
*A*). Culturing C2C12 myoblasts in MG conditioned media for 2 days significantly increased expression of Myf5 (encodes myogenic factor 5) versus C2C12 cells in differentiation media (Fig. 1
*B*). Myf6 (encodes myogenic factor 6) gene expression showed similar trends to Myf5 but was not significant. Images at day 2 reinforced these results by showing increased myotube density in C2C12 cells cultured in conditioned media (static and MG) versus differentiation media (Fig. 1
*C*). Furthermore, C2C12 Myf5 expression showed a significant and strong positive linear correlation to Ocy454 Ptgs2 expression (*r* = 0.99). These results support our hypothesis, suggesting osteocytes secrete factors that compensate for bone loss by promoting muscle differentiation. Furthermore, this effect is partially promoted by increased Ptgs2 expression, whose enzymatic end‐product prostaglandin has been previously shown to support C2C12 differentiation.^(6)^


## References


Bonewald L. Bone. 2019;120:212–8.Bikle DD, Halloran BP. J Bone Miner Metab. 1999;17(4):233–44.Bonewald LF. J Bone Miner Res. 2011;26(2):229–38.Spatz JM, et al. J Biol Chem. 2015;290(27):16744–58.Yaffe D, Saxel ORA. Nature. 1977;270(5639):725–7. Mo C, et al. Cell Cycle. 2015;14(10):1507–16.



**Whitney Bullock, PhD**


Indiana University, Indianapolis, IN, USA

Relevant Session: Biomechanical Relationships Between Muscle and Bone

DOI: 10.002/jbm4.10275


## Lrp4 Mediates Bone Mass and Mechanotransduction Through Interaction With Sclerostin In Vivo

### Whitney A Bullock, April Hoggatt, Daniel J Horan, Andrew Elmendorf, Amy Y Sato, Teresita Bellido, Gabriela G Loots, Fredrick M Pavalko, and Alexander G Robling

#### School of Medicine, Indiana University, Indianapolis, IN, USA

Wnt signaling plays a key role in regulating bone modeling and remodeling. in vitro studies suggest that sclerostin's inhibitory action on the Lrp5/6 Wnt co‐receptors is facilitated by the membrane‐associated receptor Lrp4. We explore this mechanism in vivo using an Lrp4 R1170W knockin mouse model (Lrp4KI), which was generated based on a published mutation in patients with high bone mass (HBM). Lrp4KI mice have an HBM phenotype (assessed by DXA, μCT, and pQCT), including increased bone strength and formation rates (assessed by mechanical testing and histomorphometry). At 18 weeks of age, Lrp4KI mice increased whole body BMD ~30%. Further analysis of the individual bone compartments in distal femurs showed increased trabecular BV/TV, trabecular number, and cortical thickness. Additionally, the high bone mass phenotype is observed in the lumber spine and skull. Surprisingly, testing of muscle function in vivo revealed compromised properties (reduced maximum torque) in Lrp4KI mice. Overexpression of a Sost transgene in bone tissue had osteopenic effects in Lrp4 WT but not Lrp4 KI mice. Conversely, inhibition of sclerostin had blunted osteoanabolic effects in Lrp4KI mice compared with WT mice. Four weeks of treatment with sclerostin antibody (Scl‐Ab) increased whole body BMD significantly compared with saline treatment in both WT and Lrp4KI female mice, but the antibody‐induced BMD gain exhibited by Lrp4KI mice was only about half of that exhibited by WT mice (263% in WT mice, 111% in Lrp4KI mice). Four‐week Scl‐Ab treatment increased bone formation parameters significantly in WT mice but not in Lrp4KI mice. In a model of disuse‐induced bone wasting, Lrp4KI mice exhibit significantly less bone loss than WT mice. The paralyzed limb of WT mice lost ~10% BMD over 4 weeks of muscle paralysis, but Lrp4KI mice did not lose BMD in response to Botox. In summary, mice harboring the Lrp4 R1170W missense mutation recapitulate the human HBM bone phenotype, are less sensitive to both increased and decreased sclerostin levels, and are protected from disuse‐induced bone loss. Lrp4 is an attractive target for pharmacological targeting aimed at increasing bone mass and preventing bone loss due to disuse or inactivity.


**Jarrod Call, PhD**


University of Georgia, Athens, GA, USA

DOI: 10.002/jbm4.10276


## Bone Deterioration and Metabolic Deficiency After Volumetric Muscle Loss Injury: Targets for Regenerative Rehabilitation

### Greising SM, Warren GL, and Call JA

#### 
^1^University of Minnesota; ^2^Georgia State University; ^3^University of Georgia

Volumetric muscle loss (VML) is characterized by a large volume of muscle tissue being removed due to surgery (eg, sarcoma) or severe trauma (eg, farm/industrial accident). There is currently no standard of clinical care to address the long‐term functional limitations of VML patients because the pathology and plasticity of the remaining muscle and bone are unknown. C57BL/6 mice underwent unilateral VML injury to the primary ankle plantarflexors and were subsequently used to investigate the underlying pathology and plasticity of the remaining tissue. A ~20% reduction in muscle volume resulted in a ~75% reduction in muscle strength that does not recover out to 4 months post‐VML. Bone function, ie, ultimate load, was 14% less in VML‐injured mice compared with uninjured controls and this corresponded with decrements in bone CSA (−14%) and CSMI (−20%). Rehabilitation strategies including wheel running and neuromuscular electrical stimulation were ineffective at correcting bone function and had minimal effect on muscle strength, leading us to hypothesize that the plasticity of the remaining tissue is compromised after VML. To test this hypothesis, we evaluated the metabolic plasticity of the remaining tissue to an endurance exercise training stimulus. Endurance training resulted in greater muscle oxidative capacity (ie, oxygen consumption) and mitochondrial quantity in uninjured mice, whereas VML‐injured mice were resilient to adaptation. We identified poor activation of the transcription factor PGC1α as a primary limitation to metabolic plasticity and showed that forced overexpression of PGC1α was sufficient to rescue metabolic plasticity. Using 2‐photon microscopy and the Dendra2 mitochondrial‐GFP‐labeled mice, we discovered widespread and long‐lasting irregularities in mitochondrial network organization in the remaining muscle linked to mitochondrial dysfunction. Thus, the remaining muscle is functionally limited and resistant to adaptation. Moving forward, we have analyzed RNA‐seq data sets from VML‐injured rats for muscle‐derived factors that may influence bone. Potential targets for future investigation include IL‐6 (3.5‐fold increase), IL‐7 (2.4‐fold increase), osteoglycin (10‐fold increase), and follistatin (15‐fold increase). By understanding the pathology and plasticity of the remaining muscle and the biomechanical and molecular influences it has on bone, we may be able to develop standards of care for VML patients in the future.


**Nabarun Chakraborty**


US Army Center for Environmental Health Research, Maryland City, MD, USA

DOI: 10.002/jbm4.10277


## Spaceflight Results in Muscle Loss and Is Linked to Energy Deprivation

### Nabarun Chakraborty,^1^ Aarti Gautam,^2^ David Waning,^3^ Paul Childress,^4^ Raina Kumar,^5^ George Dimitrov,^5^ Bintu Sowe,^6^ Rasha Hammamieh,^2^ and Melissa A. Kacena^4,7^


#### 
^1^Geneva Foundation, US Army Center for Environmental Health Research, Fort Detrick, MD, USA; ^2^Integrative Systems Biology, US Army Center for Environmental Health Research, Fort Detrick, MD, USA; ^3^Penn State College of Medicine, Hershey, PA, USA; ^4^Indiana University School of Medicine, Indianapolis, IN, USA; ^5^Advanced Biomedical Computing Center, NCI, Frederick, MD, USA; ^6^ORISE, US Army Center for Environmental Health Research, Fort Detrick, MD, USA; ^7^Richard L Roudebush VA Medical Center, Indianapolis, IN, USA

The unloading associated with spaceflight results in the rapid loss of muscle tissue thereby affecting functionality. This is among the most concerning physiologic change that occurs in space and could limit long‐term occupation in space. Thus, a better understanding of the mechanisms of changes to muscle mass, function, and metabolism could lead to development of improved therapies to counteract both spaceflight and terrestrial‐based muscle dysfunction. Here we used a non‐biased, stringent, deep sequencing (96 million paired end reads targeting 100 bp read length) assay to examine genomic networks altered by spaceflight in the quadriceps (*n* = 4/group). We also performed metabolomics analyses from serum (*n* = 4/group) to assess metabolite peaks. Nine‐week‐old C57BL/6 male mice were housed on the International Space Station or at Kennedy Space Center for approximately 4 weeks (*n* = 10/group). For genomics analyses, 14,228 genes (70% of whole mouse genome) met the cut‐off criteria, and the data sets were mapped to an average of ~76% of the whole mouse genome. Of these, 840 genes met the *t* test criteria, *p* < 0.05. For proteomic analyses, 740 metabolite peaks were significantly altered and met the *t* test criteria, *p* < 0.05. Analysis of the mRNA‐metabolite networks revealed inhibition of canonical networks linked to calcium ion homeostasis and muscle contraction in spaceflown mice. A comprehensive energy deprivation was indicated as functions related to protein synthesis and degradation, lipid synthesis and oxidation, and ATP hydrolysis were inhibited, and mitochondrial dysfunction was activated. This is the first time that skeletal muscle changes have been studied in male mice during spaceflight, and these mice were euthanized in space to avoid rehabituation to Earth's gravity. These data add important new findings to changes that occur in skeletal muscle in male mice during spaceflight.


**Phillip Dumesic, MD, PhD**



**Early Investigator Awardee*


Dana‐Farber Cancer Institute, Boston, MA, USA

DOI: 10.002/jbm4.10278


## An Evolutionarily Conserved uORF Regulates PGC1α and Oxidative Metabolism in Mice, Flies, and Bluefin Tuna

### Phillip A Dumesic,^1,2^ Daniel F Egan,^1,2^ Philipp Gut,^3^ Mei T Tran,^4,5^ Alice Parisi,^3^ Nirmalya Chatterjee,^6,7^ Mark Jedrychowski,^2^ Margherita Paschini,^8^ Lawrence Kazak,^9^ Sarah E Wilensky,^1^ Florence Dou,^1^ Dina Bogoslavski,^1^ Jeffrey A Cartier,^10^ Norbert Perrimon,^6,7^ Shingo Kajimura,^11^ Samir M Parikh,^4,5^ and Bruce M Spiegelman^1,2^


#### 
^1^Dana‐Farber Cancer Institute, Boston, MA, USA; ^2^Department of Cell Biology, Harvard University Medical School, Boston, MA, USA; ^3^Nestle Institute of Health Sciences, Lausanne, Switzerland; ^4^Division of Nephrology and Department of Medicine, Beth Israel Deaconess Medical Center and Harvard Medical School, Boston, MA, USA; ^5^Center for Vascular Biology Research, Beth Israel Deaconess Medical Center and Harvard Medical School, Boston, MA, USA; ^6^Department of Genetics, Harvard University Medical School, Boston, MA, USA; ^7^Howard Hughes Medical Institute; ^8^Boston Children's Hospital, Boston, MA, USA; ^9^Goodman Cancer Research Centre, Department of Biochemistry, McGill University, Montreal, Canada; ^10^Cartier and Company, LLC, Harwich, MA, USA; ^11^Diabetes Center and Department of Cell and Tissue Biology, University of California, San Francisco, CA, USA

Mitochondrial abundance and function are tightly controlled during metabolic adaptation and exercise but dysregulated in pathological states such as diabetes, neurodegeneration, cancer, and kidney disease. We show here that translation of PGC1α, a key governor of mitochondrial biogenesis and oxidative metabolism, is negatively regulated by an upstream open reading frame (uORF) in the 5′ untranslated region of its gene (PPARGC1A). We find that uORF‐mediated translational repression is a feature of PPARGC1A orthologs from human to fly. Strikingly, whereas multiple inhibitory uORFs are broadly present in fish PPARGC1A orthologs, they are completely absent in the Atlantic bluefin tuna, an animal with exceptionally high mitochondrial content and oxidative capacity. In mice, an engineered mutation disrupting the PPARGC1A uORF increases PGC1α protein levels and oxidative metabolism in multiple tissues and confers protection from acute kidney injury. These studies identify a translational regulatory element governing oxidative metabolism and highlight its potential contribution to the evolution of organismal mitochondrial function and exercise capacity.


**Karyn Esser, PhD**


University of Florida, Gainesville, FL, USA

DOI: 10.002/jbm4.10279


## Muscle Clocks, Exercise, and Bone‐Muscle Cross‐talk

### Lance A Riley, Denise Kemler, and Karyn A Esser

#### Department of Physiology and Functional Genomics, Myology Institute, University of Florida, Gainesville, FL, USA

The interaction of the muscle molecular clock, exercise, and bone‐muscle cross‐talk is a new and rapidly emerging area of research with significant implications for human health. Studies over the last 15 years have demonstrated the muscle molecular clock, in collaboration with MyoD1, plays a fundamental role in regulating a daily skeletal muscle transcriptional program. Disruption of this program leads to muscle weakness, altered metabolism, and age‐associated changes in musculoskeletal health. We have identified a subset of skeletal muscle‐secreted proteins, including Myostatin, TGFB1, and Irisin/FNDC5 that are downstream of the muscle clock with implications for skeletal tissue health. Exercise comes into this pathway as a time cue for the muscle clocks. This means that time of exercise will alter the phase of the daily transcriptional program in a healthy individual. This becomes very important as we show that time of exercise can serve to support muscle clock health and synchronization in conditions of chronic diseases when clocks are commonly disrupted. These new findings implicate time of exercise as a therapeutic intervention for supporting health musculoskeletal interactions.


**Alyson Essex, PhD**


Indiana University, Indianapolis, IN, USA

DOI: 10.002/jbm4.10280


## Lack of Osteocytic‐miR21 Promotes Skeletal Muscle Mass Growth in a Sex‐Specific Manner

### Alyson Essex,^1,4^ Hannah Davis,^1^ Padmini Deosthale,^1^ Andrea Bonetto,^1,3,4^ and Lilian Plotkin^1,2,4^


#### 
^1^Department of Anatomy and Cell Biology, Indiana University School of Medicine, Indianapolis, IN, USA; ^2^Roudebush Veterans Administration Medical Center, Indianapolis, IN, USA; ^3^Department of Surgery, Indiana University School of Medicine, Indianapolis, IN, USA; ^4^Indiana Center for Musculoskeletal Health, Indiana University, Indianapolis, IN, USA

Osteocytic microRNA21 (miR21) removal not only differentially alters cytokine production and bone mass, as well as osteoclast and osteoblast differentiation and activity in a sex‐dependent manner in mice, but also produces sex‐independent increases in mechanical bone strength. Because changes in bone remodeling and strength affect skeletal muscle through bone‐muscle cross‐talk, we aimed to investigate whether osteocytic miR21 deletion influences skeletal muscle. For this, we crossed miR21fl/fl mice with 8kbDMP1‐Cre mice to obtain OtmiR21Δ and miR21fl/fl control mice. Femora and tibias without bone marrow were obtained from female and male OtmiR21Δ and miR21fl/fl littermate control mice and cultured for 48 hours with 10% FBS/αMEM. Conditioned media (CM) was then collected to test the effects of bone‐derived factors on skeletal muscle cells. C2C12 cell differentiation was induced with 2% horse serum and the differentiated myotubes were exposed to 5% bone CM for 48 hours. CM from female OtmiR21Δ bones led to a 12% increase in average fiber size compared with CM from miR21fl/fl mice. Interestingly, CM generated from male bones did not change myotube diameter. Further, mRNA levels of IL6, a cytokine known to induce skeletal muscle atrophy, were 40% lower in bones from female OtmiR21Δ compared with control mice, whereas a Multiplex array showed that the levels of active phosphorylated‐Stat3 (p‐Stat3), a transcription factor activated by IL6, was 26% lower in the miR21‐deficient bones. Interestingly, no changes in IL6 or p‐Stat3 levels were found in male bones. Further, we found an increase in lean body mass (Dxa/Piximus) only in female OtmiR21Δ mice, even though muscle miR21 levels (qPCR) were similar in miR21fl/fl (0.05 ± 0.02) and OtmiR21Δ (0.09 ± 0.04) mice. To further study the role of osteocytic miR21 on skeletal muscle, we generated a new cohort of OtmiR21Δ mice. These mice exhibited increased soleus (42%) and gastrocnemius (21%) muscle weight only in females, whereas no changes were found in males. These data present a novel aspect of bone‐muscle cross‐talk in which osteocyte‐derived miR21 negatively influences skeletal muscle size in female but not male mice. Further studies are underway to elucidate the potential role of IL6 and the novel mechanism(s) responsible for miR21 effects on the bone‐muscle cross‐talk.


**Michael Friedman, PhD**



**Early Investigator Awardee*


Virginia Commonwealth University, Richmond, VA, USA

Relevant Session: Biomechanical Relationships Between Muscle and Bone

DOI: 10.002/jbm4.10281


## Differential Response to Unloading in Bones and Muscles of Diversity Outbred Mouse Founder Strains

### MA Friedman,^1^ CR Maroni,^1,2^ A Abood,^3^ Y Zhang,^1^ CR Farber,^3^ and HJ Donahue^1^


#### 
^1^Virginia Commonwealth University, Richmond, VA, USA; ^2^University “G. d'Annunzio” Chieti‐Pescara, Chieti, Italy; ^3^University of Virginia, Charlottesville, VA, USA

**Figure 2 jbm410257-fig-0002:**
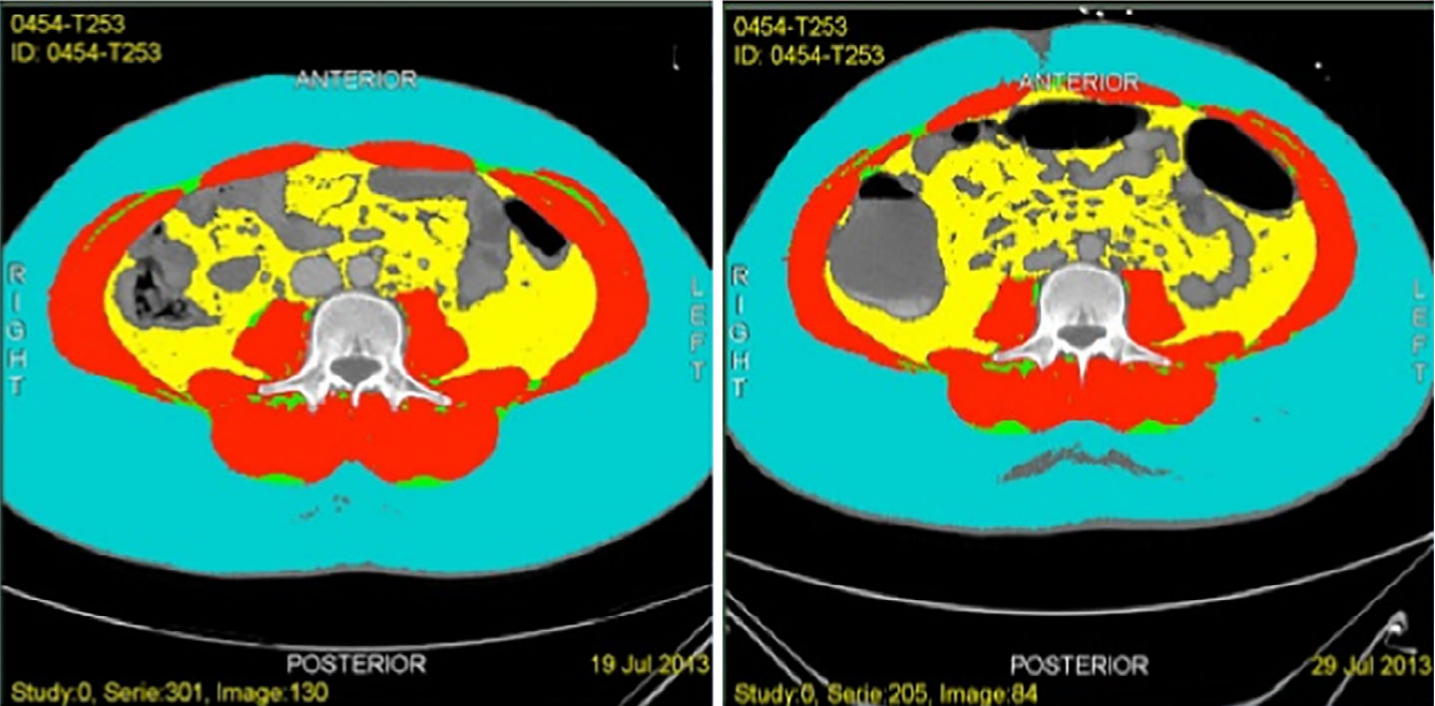
Visual loss of skeletal muscle on CT SMI

**Figure 3 jbm410257-fig-0003:**
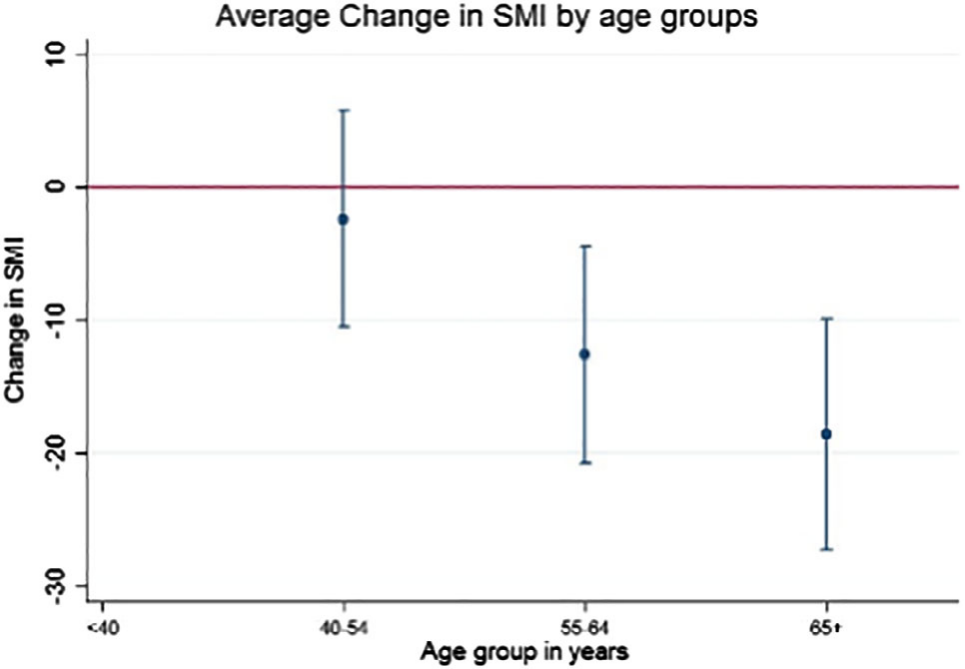
Visual loss of skeletal muscle on CT SMI

Mechanical unloading decreases bone and muscle volume up to 3% per month.^(1)^ Variations in lean mass and bone mass are influenced by genetics; however, it remains unclear how genetic variation affects their response to unloading. Diversity outbred mice (DO) are a diverse outbred population and are ideal for studying effects of genetic variability on the response to unloading.^(2)^ We examined phenotypic and transcriptomic responses to unloading in 8 DO founder strains (C57Bl/6J, A/J, 129S1/SvImJ, NOD/ShiLtJ, NZO/HlLtJ, CAST/EiJ, PWK/PhJ, and WSB/EiJ) after 3 weeks of single limb immobilization (SLI). We hypothesized there would be differential, strain‐dependent effects of unloading across these strains. Six 16‐week‐old male mice of each strain had the left limb immobilized in a cast for 3 weeks. The unaltered right limb was the control. Femoral bone geometry was analyzed by micro‐CT. Mechanical properties were tested by 3‐point bending. Quadriceps and gastrocnemius muscle samples were analyzed for markers of muscle atrophy and protein synthesis in 5 of the 8 strains. Two‐way RM ANOVAs with Tukey's tests were used to test for significant differences (*p* < 0.05). RNA‐seq was performed on tibial RNA from 7 strains. All strains had significantly different magnitudes of BV/TV loss (7% to 37%) in immobilized versus control limbs (Fig. 2). Quadriceps mass loss ranged from 4% to 45% and gastrocnemius mass loss from 1% to 33%. C57Bl/6J and CAST/EiJ had the greatest BV/TV loss, while NOD/ShiLtJ and NZO/HlLtJ had the greatest muscle loss. Only NOD/ShiLtJ and CAST/EiJ had upregulation of muscle atrophy genes. NOD/ShiLtJ had decreased protein synthesis. RNA‐seq revealed many significantly differentially expressed genes in immobilized tibias (Fig. 3). Immobilized bones displayed upregulation of osteogenic genes (col1a1, bglap, sparc), indicating a change in bone remodeling. This response differed across strains. The results indicate that different mouse strains respond to unloading from SLI differently. Interestingly, the two strains that lost the most bone to unloading were not the strains that lost the most muscle, suggesting a genetically based disconnect between bone and muscle loss in response to unloading. These results suggest DO mice will be a powerful model for examining effects of genetics on musculoskeletal response to unloading.

## References

1.Lang T, et al. J Bone Miner Res. 2004;19.

2.Svenson KL, et al. Genetics. 2012;190:2.


**Dorthée Girard, PhD**


Institut de Recherche Biomédicale des Armées, Clamart, France

DOI: 10.002/jbm4.10282


## Macrophage‐Derived Inflammation Promotes the Osteogenic Potential of Muscle Progenitor Cells and Contributes to Heterotopic Ossification in Trauma Patients

### Dorothée Girard,^1^* Frédéric Torossian,^2^* Bernadette Guerton,^2^ Adrienne Anginot,^2^ Marie‐Emmanuelle Goriot,^1^ Jules Gueguen,^1^ Denis Clay,^3^ Nathalie Rochet,^4^ Caroline Le Bousse‐Kerdilès,^2^ and Sébastien Banzet^1^


#### 
^1^Institut de Recherche Biomédicale des Armées, INSERM UMRS‐MD 1197, Clamart, France; ^2^INSERM UMRS‐MD 1197, Université Paris 11, Villejuif, France; ^3^INSERM UMS33, Université Paris 11, Villejuif, France; ^4^Université Côte d'Azur, CNRS, INSERM, Institut de Biologie Valrose, Nice, France

*Contributed equally to this work.

Heterotopic ossification (HO) is characterized by the development of ectopic bone tissue within periarticular muscle. HO is associated with severe pain, joint ankylosis, and vascular and nerve compression and the only curative option is surgical resection. Polytraumatic injuries and inflammation are the main identified factors contributing to the pathology, but the cellular mechanisms underlying the onset of HO still required further characterization. In this study, we investigated the role of macrophage‐derived inflammation via the secretion of oncostatin M (OSM) in modulating the osteogenic potential of muscle stem cells. Human HO samples were collected during surgery to isolate CD14+ macrophages. Samples of muscle surrounding HOs were also collected to isolate CD56+ myoblasts and PDGFRα+ mesenchymal progenitors using cell sorting. Conditioned medium of CD14+ cells stimulated or not with LPS and OSM was added during CD56+ and PDGFRα+ in vitro osteogenic differentiation assays and calcium deposition was quantified using Alizarin Red S staining. CD56+ and PDGFRα+ cells were also seeded into hydroxyapatite/calcium phosphate scaffolds and implanted subcutaneously into the flanks of nude mice for in vivo osteogenic assays. After 15 weeks, scaffolds were decalcified for H&E histological evaluation.

Our results show that conditioned medium of macrophages stimulated with LPS and the addition of OSM increase calcium deposition for both CD56+ and PDGFRα+ cells. Interestingly, PDGFRα+ cells display higher in vitro osteogenic differentiation potential compared with CD56+ cells. The histological analyses of scaffolds seeded with CD56+ cells showed collagen matrix deposition, whereas PDGFRα+ cells were able to generate mature bone matrix. More interestingly, the presence of hematopoietic stem cells was mainly observed in PDGFRα+ seeded scaffolds. This study confirms that macrophage‐derived inflammation and more particularly OSM is a major component of HO pathophysiology. PDGFRα+ precursor cells display higher osteogenic potential and can mediate de novo formation of a hematopoietic stem cell niche.


**Theresa Guise, MD, PhD**


Indiana University, Indianapolis, IN.

DOI: 10.002/jbm4.10283


## Bone‐Derived TGF‐β Impairs Glucose Metabolism and Insulin Release by Oxidation of RyR2 Ca2+ Release Channel in Pancreatic β‐Cells in the Setting of High Bone Turnover, Aging, and High‐Fat Diet

### Trupti Trivedi,^1^ Jenna N Regan,^1^ Asma Bahrami,^1^ Jennymar Rojas,^1^ Steven Reiken,^2^ Sutha John,^1^ Sreemala Murthy,^1^ Yun She,^1^ Gabriel M Pagnotti,^1^ Sarah A Tersey,^3^ Xu Cao,^4^ Andrew R Marks,^2^ Carmella Evans‐Molina,^1,3^ Khalid S Mohammad,^1^ and Theresa A Guise^1^


#### 
^1^Division of Endocrinology, Department of Medicine, Indiana University School of Medicine, Indianapolis, IN, USA; ^2^Department of Physiology and Cellular Biophysics, Helen and Clyde Wu Center for Molecular Cardiology; ^3^College of Physicians and Surgeons, Columbia University, New York, NY, USA; ^4^Department of Pediatrics, Indiana University School of Medicine, Indianapolis, IN, USA; ^5^Department of Orthopedic Surgery, Johns Hopkins University School of Medicine, Baltimore, MD, USA

Bone destruction in cancer or other pathology causes fractures, pain, and muscle weakness. TGF‐β released from bone via osteoclastic bone resorption acts systemically to cause skeletal muscle weakness through oxidation of sarcoplasmic reticulum Ca + 2 channel, ryanodine receptor (RyR). Since oxidation of pancreatic‐b‐cell RyR2 can impair insulin secretion and bone destruction results in systemic TGF‐β effects, we hypothesized that states of increased bone resorption with release of TGF‐β causes oxidation of pancreatic‐β‐cell RyR2 to impair insulin secretion and glucose homeostasis. We studied the effects of bone‐derived TGF‐b on the pancreas using a model of Camurati‐Engelmann disease (CED), a bone dysplasia with increased TGF‐b and bone turnover. CED mice had increased circulating TGF‐β, reduced serum insulin, and increased pSmad2/3 in pancreatic‐b‐cells. Forty‐five‐week‐old CED mice fed a high‐fat diet (HFD) for 15 weeks developed glucose intolerance (*p* < 0.01) and impaired insulin (*p* < 0.01) secretion (glucose‐stimulated insulin secretion in isolated islets) versus HFD‐WT mice. Both HFD‐CED and HFD‐WT had insulin resistance (via ITT) compared with CED and WT fed low‐fat diet (LFD). HFD‐CED mice had higher fat mass (*p* < 0.001), skeletal muscle weakness (*p* < 0.001), and reduced muscle‐fiber diameter (*p* < 0.001) compared with HFD‐WT. HFD‐CED mice had reduced bone mineral density (*p* < 0.001) and increased cortical porosity (*p* < 0.01) compared with HFD‐WT mice. Impaired insulin secretion and skeletal muscle weakness in HFD‐CED mice were associated with Nox4‐mediated oxidation of pancreatic β‐cell RyR2 and skeletal muscle RyR1, respectively. TGF‐β had direct effects on insulin secretion as isolated pancreatic islets from WT mice treated with TGF‐β showed increased phosphoSmad3 and Nox4‐mediated oxidation of RyR2. Further, TGF‐β decreased expression of pro‐insulin (*ins‐1* and *ins‐2*) mRNA. Collectively, these data suggest that states of increased bone destruction can disrupt glucose metabolism, pancreatic β cell insulin secretion, and causes muscle weakness, via systemic effects of bone‐derived TGF‐β to oxidize RyR. These effects, exacerbated by HFD and aging, have implications for bone health as impaired glucose metabolism and muscle weakness can further increase fracture risk. Blocking bone destruction, the release of TGF‐β, and preventing RyR Ca2+ leak in pathologic bone destruction should reduce fracture risk by improving hyperglycemia, muscle weakness, and subsequent bone quality.


**Mark Hamrick, PhD**


Medical College of Georgia, Augusta, GA, USA

DOI: 10.002/jbm4.10284


## The Kynurenine Pathway in Musculoskeletal Aging

### Mark W Hamrick,^1^ Carlos M Isales,^1^ Helen Kaiser,^1^ Beom‐Jun Kim,^2^ Meghan McGee‐Lawrence,^1^ Sadanand Fulzele,^1^ and William D Hill^3^


#### 
^1^Medical College of Georgia, Augusta University, Augusta, GA, USA; ^2^Asan Medical Center, University of Ulsan College of Medicine, Seoul, South Korea; ^3^Medical University of South Carolina, Charleston, SC, USA

Kynurenine (KYN) is a circulating tryptophan (TRP) metabolite that increases with age and is implicated in several age‐related disorders. KYN is metabolized from tryptophan by two major enzymes: tryptophan 2, 3 dioxygenase (TDO) in the liver, and indoleamine 2, 3 dioxygenase (IDO) extrahepatically. An increase in IDO activity has been linked to an increased mortality rate in humans, and frailty is associated with a marked increase in the KYN/TRP ratio. We have recently studied the effects of kynurenine on bone and muscle in both humans and animal models. Serum kynurenine levels increase significantly with age in mice, consistent with data previously reported for human subjects. Levels of serum markers of osteoclastic activity (pyridinoline [PYD] and RANKL) increase significantly with KYN treatment in adult (12‐month mice). The increase in osteoclast activity with KYN treatment is associated with a significant decrease in vertebral bone volume (BV/TV). We also examined kynurenine levels in patients with and without hip fracture (HF). Patients undergoing hip replacement for fragility HF (*n* = 27) had a ~40% higher bone marrow kynurenine level than patients undergoing hip replacement for other reasons (*n* = 45). In addition, bone marrow KYN level was inversely associated with total femur BMD even after adjustment for sex, age, and body mass index. We have also investigated the effects of KYN on skeletal muscle and found that KYN treatment in young mice produces an aged phenotype characterized by muscle fiber atrophy. Results from these studies suggest that targeting the kynurenine pathway with aging may be a potential therapeutic strategy for improving muscle and bone health. Funding for this research was provided by the National Institute on Aging (AG 036675).


**Jennifer Hartwell, MD, FACS, CNSC**


Indiana University, Indianapolis, IN, USA

DOI: 10.002/jbm4.10285


## Trauma Patients Demonstrate a Rapid Decline in Skeletal Muscle Index Despite Early Adequate Nutrition

### Mozhu Li, Lava Timsina, Chelsea Wenos, Ann Cotton, Teresa Zimmers, and Jennifer L Hartwell

#### Indiana University, Indianapolis, IN, USA

Sarcopenia in older patients at admission and low psoas muscle density in younger patients at admission both associate with poor outcomes after trauma. Less is known about in‐hospital loss of muscle mass in trauma patients of all ages. We hypothesized that adequate nutrition would help mitigate the loss of skeletal muscle mass during hospitalization. We identified 64 critically injured trauma patients with admission CT and second CT within 30 days. We measured skeletal muscle area from these CT scans using Sliceomatic software. Skeletal muscle index (SMI) was calculated from muscle area normalized to height for each time point and the difference determined. Patients were placed into three groups: SMI decline of <10%, 10% to 20%, or >20% from baseline. Relationships among patient age, sex, injury severity score, injury type, nutrition delivery, and morbidity were determined using univariate and multivariate analysis. Patient characteristics: 76.6% male, 92.2% with blunt injury, mean age of 47.16 ± 18.2 years. Of these, 42.2% had a <10% drop in SMI, 31.3% had a 10% to 20% drop, and 12.5% had a >20% SMI drop. Total protein deficiency was not significantly different among groups (*p* = 0.093). On multivariable analysis, age (*p* < 0.001), female sex (*p* = 0.004), APACHE II score (*p* = 0.042), and feeding intolerance (*p* = 0.004) were all significantly associated with change in SMI; ISS, injury mechanism, complications, and total protein deficit were not. By random effects modeling, age over 55 years was significantly associated with decrease in SMI. Despite adequate protein delivery, almost half of these seriously injured patients lost >10% and 12.5% lost >20% skeletal muscle mass within 30 days of injury. Such loss of muscle mass despite adequate nutrition, also known as cachexia, is associated with morbidity and mortality across diseases. The striking muscle loss documented here could contribute to longer‐term disability and mortality after trauma. Methods to mitigate trauma‐induced cachexia should be investigated.


**Namki Hong, MD**


Yonsei University College of Medicine

Relevant Session: Muscle‐Bone Interactions During Aging

DOI: 10.002/jbm4.10286


## Computed Tomography (CT)‐Derived Skeletal Muscle Radiodensity Is a More Sensitive Marker Than Skeletal Muscle Area for the Age‐Related Musculoskeletal Changes in Healthy Adults

### Namki Hong, Junchae Na, Woong Kyu Han, Yumie Rhee

#### 
^1^Department of Internal Medicine, Severance Hospital, Endocrine Research Institute; ^2^Yonsei University College of Medicine; Department of Urology, Severance Hospital, Seoul, Republic of Korea

Assessment of sarcopenia by computed tomography (CT)‐derived muscle parameters is recently endorsed by European sarcopenia guideline. Although opportunistic diagnosis of sarcopenia in routine clinical CT can provide useful information, there are limited data regarding cut‐off value for sarcopenia based on CT and age‐related changes in muscle and bone parameters in healthy Korean adults. From kidney donor registry of a tertiary institution between 2006 to 2014, preoperative unenhanced abdominal CT scans of kidney donors (*n* = 593; aged 19 to 69 years) were analyzed. Sarcopenia cut‐off was determined by EWGSOP2 guideline (2 standard deviations below the mean of young reference group aged 19 to 39 years; *n* = 299) for skeletal muscle area (SMA, cm^2^), skeletal muscle index (SMI, SMA/height^2^, cm^2^/m^2^), and skeletal muscle radiodensity (SMD, HU) at lumbar area (L_1_ to L_5_). SMA and SMI at L_3_ showed highest value among all lumbar areas. Diagnostic threshold for sarcopenia for SMA, SMI, and SMD at L_3_ in community‐dwelling Korean adults were as follows: 132.9 cm^2^ and 82.5 cm^2^; 43.9 cm^2^/m^2^ and 33.7 cm^2^/m^2^; and 37.3 HU and 33.7 HU for men and women, respectively. SMA and SMD at L_3_ showed strong correlation with L_2_ (*r* = 0.97 and 0.94) and L_4_ (*r* = 0.93 and 0.96; *p* < 0.001 for all), followed by L_1_ and L_5_. Per 5‐year increase of age, L_3_ SMI showed linear decrease in men (−0.75 cm^2^/m^2^; *p* = 0.004) but not in women (0.03 cm^2^/m^2^; *p* = 0.826). However, L_3_ SMD showed significant linear decrease in both men and women (−1.15 HU and − 1.31 HU per 5‐year increase in men and women; *p* < 0.001 for all). Compared with L_3_ SMA, L_3_ SMD showed better discriminatory performance for detecting low L_1_ trabecular bone attenuation (<110 HU) (area under the receiver operating characteristics curve [AUROC] 0.855 versus 0.686; *p* < 0.001). Cut‐off value for CT‐derived L_3_ SMI and L_3_ SMD in Korean population was presented. L_3_ SMD might be a more sensitive marker than L_3_ SMA for aging‐related changes of bone and muscle.


**Joshua Huot, PhD**


Indiana University, Indianapolis, IN, USA

DOI: 10.002/jbm4.10287


## Metastatic Colorectal Cancer Induces Musculoskeletal and Metabolic Abnormalities

### Joshua R Huot,^1,2^ Leah J Novinger,^3^ Fabrizio Pin,^2,4^ Alyson L Essex,^4^ Alexander J Jones,^3^ Thomas M O'Connell,^2,3,5,6^ and Andrea Bonetto^1,2,3,4,5,6^


#### 
^1^Department of Surgery, Indiana University School of Medicine, Indianapolis, IN, USA; ^2^IUPUI Center for Cachexia Research, Innovation and Therapy, Indiana University School of Medicine; ^3^Department of Otolaryngology–Head & Neck Surgery, Indiana University School of Medicine; ^4^Department of Anatomy and Cell Biology, Indiana University School of Medicine; ^5^Indiana Center for Musculoskeletal Heath, Indiana University School of Medicine; ^6^Simon Cancer Center, Indiana University School of Medicine

Colorectal cancer (CRC) is a leading cause of death worldwide and in the most advanced state is often accompanied by the development of liver metastases and skeletal muscle wasting, ie, cachexia. Despite affecting the majority of CRC patients, cachexia remains understudied and currently has no cure. A limited number of elementary characterized animal models for CRC are available, and only a single model of liver metastases associated with CRC has been developed for the study of cachexia. We aimed to further characterize this model by focusing on functional, molecular, and metabolic effects on muscle. CD2F1 male mice were intrasplenically injected with C26 tumor cells (mC26) to mimic hepatic dissemination of cancer cells, while sham‐operated animals received saline (*n* = 5/group). Animals were assessed weekly for body weight and grip strength. Upon euthanization, tissues (muscles, liver, and bone) were collected for morphological and molecular analyses. Liver metastatization of C26 cells was associated with progressive and significant loss of body weight (−13%). Consistently, mC26 bearers displayed significant reductions in muscle weights (gastrocnemius: –26%; quadriceps: −33%), supported by decreased muscle strength (−23%) and cross‐sectional area (−22%). MicroCT analysis revealed that loss of skeletal muscle in mC26 hosts was accompanied by reductions in bone mass as indicated by reductions in trabecular bone volume fraction (BV/TV: −45%) and trabecular thickness (Tb.Th: −11%). At the molecular level, skeletal muscle of mC26 mice showed reduced phosphorylation of the markers of protein anabolism mTOR, 4EBP1, and p70S6K, along with increased levels of phospho‐STAT3, ubiquitin, MuRF‐1, and Atrogin‐1, also suggesting enhanced protein catabolism. mC26 hosts also showed prevalence of fibers with glycolytic metabolism and enhanced lipid accumulation, in line with mitochondrial abnormalities, as also evidenced by reduced levels of PGC1α and Mitofusin 2 and reduced enzymatic activity of succinate and pyruvate dehydrogenase. Metabolomics analysis by NMR revealed systemic reductions in glucose and reduced branched‐chain amino acid levels, suggesting abnormalities in energy metabolism. Overall, our model recapitulates the cachectic phenotype of metastatic CRC and displays loss of muscle and bone mass, accompanied by reduced muscle anabolism, increased protein catabolism, abnormal mitochondrial homeostasis, and metabolic deficits.


**Abdurahman Jama, PhD (c)**


Wright State University, Dayton, OH, USA

Relevant Session: Role of Muscle and Bone Factors in Energetics and Metabolism

DOI: 10.002/jbm4.10288


## Lipin‐1 Is Required for Skeletal Muscle Development by Regulating MEF2c and MyoD Expression

### Abdurahman Jama,^1^ Dengtong Huang,^1^ Abdullah A Alshudukhi,^1^ Roman Chrast,^2^ and Hongmei Ren^1^


#### 
^1^Department of Biochemistry and Molecular Biology, Wright State University, Dayton, OH, USA; ^2^Department of Neuroscience and Department of Clinical Neuroscience, Karolinska Institute, Stockholm, Sweden

Our previous characterization of global lipin1‐deficient (fld) mice demonstrated that lipin1 played a novel role in skeletal muscle (SM) regeneration. The present study using cell type‐specific Myf5‐cre;Lipin1fl/fl conditional knockout mice (Lipin1Myf5cKO) shows that lipin1 is a major determinant of SM development. Lipin1 deficiency induced reduced muscle mass and myopathy. Our results from lipin1‐deficient myoblasts suggested that lipin1 regulates myoblast differentiation via the protein kinase Cμ (PKCμ)/histone deacetylase 5 (HDAC5)/myocyte‐specific enhancer factor 2C (MEF2c):MyoD‐mediated pathway. Lipin1 deficiency leads to the suppression of PKC isoform activities, as well as inhibition of the downstream target of PKCμ, class II deacetylase HDAC5 nuclear export, and, consequently, inhibition of MEF2c and MyoD expression in the SM of lipin1Myf5cKOmice. Restoration of diacylglycerol‐mediated signaling in lipin1‐deficient myoblasts by phorbol 12‐myristate 13‐acetate transiently activated PKC and HDAC5, and upregulated MEF2c expression. Our findings provide insights into the signaling circuitry that regulates SM development and have important implications for developing intervention aimed at treating muscular dystrophy.


**Daenique Jengelley, MS (c)**


Indiana University, Indianapolis, IN, USA

Relevant Session: Muscle‐Bone Interactions in Cancer

DOI: 10.002/jbm4.10289


## Musculoskeletal Effects of Oncostatin M

### Daenique Jengelley,^1^ Meijing Wang,^2^ Andrea Bonetto,^3^ Alexander Robling,^2^ and Teresa A Zimmers^1,2,3,4,5,6^


#### Departments of ^1^Biochemistry and Molecular Cell Biology, ^2^Anatomy and Cell Biology, ^3^Surgery, ^4^IU Simon Cancer Center, ^5^IUPUI Center for Cachexia Research, Innovation, and Therapy, and ^6^Indiana Center for Musculoskeletal Health, Indiana University School of Medicine, Indianapolis, IN, USA

Cachexia is a chronic muscle‐ and fat‐wasting syndrome combined with significant body weight loss. It is a comorbidity associated with most cancers and affects more than 85% of patients with pancreatic cancer. The tumor secretes cytokines that induce a systemic inflammatory response leading to cachexia. The JAK/STAT pathway is commonly activated in pancreatic cancer‐cachexia by the Interleukin‐6 family of cytokines. The most studied of these cytokines is Interleukin‐6 (IL‐6); however, less is known of the other IL‐6 family of cytokines and their roles in the development of pancreatic cancer‐cachexia. Here, we investigate the musculoskeletal effects of Oncostatin M (OSM) in a noncancerous inflammatory condition. OSM was first characterized to inhibit tumor cell proliferation but now has identified roles in inflammatory disorders, cell proliferation, fibrosis, and cytokine secretion. OSM expression is localized to the blood, and it is secreted by immune cells; however, the receptor is more widely expressed (GTEx). There is little to no mRNA expression of OSM in muscle of mice; however, the receptor is more highly expressed in muscle (muscleDB). In bone, OSM induces expression of RANKL, osteoclast formation, and functions in hematopoiesis and thrombocytosis. In fat, OSM functions as an adipokine. In cardiac muscle, OSM promotes differentiation of cardiomyocytes and exacerbates cardiac failure. In skeletal muscle, OSM inhibits muscle stem cell differentiation. Less is known of the mechanisms of OSM in pancreatic cancer‐cachexia. In vitro, we have treated C2C12 myotubes with recombinant OSM for 48 hours and observed myotube wasting. Using an adeno‐associated virus expressing Osm, we observed skeletal muscle and fat wasting in wild‐type and IL‐6 knockout mice. Echocardiography results showed reduced ejection fraction (%) and fractional shortening (%) in AAV‐Osm groups compared with AAV‐Null over 12 weeks. These effects were independent of IL‐6. The results in bone are pending. These preliminary data along with publicly available data and relevant literature support a role for OSM in skeletal muscle and bone and future studies in pancreatic cancer‐cachexia.


**Zhihao Jia, PhD**


Purdue University, West Lafayette, IN, USA

Relevant Session: Muscle‐Bone Interactions in Cancer

DOI: 10.002/jbm4.10290


## Polo‐Like Kinase 1 Is Essential for Cell Cycle Progression and Survival of Skeletal Myoblasts

### Zhihao Jia,^1^ Yaohui Nie,^1,2^ Feng Yue,^1^ Yifan Kong,^1^ Lijie Gu,^1^ Timothy Gavin,^2^ Xiaoqi Liu,^3,4^ and Shihuan Kuang^1,4^


#### 
^1^Department of Animal Sciences, ^2^Department of Health and Kinesiology, ^3^Department of Biochemistry, and ^4^Center for Cancer Research, Purdue University, West Lafayette, IN, USA

Muscle development and regeneration require delicate cell cycle regulation of embryonic myoblasts and adult muscle satellite cells (MuSCs). Through analysis of the Polo‐like kinase (Plk) family cell‐cycle regulators in mice, we show that Plk1's expression closely mirrors myoblast dynamics during embryonic and postnatal myogenesis. Cell‐specific deletion of *Plk1* in embryonic myoblasts leads to depletion of myoblasts, developmental failure, and prenatal lethality. Postnatal deletion of *Plk1* in MuSCs does not perturb their quiescence but depletes activated MuSCs as they enter the cell cycle, leading to regenerative failure. The *Plk1*‐null MuSCs are arrested at the M‐phase, accumulate DNA damage, and apoptose. Mechanistically, *Plk1* deletion upregulates p53, and inhibition of p53 promotes survival of the *Plk1*‐null myoblasts. Pharmacological inhibition of Plk1 similarly inhibits proliferation but promotes differentiation of myoblasts in vitro and blocks muscle regeneration in vivo. These results reveal for the first time an indispensable role of Plk1 in developmental and regenerative myogenesis.


**Camilo Morales Jimenez, PhD**



**Early Investigator Awardee*


Pontifica Universidad, Javeriana Cali, Columbia

Relevant Session: Role of Soluble Factors in Muscle‐Bone Interactions

DOI: 10.002/jbm4.10291


## Osteoclasts Release ATP to the Extracellular Medium by Mechanical Stimuli and Increase Protein Synthesis in Skeletal Muscle Through the Activation of P2Y Receptors Associated With the PI3K‐Akt–mTOR Pathway

### Camilo Morales,^1,2,3^ Manuel Arias‐Calderón,^1,2^ Nadia Hernández,^1^ Enrique Jaimovich,^2^ and Sonja Buvinic^1^


#### 
^1^Molecular Cell Biology Laboratory, Institute for Research in Dental Sciences, Faculty of Dentistry, Universidad de Chile; ^2^Center for Molecular Studies of the Cell, ICBM, Faculty of Medicine, Universidad de Chile; ^3^Department of Health Sciences, Faculty of Health, Pontificia Universidad, Javeriana‐Cali‐Colombia

In this research, we demonstrated that osteoclasts, purified from the RAW264.7 cell line differentiated by RANKL, release ATP to the extracellular medium both basally and in response to mechanical stimulation by medium perturbation. The ATP release is proportional to the intensity of the stimulus and independent of cell lysis. The basal release of ATP occurs both via vesicular exocytosis and via conductive mechanisms through pannexin 1 hemichannels. The ATP release evoked by mechanical stimulation occurs via a conductive mechanism mediated by P2X7 receptors. In parallel, we demonstrated that exogenous ATP promotes Akt phosphorylation (S473) in FDB muscle isolated from adult mice, in a time‐ and concentration‐dependent manner, with maximal values at 7 to 15 minutes and 3 μM. ATP also induced phosphorylation of proteins downstream Akt: mTOR (S2448), p70S6K (T389), and 4E‐BP1 (T37 / 46). ATP 3 μM increased the protein synthesis rate in FDB muscle by 2.2 times; this effect was blocked with suramin (general P2X / P2Y antagonist), LY294002 (phosphatidylinositol 3 kinase inhibitor), and rapamycin (mTOR inhibitor). ATP 3 μM did not significantly modify the activity of the degradation pathway that involves ubiquitin 3 ligase‐proteosome. Finally, using co‐cultures in Transwell chambers, we demonstrated that mechanically stimulated osteoclasts promote protein synthesis in isolated FDB muscle, through a mechanism dependent on the ATP release and activation of the P2‐PI3K‐Akt–mTOR pathway in muscle cells.


**Stephanie Y Jo, MD, PhD**



**Early Investigator Awardee*


University of Philadelphia, Philadelphia, PA, USA

DOI: 10.002/jbm4.10292


## Interaction of Cartilage and Subchondral Bone in H3K79 Methyltransferase DOT1L Loss Mouse Model

### Stephanie Y Jo,^1^ Miriam S Domowicz,^2^ Judith G Henry,^2^ and Nancy B Schwartz^2^


#### 
^1^University of Pennsylvania; ^2^University of Chicago

Osteoarthritis and osteoporosis are widely prevalent and have far‐reaching public health implications. There is increasing evidence that epigenetics, in particular histone 3 lysine 79 methyltransferase DOT1L, plays an important role in the cartilage and bone biology. In this study, we evaluate the role of Dot1l in cartilage, growth plate, and subchondral bone utilizing conditional knockout mouse models. We generated chondrocyte‐specific constitutive and inducible conditional Dot1l knockout mouse lines using Col2a1‐Cre and Acan‐CreER systems. Techniques including whole‐mount alcian blue stain, in situ hybridization, microCT, immunohistochemistry, and quantitative PCR were used for analyzing the mouse model. Prenatal deletion of Dot1l in mouse chondrocytes led to perinatal mortality, accelerated ossification, and dysregulation of Col10a1 expression. Postnatal deletion of Dot1l in mouse chondrocytes resulted in subchondral trabecular weakening, decreased extracellular matrix production, and disruption of the growth plate. In addition, pharmacological inhibition of DOT1L in a progeria mouse model partially rescued the abnormal osseous phenotype. In conclusion, Dot1l is important in the maintenance of growth plate, extracellular matrix production, and subchondral bone.


**Mark Johnson, PhD**


University of Missouri – Kansas City, School of Dentistry, Kansas City, MO, USA

DOI: 10.002/jbm4.10293


## The Role of Estrogen in Muscle‐Bone Cross‐talk

### Nuria Lara‐Castillo, Erica Jackson, Kika Masunaga, Mark Dallas, Mark Gray, Julian Vallejo, Michael Wacker, and Mark L Johnson

#### University of Missouri – Kansas City, Kansas City, MO, USA

Osteoporosis (bone loss, increased fracture risk) and sarcopenia (decreased muscle mass and function) represent a significant health care burden. In females, both diseases manifest in the peri/postmenopausal period of life, which is characterized by a decline in female sex hormone estrogen. We hypothesize that loss of estrogen‐mediated signaling with aging impairs cross‐talk signaling between bone and muscle. We are investigating the mechanisms by which muscle and bone biochemically communicate with each other, the effects of aging on these mechanisms, and the role of estrogen receptor‐mediated signaling in regulating the production of factors involved in this biochemical cross‐talk. An important pathway involved in both muscle development/function and bone (osteocyte) response to mechanical loading is the Wnt/β‐catenin signaling pathway. Addition of conditioned media (CM) from C2C12 muscle myotubes (but not myoblasts) enhances β‐catenin signaling in TOPflash‐MLO‐Y4 osteocytes 2‐fold (*p* < 0.05). Wnt treatment (10 ng/mL for 24 hours) of TOPflash‐MLO‐Y4 cells activates β‐catenin signaling 10‐fold (*p* < 0.05). CM plus Wnt synergistically activates β‐catenin signaling by ~30‐fold (*p* < 0.05). Treatment of C2C12 myotubes with the mTOR pathway inhibitor, KU‐0063794, reversibly inhibits the production of this factor. Initial attempts to identify this factor suggest it is a water‐soluble molecule(s) of molecular of MW >10Kd. in vivo mechanical loading activates β‐catenin signaling in osteocytes (~2.5‐fold, *p* < 0.05), which is blocked by either ovariectomy or BOTOX‐induced paralysis of adjacent muscles. Treatment of TOPflash‐MLO‐Y4 cells with the estrogen receptor inhibitor ICI 182,780 blocks fluid flow shear stress activation of β‐catenin signaling. Preliminary studies on mice with muscle‐targeted deletion (HSA‐MCM‐Cre) of the estrogen receptor‐β isoform, ERβ, did not alter the load‐strain relationship in female tibia but in male tibia resulted in a left shift. Dmp1‐Cre‐targeted deletion of ERβ in male mice resulted in a significant reduction in femur trabecular BMD, BV/TV, number, and increased separation. Heart weight/body weight was not altered, and no differences in EKG intervals were observed in this preliminary study of Dmp1‐Cre‐deleted ERβ mice. Muscle function is currently being analyzed in these models. These data demonstrate the importance of estrogen and ERs in bone and muscle and cross‐talk between these two tissues.


**Evangelia Kalaitzoglou, MD**


University of Kentucky, Lexington, KY, USA

Relevant Session: Role of Muscle and Bone Factors in Energetics and Metabolism

DOI: 10.002/jbm4.10294


## The Effects of Myostatin in Pre‐Osteoblast Cells in Normoglycemic and Hyperglycemic Conditions

### Evangelia Kalaitzoglou,^1,2^ Callie Knuckles,^3^ and R Clay Bunn^1,2^


#### 
^1^Pediatrics, University of Kentucky, Lexington, KY, USA; ^2^Barnstable Brown Diabetes Center, University of Kentucky, Lexington, KY, USA; ^3^College of Agriculture, Food, and Environment, University of Kentucky, Lexington, KY, USA

Myostatin, or Growth and Differentiation Factor 8 (GDF‐8), a member of the TGF‐β family primarily expressed in skeletal muscle, is a negative regulator of both muscle and bone mass. Myostatin has been shown to have a negative effect on bone marrow–derived stromal cells and bone healing. Furthermore, myostatin levels are high in animal models of insulin‐deficiency (models of diabetes mellitus) that are proven to have low bone mass and impaired bone quality. However, its effects on bone cells, particularly in a hyperglycemic environment, are not well known. This study aims to assess the impact of myostatin on genes regulating osteoblast differentiation in osteoblasts under normal and hyperglycemic conditions. Myostatin and AcvR2b (myostatin receptor) transcripts were quantified in MC3T3‐E1 cells (pre‐osteoblast murine cell line). Additionally, cells were stimulated with vehicle or myostatin, and collected and processed via Western blot to assess activation of myostatin‐related signaling pathways such as Smad2/3. Quantification of runt related transcription factor 2 (RUNX2) and Osterix (Osx) after myostatin stimulation for 24 hours was evaluated under normo (5.5 mM)‐ and hyperglycemic (15 mM) conditions using qRT‐PCR. We have demonstrated that although MC3T3‐E1 cells lack myostatin mRNA, transcripts for the myostatin receptor (Acv2b) were detected, indicating their potential to respond to myostatin. We have confirmed myostatin intracellular signaling in these cells, as exogenous myostatin resulted in Smad2 phosphorylation. Experiments looking at the impact of myostatin on the expression of Runx2 and Osx showed downregulation of transcription of both genes under normoglycemic conditions. Our data also indicate that hyperglycemic conditions potentiate downregulation of Runx2 and Osx mRNA by myostatin. MC3T3‐E1 pre‐osteoblast cells possess functional myostatin‐activated signaling pathways via Smad2 phosphorylation. Myostatin negatively regulates transcription of genes involved in osteoblast differentiation, such as RUNX2 and Osx, and these effects are more pronounced in the presence of hyperglycemia. These experiments may be of particular clinical relevance when evaluating the effects of myostatin on bone cells in hyperglycemic conditions, such as those found in diabetes mellitus.


**Japneet Kaur, PhD (c)**



**Early Investigator Awardee*


University of Oklahoma, Norman, OK, USA

Relevant Session: Muscle‐Bone Interactions During Aging

DOI: 10.002/jbm4.10295


## Relationships Between Bone, Muscle, and Fat in Women Aged 18–85 Years

### Japneet Kaur,^1^ Zhaojing Chen,^2^ Ryan Miller,^1^ Eduardo Freitas,^1^ Debra Bemben,^1^ and Michael Bemben^1^


#### 
^1^Department of Health and Exercise Science, University of Oklahoma, Norman, OK, USA; ^2^Department of Kinesiology, California State University, San Bernardino, CA, USA

Previous research indicates that both lean and fat mass are equally associated with BMD or one is a better predictor than the other (Khosla et al., 1996). The aim of this study was to determine the relationships between BMD, bone free lean mass (BFLM), fat mass (FM), and muscle strength in pre‐ and postmenopausal women. The study included 139 women aged 18 to 85 years (*n* = 64 premenopausal [Pre‐MP] and *n* = 75 postmenopausal [Post‐MP]). Body composition (FM, percent body fat, and BFLM) and areal BMD for the total body, lumbar spine (LS), and proximal femur (femoral neck [FN], trochanter, total hip) were measured using DXA. PQCT was used to assess volumetric BMD (vBMD) and bone strength at 4%, 38%, and 66% of non‐dominant tibia. Handgrip test and jump test were used to assess muscle strength and power. After adjusting for covariates LS BMD (Pre‐MP: 1.231 ± 0.105 g/cm^2^, Post‐MP: 1.104 ± 0.174 g/cm^2^), trabecular bone strength index (BSI) at 4% (Pre‐MP: 50.58 ± 14.30 mg*mm, Post‐MP: 44.08 ± 12.11 mg*mm), and cortical vBMD at 38% (Pre‐MP: 1201.1 ± 18.0 mg/cm^3^, Post‐MP: 1160.5 ± 33.9 mg/cm^3^) and 66% (Pre‐MP: 1159.3 ± 20.2 mg/cm^3^, Post‐MP: 1107.4 ± 35.1 mg/cm^3^) of tibia were significantly greater for pre‐ than for postmenopausal women (*p* = 0.01). Linear regression analyses showed that BFLM had significant positive associations with LS BMD, left FN BMD, trabecular vBMD, and BSI at 4% of tibia for premenopausal women, and strength strain index (SSI) and polar moment of inertia (iPOLAR) at 38% and 66% of tibia for both pre‐ and postmenopausal women (*p* = 0.01). Handgrip strength and jump time in air were positively associated with SSI, iPOLAR, and total vBMD at 38% and 66% of tibia for pre‐ and postmenopausal women (*p* = 0.001). Fat mass showed a positive association with total vBMD at 4% and a negative association with cortical vBMD at 66% of tibia for premenopausal women (*p* = 0.01). Inflection points revealed that increase in FM above 18 kg was negatively associated with vBMD or at least became less favorable. Thus, both BFLM and FM are associated with bone parameters depending on skeletal site and age; however, FM has beneficial effects on bone only until a certain point after which it becomes less advantageous. Therefore, clinicians should target increasing BFLM and muscle strength and decreasing fat mass for optimal bone health.


**Mariana Kersh, PhD**


Beckman Institute, University of Illinois, Urbana, IL, USA

DOI: 10.002/jbm4.10296


## Building Better Bones Using Multi‐Scale Musculoskeletal Models

### Mariana E Kersh^1,2^


#### 
^1^Department of Mechanical Science and Engineering, University of Illinois at Urbana‐Champaign; ^2^Beckman Institute of Advanced Science and Engineering, University of Illinois at Urbana‐Champaign

The notion that bone responds to mechanical forces has been well established, but understanding the relationship between muscle‐driven forces on bone—and the adaptation to such forces—is not as well understood. This lack of clarity is due in part to the inability to measure the resultant mechanical stimulus at the cellular level to induce a response from osteocytes embedded within the bone matrix. However, computational models present an inviting opportunity to explore the propagation of muscle forces to meso‐ and micro‐scale models of bone in order to investigate the mechanobiology of bone (re)modeling. We have used these computational approaches to explore the strain and strain energy in bone (mechanical stimuli measures for adaptation) in healthy growing bone and in aged bone to further elucidate the muscle‐bone connection. Using animal models of growth, we have shown that strain energy resulting from everyday forces during locomotive activities is modulated more by material properties (apparent mineral density) than structural properties (bone area fraction or cortical thickness). At the meso‐scale, these properties are heterogeneous and vary between anatomical quadrants suggesting that typical loading during growth results in focally specific adaptations. These results now serve as the benchmark from which exercise interventions can be targeted to induce an adaptive response. The development of such interventions requires an understanding of which muscle groups result in increased strain energy within the desired region of bone. Using subject‐specific models of postmenopausal women, we have identified muscle groups that may serve as targets for interventions designed to improve bone strength within the proximal femur. Based on our analyses of a variety of tasks, activities that include increased hip and knee flexion and therefore use of gluteus maximum and vasti muscles result in increased strains within the femoral neck. Importantly, the ground reaction force during these tasks was significantly correlated with strain within the femoral neck and may be a useful clinical measure of an exercise designed to induce increased strains. These results highlight the utility of musculoskeletal and finite‐element models to further our understanding of how muscle and joint reaction forces induce a mechanical stimulus in bone.


**Allie Kemp, PhD**


Indiana University, Indianapolis, IN, USA

Relevant Session: Biomechanical Relationships Between Muscle and Bone

DOI: 10.002/jbm4.10297


## Physical Activity Benefits Bone Microarchitecture and Strength at the Distal Radius: A Within‐Subject Controlled HRpQCT Study

### Allie C Kemp, Christian S Wright, Robyn K Fuchs, and Stuart J Warden

#### Department of Physical Therapy, School of Health & Human Sciences, Indiana University, Indianapolis, IN, USA

Fractures of the distal radius are common across the lifespan, typically occurring due to a fall onto an outstretched hand. Risk is particularly increased in the elderly due to concomitant osteoporosis, which compromises the underlying skeletal structure and strength. One way to strengthen the skeleton is through physical activity. Previous studies have used within‐subject controlled models to demonstrate the benefit of physical activity on distal radius bone mass and size. Within‐subject controlled studies assess side‐to‐side differences (ie, bilateral asymmetry) in individuals who preferentially exercise one side of the body, enabling the skeletal effects of physical activity to be explored in the absence of selection bias and with lessened impact of inherited and systemic factors. To date, no studies have explored the benefit of physical activity on distal radius bone microarchitecture and finite element estimated strength. We recruited cohorts of collegiate‐level tennis (*n* = 13) and cross‐country (*n* = 12; control) athletes to explore side‐to‐side differences in cortical and trabecular bone properties at the distal radius utilizing high‐resolution (60.7‐micron voxel size) peripheral quantitative computed tomography (HRpQCT). The dominant arm in each individual was their racquet arm or the arm they would prefer to throw or hit a ball with. After performance of a scout scan, a reference line was placed at the medial edge of the distal radius joint surface and 168 slices (10.2 mm of bone length) were acquired centered 4% of bone length proximal to the reference line. Control (cross‐country) athletes did not exhibit dominant‐to‐nondominant arm differences in any assessed property (all *p* > 0.05), indicating minimal impact of simple arm dominance on distal radius bone properties. In contrast, tennis players exhibited large dominant‐to‐nondominant arm differences in distal radius size, cortical and trabecular bone properties, and estimated strength. Total bone area and cortical area and thickness were 7.2% (3.5% to 10.8% [95% confidence interval]), 12.7% (6.9% to 18.5%) and 11.7% (5.0% to 18.5%) greater in dominant versus nondominant arms in tennis players. Dominant versus nondominant arms in tennis players had 11.8% (7.6% to 16.1%) more trabecular bone (BV/TV) as a result of increased trabecular thickness (37%; 1.8% to 5.6%) as opposed to greater numbers of trabeculae (0.8%; −2.1% to 3.7%). These cumulative size and cortical and trabecular differences endowed the distal radius in the dominant arm of tennis players with 19.4% (13.5% to 25.3%) and 21.0% (14.6% to 27.4%) greater estimated fracture load and stiffness, respectively. These data indicate the benefit of physical activity on bone microarchitecture and strength at the distal radius and extend previous observations by revealing that prolonged unilateral physical activity increases the size/thickness of trabeculae, as opposed to increasing how many trabeculae are present.


**Yukiko Kitase, DDS, PhD**


Indiana University, Indianapolis, IN, USA

DOI: 10.002/jbm4.10298


## HIF‐Mediated Metabolic Reprogramming and Mitochondria Quality Control in Osteocytes

### Yukiko Kitase,^1,2^ Matt Prideaux,^1,2^ and Lynda Bonewald^1,2^


#### 
^1^Indiana Center for Musculoskeletal Health, Indianapolis, IN, USA; ^2^Anatomy and Cell Biology, Indiana University, Indianapolis, IN, USA

Homeostasis of energy metabolism is critical for cellular function, but there are few studies on energy metabolism of osteocytes that reside within a mineralized matrix. We hypothesized that cellular energy metabolism is altered during the transition from osteoblasts to osteocytes in order for osteocytes to dynamically adjust to a hypoxic environment. This is accomplished by reprogramming energy metabolism and maintaining mitochondria integrity. Using the IDG‐SW3 cell line that reproduces late osteoblast to late osteocyte differentiation, we performed RNA‐Seq and compared Day 28 (late osteocytes) with Day 4 (osteoblasts). Upregulation of the Phd/Hif pathway in Day 28 osteocytes compared with Day 4 osteoblasts shows that these cells are experiencing hypoxia. Under limited oxygen, strict regulation of ROS is required to maintain cell viability. Ineffective electron transfer induces electron leakage, resulting in damaging levels of ROS in mitochondria. Three HIF‐regulated mechanisms reducing ROS production were observed: **1) Glycolytic cellular energy metabolism.** ATP counteracts loss of mitochondrial membrane depolarization that can lead to apoptosis. Glycolytic adaptation as determined by expression of key HIF‐regulated glycolytic genes was strongly induced in osteocytes compared with osteoblasts; Slc2a10 (3.9‐fold, Log2FPKM = 3.5), Pgk1 (6.2, 7.1), Ldha (3.2, 8.6), and Slc16a3 (153, 4.5). Pdk1 that negatively regulates glucose entry to the mitochondria showed a 5‐fold induction. This suggests that osteocytes produce ATP anaerobically. Cpt1a/c that controls the entry of fatty acid (FA) to mitochondria for β‐oxidation was downregulated 2.5‐fold, suggesting inhibition of energy substrate oxidation. Suppression of FA entry should lead to suppression of oxidative phosphorylation, which also limits ROS production. **2) Mitochondria respiratory chain.** Ndufa4l2 and Cox4i2 were elevated 46‐ and 135‐fold, respectively. Both Ndufa4l1 and Cox4i2 were reported to minimize ROS production by inhibiting Complex I activity and by maximizing the efficiency of mitochondrial respiration under hypoxia. **3) Mitochondrial quality control.** Bnip3, which regulates mitochondrial integrity by inducing fission and mitophagy to remove damaged mitochondria, was increased by 8‐fold. The present study indicates that during osteoblast to osteocyte differentiation, cellular energy metabolism is reprogrammed and mitochondrial quality‐control systems are modified to adapt to an increasing hypoxic environment. HIF‐mediated protective mechanisms may play a pivotal role to maintain osteocyte long life and function.


**Ben Kirk, PhD**



**Early Investigator Awardee*


University of Melbourne and Western Health, St Albans, Melbourne, Australia

Relevant Session: Muscle‐Bone Interactions During Aging

DOI: 10.002/jbm4.10299


## Osteosarcopenia Impairs Balance in Community‐Dwelling Older Adults

### Ben Kirk,^1,2^ Steven Phu,^1,2^ Ebrahim Bani Hassan,^1,2^ Ahmed Al Saedi,^1,2^ Sara Vorgin,^1,2^ Sharon Brennan,^1,2^ and Gustavo Duque^1,2^


#### 
^1^Australian Institute for Musculoskeletal Science (AIMSS), University of Melbourne and Western Health, St Albans, Melbourne, Australia; ^2^Department of Medicine, Western Health, Melbourne Medical School, University of Melbourne, St Albans, Melbourne, Australia

Osteosarcopenia is a geriatric syndrome characterized by low bone mass (osteoporosis) and low muscle mass and function (sarcopenia), which may lead to a greater risk of falls and fractures in comparison to either disease alone. As such, this study aimed to investigate the impact of osteosarcopenia on postural control and unearth modifiable risk factors in community‐dwelling older adults. A total of 235 community‐dwelling, older adults (78% female; age: median 78 years [IQR: 73, 83] years; BMI: 27.32 [23.83, 31.01] kg/m^2^; ALM: 6.36 [5.73, 7.18] kg/m^2^; BMD: −2.9 [−3.5, −2.1]; vitamin D [vit D]: 70 [55, 85] nmol/L; parathyroid hormone [PTH]: 6.8 [5.0, 10.3] pmol/L) were diagnosed as osteosarcopenic or nonosteosarcopenic. Appendicular lean mass (measured by dual‐energy X‐ray absorptiometry), handgrip strength (hydraulic dynamometer), and usual gait speed (over 4 meters) were utilized for diagnostic criteria, while postural control was evaluated by the 3D virtual reality Balance Rehabilitation Unit (BRU), and the number of falls and fractures within the past 12 months were self‐reported. Blood test (via chemiluminescence immunoassay) were used to control for vit D and PTH concentrations during modeling analyses. Prevalence of osteosarcopenia and sarcopenia was 14.2% (*n* = 35) and 20% (*n* = 47) in our respective population. Posturography comparisons revealed a greater proportion of osteosarcopenic (*n* = 25, 71.4%) versus nonosteosarcopenic (*n* = 106, 51%) older adults were unable to complete the “stand on foam with eyes closed” assessment (*p* = 0.028; odds ratio = 0.42; 95% CI 0.19–0.91). After adjusting for age, sex, BMI, vit D, and PTH, these values remained significant (*p* = 0.046; odds ratio = 0.40; 95% CI 0.17–0.98). However, no between‐group differences were observed for other BRU parameters, falls, or fractures (*p* > 0.05). Osteosarcopenia conferred a greater decrement of postural control in older adults. Implications from these findings suggest rehabilitation programs focus on improving balance performance in order to reduce the risk of injurious falls and fractures in this population.


**Michael Klüppel, PhD**


Indiana University, Indianapolis, IN, USA

Relevant Session: Role of Muscle and Bone Factors in Energetics and Metabolism

DOI: 10.002/jbm4.10300


## Novel Roles of Atrogin‐1 in Cardiac Disease, Lipid Metabolism, and Bone Microstructure

### Michael Klüppel,^1,2^ Vidyani Suryadevara,^1,2^ Jessica M Berthiaume,^1,2^ and Monte Willis^1,2,3^


#### 
^1^Department of Pathology and Laboratory Medicine, Indiana University School of Medicine, Indianapolis, IN, USA; ^2^Indiana Center for Musculoskeletal Health, Indiana University School of Medicine, Indianapolis, IN, USA; ^3^Krannert Institute of Cardiology and Division of Cardiology, Department of Internal Medicine, Indiana University School of Medicine, Indianapolis, IN, USA

Differential ubiquitination plays a critical role in controlling proteasomal degradation, subcellular localization, and activity of proteins. A functional role for the ubiquitin ligase Atrogin‐1 (MAFbx) has been reported in skeletal muscle and the heart but not in other organs. Within the myocyte, Atrogin‐1 localizes to the sarcomere and nucleus. Our group has reported that Atrogin‐1 is a critical regulator of pathologic and physiologic cardiac hypertrophy in vivo, involving specific interaction and ubiquitination of transcription factors central the signaling pathways driving these hypertrophic responses, including FOXO1/3. Recent studies have linked cardiomyocyte Atrogin‐1 to the regulation of the extracellular matrix. With evidence that cardiomyocyte Atrogin‐1 can affect cells other than myocytes, we analyzed Atrogin‐1−/− mice for their effects on metabolism and bone. DXA whole body analysis of Atrogin‐1−/− mice revealed significantly decreased fat mass (15.6% versus 35.8% fat) with a corresponding increase in lean mass (84.4% versus 64.2%) at the age of 11 to 15 months, but not at 5 months, when compared with strain‐ and age‐matched wild‐type (WT) control mice. Since the Atrogin‐1−/− mice weighed significantly less than WT mice (29 + 3 versus 40 + 2 g), the net fat loss was 4.6 g (versus 14.3 g in age‐matched WT) and the lean body mass identical between groups (25.7 versus 24.6 g). Together these findings suggest Atrogin‐1's role in regulating fat metabolism. Analysis of bone microarchitecture and mechanical properties of adult Atrogin‐1−/− mice was undertaken at 17 to 21 weeks of age, a time point where no phenotype has been previously observed in skeletal muscle or heart. Femurs were dissected and subsequently assessed by microCT (SkyScan 1172) for bone microarchitecture in the distal femur metaphysis (1 mm) and mid‐diaphysis region and challenged by a three‐point bending test to assess their material and structural properties. No differences in cortical or trabecular bone microarchitecture were identified and nor were any changes in mechanical properties. These findings illustrate novel biological roles of Atrogin‐1 in regulating systemic fat metabolism possibly involving cross‐talk between skeletal muscle/heart and systemic fat metabolism. Our analysis provides an essential framework for the potential therapeutic targeting of ubiquitin ligases like Atrogin‐1 in the context of striated muscle and metabolic disease.


**Tatiana Kostrominova, PhD**


Indiana University, Gary, IN, USA

Relevant Session: Muscle‐Bone Interactions in Orthopedics

DOI: 10.002/jbm4.10301


## Endoplasmic Reticulum Stress‐Induced Activation of Unfolded Protein Response in Myoblasts and Myotubes

### TY Kostrominova

#### School of Medicine – Northwest, Indiana University, Gary, IN, USA

The endoplasmic reticulum (ER) is an important component of skeletal muscle adaptation to physiological and pathological conditions. Accumulation of misfolded proteins leads to increased ER stress and to the activation of adaptive cellular response: unfolded protein response (UPR). UPR includes activation of Inositol‐requiring enzyme (IRE1), protein kinase RNA‐like endoplasmic reticulum kinase (PERK), and activating transcription factor 6 (ATF6). Effective upregulation of ER stress‐induced UPR protects cells from damage, while inadequate or persistent UPR can lead to cell death. We compared the effect of Tunicamycin (Tnm), a well‐known ER stress inducer, on L_6_ rat skeletal muscle myoblasts and myotubes. Tnm treatment increased mRNA expression of IRE1 (2.2‐fold) and CHOP (16.4‐fold) in myoblasts, and significantly higher (IRE1: 3.3‐fold; CHOP: 20‐fold) in myotubes. Tnm treatment also increased CHOP protein expression in both myoblasts (8‐fold) and myotubes (9‐fold) as well as CHOP nuclear translocation. mRNA expression of ATF4, ATF6, XBP1, BiP, and ERdj4 were also significantly increased after Tnm treatment in both myoblasts and myotubes. There were no differences between myoblasts and myotubes in Tnm‐induced activation of these genes. Interestingly, mRNA expression levels of BiP, ATF4, and ATF6 were ~2‐fold higher in nontreated control myotubes when compared with myoblasts. ATF6 protein expression also was ~2‐fold higher in nontreated control myotubes when compared with myoblasts. There was a trend to higher protein expression of BiP in control myotubes when compared with myoblasts, but it did not reach statistical significance. BiP immunostaining also indicates higher expression levels in myotubes when compared with myoblasts. In summary, both myoblasts and myotubes have a very similar activation pattern of ER stress‐induced UPR. Myotubes have higher basal levels of expression of some ER stress‐related genes. This might reflect a higher level of protein synthesis/modification observed in myotubes.


**H. Jean Kok, PhD Candidate**


University of Florida, Gainesville, FL, USA

DOI: 10.002/jbm4.10302


## Muscle Viral Delivery of IGF‐1 in Mice Alters the Responses of Muscle and Bone to Suspension and Reloading

### Hui Jean Kok,^1,5^ Lauren Lautenslager,^1^ Raymond Duong,^4^ Joshua Yarrow,^3,5^ and Elisabeth Barton^1,2,4^


#### 
^1^Departments of Applied Physiology and Kinesiology, and Pharmacology and Therapeutics; ^2^Division of Diabetes, Endocrinology, and Metabolism, University of Florida, Gainesville, FL, USA; ^3^Anatomy and Cell Biology, School of Dental Medicine, University of Pennsylvania, Philadelphia, PA, USA; ^4^Research Service Malcom Randall VA Medical Center, North Florida/South Georgia; ^5^Veterans Health System, Gainesville, FL, USA

Prolonged disuse of skeletal muscle results in atrophy, and once physical activity is resumed, there is increased susceptibility to injury. Similarly, bone also suffers from disuse, with the most aggressive loss of trabecular bone density compared with cortical bone thickness. Insulin‐like growth factor‐I (IGF‐I) is considered a potential therapeutic target to attenuate atrophy during unloading and to enhance rehabilitation upon reloading of the musculoskeletal system. While systemic delivery of IGF‐I seems protective only with loading, local production of IGF‐I by muscle may provide a more efficient source of benefit for both tissues. To determine if increased IGF‐I in muscle contributes to remodeling of both muscle and bone during disuse and reloading, unilateral intramuscular viral delivery of Igf1 was performed in mice, which were then subjected to hindlimb suspension and reloading. Self‐complementary adeno‐associated virus harboring the murine proIgf1 cDNA was delivered to hindlimbs of adult female C57BL6 mice 3 days before hindlimb suspension. Hindlimb muscles were unloaded for 7 days and then reloaded for 3, 7, and 14 days. Loss of soleus mass and force after suspension was not prevented by IGF‐I. Nevertheless, soleus muscles showed a 10% to 30% increase in mass and force‐generating capacity at 7 and 14 days reloading due to increased IGF‐I. Trabecular bone density decreased by ~50% in response to suspension and was rescued in IGF‐1‐treated limbs across all groups by 10% to 35%. Minimal changes in cortical bone thickness were observed. This study supports that skeletal muscle integrity contributes to skeletal properties and implicates IGF‐I as an important factor in muscle and bone interaction. Future work will be performed to delineate if our current finding is due to mechanical or chemical coupling between muscle, bone, and IGF‐I.


**Kent Leach, PhD**


Indiana University, Indianapolis, IN, USA

Relevant Session: Muscle‐Bone Interactions in Orthopedics

DOI: 10.002/jbm4.10303


## Conditioning of Myoblast Secretome Using Mesenchymal Stem/Stromal Cell Spheroids Improves Bone Repair

### Augustine M Saiz Jr,^1^ Marissa A Gionet‐Gonzales,^2^ Mark A Lee,^1^ and J Kent Leach^1,2^


#### 
^1^Department of Biomedical Engineering, University of California at Davis, Davis, CA, USA; ^2^Department of Orthopedic Surgery, UC Davis Health, Sacramento, CA, USA

Local muscle loss associated with open fractures remains an obstacle to functional recovery and bone healing. Muscle cells secrete bioactive myokines that elicit autocrine and paracrine effects and initiate signaling pathways for regenerating damaged muscle and bone. Mesenchymal stem/stromal cells (MSCs) are under investigation for the regeneration of both muscle and bone through their potent secretome. Compared with monodisperse cells, MSC spheroids exhibit a more complex secretome with heightened therapeutic potential. We hypothesized that the osteogenic potential of myokines would be enhanced when myoblasts were exposed to the MSC spheroid secretome. Conditioned media from MSC spheroids increased osteogenic response of MC3T3 pre‐osteoblasts compared with myokines from L_6_ myoblasts alone. This effect was synergistically enhanced when conditioned media of MSC spheroids was serially delivered to myoblasts and then osteoprogenitor cells in vitro. We then delivered myoblast‐stimulated conditioned media in the presence or absence of syngeneic rat bone marrow stromal cells (rBMSCs) from alginate hydrogels to a rat critical‐sized segmental defect. We observed increased bone formation in defects treated with conditioned media compared to rBMSCs alone, while bone formation was greatest in defects treated with both conditioned media and rBMSCs over 12 weeks. This study demonstrates a novel approach for capitalizing on the paracrine signaling of muscle cells to promote bone repair and provides additional evidence of the synergistic interaction between muscle and bone.


**Todd McKinley, MD**


Indiana University School of Medicine, Indianapolis, IN, USA

DOI: 10.002/jbm4.10304


## Polytraumatized Rats with Bone and Muscle Injury

### Roman Natoli,^1^ George Kolettis,^1^ Jared Smith,^1^ Kayla Delaney,^1^ Jie Xie,^1^ Fletcher White,^1^ Ophir Ortiz,^2^ Philip Chan,^2^ and Todd McKinley^1^


#### 
^1^Indiana University School of Medicine, Indianapolis, IN, USA; ^2^Cytosorbents, Monmouth, NJ, USA

Multiply injured patients sustaining severe extremity trauma are at risk of wound failure, including nonunion, infection, and muscle loss. The systemic immunologic response may affect localized response in injured bone and muscle. Blood purification by selective extracorporeal hemoadsorption has been shown to reduce circulating levels of cytokines after injury. We tested the hypothesis that systemic blood purification would affect regional injury‐level immunologic response in a composite extremity wound. Male Sprague–Dawley rats approximately 350 to 400 g had open tibia fractures created by an open approach and cutting the bone. Fractures were stabilized with an intramedullary wire. Anterior compartment muscle adjacent to the fracture was crushed with a surgical snap. Subsequently, the rats were subjected to hemorrhagic shock by blood withdrawal to a systolic blood pressure of 40 mmHg for 60 minutes. After resuscitation, experimental rats received 120 minutes of extracorporeal circulation with the blood passing through a column of proprietary beads that selectively removed circulating cytokines (Cytosorbents; Monmouth, NJ). Control rats had no extracorporeal circulation. Rats were euthanized at 3 days (4 experimental; 4 control) and 7 days (5 experimental; 6 control) after surgery. Tissue was harvested from the injured muscle and from the hematoma/callus. The tissue was processed for flow cytometry focusing on typing immune cells (T cells, monocytes, PMNs). Hemoadsorption resulted in 10X to 20X increases in T cells in injured muscle at both 3 and 7 days postinjury, including CD4+, CD8+, and non CD4/CD8 cells. In callus tissue, only CD4+ cells were increased (10X) 3 days after injury. Likewise, hemoadsorption increased monocytes by 10X to 30X in injured muscle 7 days after injury. In addition, hemoadsorption reduced the ratio of inflammatory/anabolic monocytes at both 3 days and 7 days after injury in muscle. No changes in monocytes were measured in the callus. Hemoadsorption reduced PMNs in callus by 10X 3 days after injury. Hemoadsorption had significant effects on immune cell trafficking primarily in injured muscle in multiply injured rats with severe limb trauma. Increasing cytokine gradients between the circulating compartment and the injury compartment may affect cell migration during the acute injury time period.


**Luke Mortensen**


University of Georgia, Athens, GA, USA

DOI: 10.002/jbm4.10305


## Mitochondrial Dysfunction May Contribute to Muscle Weakness in Hypophosphatasia

### Luke J Mortensen,^1,2^ Anna S Nichenko,^1^ Jarrod A Call,^1^ and Emily G Pendleton^1^


#### 
^1^University of Georgia Regenerative Bioscience Center; ^2^University of Georgia School of Chemical, Materials, and Biomedical Engineering

Hypophosphatasia (HPP) is a rare metabolic bone disorder that is characterized by low levels of tissue nonspecific alkaline phosphatase (TNALP). Clinical manifestations of HPP include demineralization of the bone, which can lead to bone deformity and fractures. In addition, patients often complain of chronic muscle pain, reduced muscle strength, and an altered gait. To probe at the cause of muscular pain in HPP patients, we used a murine model with juvenile‐onset HPP to evaluate mitochondrial function. The physiological responses of the skeletal muscle was weaker in the HPP mouse than the healthy siblings. The mitochondrial respiration of skeletal muscle was also significantly different in the HPP mouse when compared with healthy siblings. Using two‐photon microscopy, we evaluated the formation of mitochondrial networks and found that the HPP muscle showed a pattern distinct from healthy muscle. With the same imaging technique, we evaluated calcium flux in skeletal muscle when live muscles were stimulated ex vivo. Again, there were marked differences between the responses of healthy and HPP muscle. Our work here suggests that HPP leads to mitochondrial dysfunction in skeletal muscle, thus creating a novel target in the treatment of HPP.


**Sara Moshage, MS (c)**


University of Illinois at Urbana‐Champaign, Urbana, IL, USA

Relevant Session: Biomechanical Relationships between Bone and Muscle

DOI: 10.002/jbm4.10306


## Spatial Heterogeneity in Bone Structure and Composition During Growth

### Sara G Moshage,^1^ Annette M McCoy,^2^ John D Polk,^3^ and Mariana E Kersh^1,4^


#### 
^1^Department of Mechanical Science and Engineering, University of Illinois at Urbana Champaign (UIUC); ^2^Department of Veterinary Clinical Medicine, UIUC; ^3^Department of Anthropology, UIUC; ^4^Beckman Institute for Advanced Science and Technology

Bone is most responsive to mechanical loads during growth,^(1)^ when the skeletal system reorganizes its structure and composition to increase in size and mass. Exercise at an early age increases bone strength, but the amount and intensity of needed exercise is unknown. Establishing a baseline measure of bone development will assist exercise programs used to direct bone growth. Therefore, the goal of this study was to evaluate the degree of spatial heterogeneity in bone structure and composition during growth. After IACUC approval, the left forelimbs of 3 male Standardbred trotter foals were CT scanned between birth and 1 year of age (18 scans total). Hydroxyapatite phantoms were used to calculate apparent mineral density, and previously established thresholds were used to identify cortical and trabecular bone voxels. Bone area fraction (expressed as %) and apparent density were measured at the diaphysis, distal, and proximal regions of the proximal phalanx (P1) bone. Cortical, trabecular, and density accrual rates were examined at the cross‐section level, as well as within anatomical quadrants (dorsal, palmar, lateral, and medial). Within the distal epiphysis, bone (re)modeling during growth resulted in an apparent increase in cortical bone and simultaneous decrease in trabecular bone resulting in the lowest net bone accrual rate (0.012%/kg, *p* < 0.05). This pattern was also observed at the proximal epiphysis, but the degree of trabecular bone loss was lower, resulting in a doubling of the bone accrual rate compared with the distal region. Within the diaphysis, the cortical bone accrual rate was lower than in the epiphyses, but there was significantly less trabecular bone loss, resulting in the overall highest net accrual rate (0.048%/kg, *p* < 0.01). The diaphysis had the densest cortical bone compared with the epiphyses with maximum densities found in the medial and lateral quadrants. Diaphyseal cortical density increased at similar rates in all quadrants. These data suggest that bone (re)modeling during growth within the P1 increases the structural and material properties within the diaphysis compared with the epiphyses and is preferentially directed mediolaterally, which may serve to enhance resistance to bending loads during locomotion.^(3)^


## References


Firth EC, et al. Mild exercise early in life produces changes in bone size and strength but not density in proximal phalangeal, third metacarpal and third carpal bones of foals. Vet J. 2011;190(3):383–9.Verheyen KLP, Wood JLN. Descriptive epidemiology of fractures occurring in British thoroughbred racehorses in training. Equine Vet J. 2004;36(2):167–73.Les CM, et al. The distribution of material properties in the equine third metacarpal bone serves to enhance sagittal bending. J Biomech. 1997;30(4):355–61.



**Roman Natoli, MD, PhD**


Indiana University School of Medicine, Indianapolis, IN, USA

DOI: 10.002/jbm4.10307


## Development and Characterization of a Porcine Model of Combined Segmental Bone Defect and Volumetric Muscle Loss to Examine Longitudinal Muscle Strength Changes Resulting From Injury

### RM Natoli,^1^ A Brinker,^1^ G Adam,^1^ Z Gunderson,^1^ N Diggins,^1^ S Seungyup,^1^ A Tucker,^1^ B Corona,^2^ MA Kacena,^1^ and OT McKinley^1^


#### 
^1^Indiana University School of Medicine, Department of Orthopaedic Surgery; ^2^Wake Forest School of Medicine

Major extremity limb trauma can cause bone and muscle loss. These injuries often result in a limb that has decreased strength compared with an uninjured limb. Better understanding of changes that occur in muscle strength after volumetric muscle loss (VML) adjacent to a segmental bone defect (SBD) could eventually translate to improved patient function. In this abstract we present a translational model simulating major extremity injury. Seventeen male Yucatan miniature pigs were subjected to a SBD of 2.5 cm stabilized with internal fixation via plate and screw construct (control) or the addition of ~7 g VML from the anterior compartment (experimental). VML was created either as partial or full thickness defect. Thirteen pigs had scaffolds implanted in the SBD that yielded a robust inflammatory reaction (*n* = 3 SBD, *n* = 5 SBD + partial VML, *n* = 5 SBD + full‐thickness VML), whereas 4 did not have scaffolds placed (*n* = 2 SBD, *n* = 2 SBD + partial VML). A customized, in vivo muscle testing apparatus was used to measure maximum torque generated by the anterior compartment muscle before injury and at 1, 2, and 3 months post‐surgery. A qualitative data synthesis is presented. In all pigs, there is a sharp decline in peak torque from pre‐injury to 1‐month post‐surgery. Muscle strength did not recover by the 3 months post‐surgery, irrespective of partial or full thickness VML, in limbs with an inflammatory reaction (<20% pre‐injury state). Gross inspection revealed substantial muscle fibrosis. At 3 months, all VML limbs had decreased peak torque generation compared with those without muscle injury. Interestingly, creation of a SBD itself resulted in decreased muscle strength that recovered only to ~55% to 65% of pre‐injury state by 3 months. We present a translational, porcine model of major extremity limb injury with the ability to test muscle strength recovery in vivo. It appears that an SBD alone is enough to decrease muscle strength that does not fully recover by 3 months, likely due to relative extremity disuse. Further, an inflammatory reaction is clearly detrimental to injured muscle strength recovery. This model can be used to further explore muscle‐bone interactions after major extremity injury.


**Hari Nakshatri, BVSc, PhD**


Indiana University, Indianapolis, Indiana.

DOI: 10.002/jbm4.10308


## Myogenic microRNA miR‐486: A Common Thread Between Cancer and Cancer‐Induced Skeletal Muscle Dysfunction

### Ruizhong Wang,^1^ Brijesh Kumar,^1^ Poornima Bhat‐Nakshatri,^1^ Mayuri S Prasad,^1^ Trupti Trivedi,^1^ Max Jacobson,^1^ Gabriela Ovalle,^1^ Calli Maguire,^1^ George Sandusky,^1^ Narsimha Penthala,^2^ Peter A Crooks,^2^ Jianguo Liu,^1^ Khalid S Mohammad,^1^ Theresa Guise,^1^ Teresa Zimmers,^1,3^ and Harikrishna Nakshatri^1,3^


#### 
^1^Indiana University School of Medicine, Indianapolis, IN, USA; ^2^University of Arkansas, Little Rock, AR, USA; ^3^VA Roudebush Medical Center, Indianapolis, IN, USA

Breast cancer progression is associated with systemic defects including functional limitations and myopenia. Autocrine/paracrine actions of cancer‐derived cytokines/chemokines mediate cancer progression and functional limitations. The cytokine‐inducible transcription factor NF‐κB could be central to this process, as it displays oncogenic functions and is integral to the Pax7:MyoD:Pgc‐1β:miR‐486 myogenesis axis. We reported lower circulating levels of the myogenic microRNA miR‐486 in breast cancer patients compared with healthy women. Similar changes in circulating levels as well as skeletal muscle levels of miR‐486 were observed in mammary tumor models. This study was designed to examine the effects of pharmacologic and genetic manipulation of miR‐486 levels on skeletal muscle functions. MMTV‐PyMT and MMTV‐Neu mice were used as tumor models, whereas MCK‐miR‐486 transgenic mice were used to genetically manipulate skeletal muscle miR‐486 levels. The NF‐κB inhibitor dimethylaminoparthenolide (DMAPT) was used to pharmacologically alter miR‐486 expression. Functional limitation studies and biochemical studies of skeletal muscle were performed to assess the impact of cancer and therapeutic interventions on skeletal muscle function. We observed aberrations in skeletal muscle of tumor‐bearing mice with accompanying functional limitations, which were reversed partially by DMAPT. Comparative analyses of two models revealed breast cancer subtype‐specific molecular differences in skeletal muscle. While tumor‐bearing mice in both models showed lower muscle contraction force, increased ECM deposition, and reduced p53 signaling, which is essential for maintaining muscle satellite‐progenitor‐myotube hierarchy, other molecular targets in two models were different. While skeletal muscle of PyMT+ mice showed mitochondrial defects, Neu + mice displayed accelerated aging‐associated changes including shrinkage of muscle fibers. Circulating cytokine/chemokine profiles were also different in two models. DMAPT restored circulating levels of muscle‐enriched microRNA miR‐486 only in Neu + mice. In Neu + but not PyMT+ model, skeletal muscle‐specific overexpression of miR‐486 was sufficient to overcome cancer‐induced skeletal muscle defects. Cancer‐specific genomic aberrations have an impact on the type of molecular and structure changes in skeletal muscle, which are amicable for pharmacologic interventions in only specific cases. This cancer‐specificity could explain for the lack of clinical success of cachexia‐targeted clinical trials, as these trials did not integrate cancer genomics with symptom science management to individualize therapies.


**Ashok Narasimhan, PhD**


Indiana University, Indianapolis, IN, USA

DOI: 10.002/jbm4.10309


## Profiling of Matched Adipose and Skeletal Muscle Tissue in Patients with Pancreatic Cancer Cachexia

### Ashok Narasimhan,^1^ Xiaoling Zhong,^1,7^ Ernie Au,^1,2^ Eugene Ceppa,^1^ Atilla Nakeeb,^1^ Michael House,^1,7,9^ Nicholas Zyromski,^1^ C Max Schmidt,^1^ Katharyn Hannaford,^1^ Daniel Schloss,^1^ Yunlong Liu,^3,7,8,9,10^ Guanglong Jiang,^3^ Brad Hancock,^1^ Milan Radovich,^1,9^ Joshua Kays,^1^ Safi Shahda,^4,7,9^ Marion E Couch,^5,7,9^ Leonidas G Koniaris,^1,7,9,10^ and Teresa A Zimmers^1,2,5,6,7,8,9,10^


#### Departments of ^1^Surgery, ^2^Biochemistry and Molecular Biology, ^3^Medical and Molecular Genetics, ^4^Medicine—Hematology and Oncology, ^5^Otolaryngology—Head and Neck Surgery, and ^6^Anatomy and Cell Biology, and Surgery, Indiana University School of Medicine, Indianapolis, IN, USA; ^7^IUPUI Center for Cachexia Research Innovation and Therapy, ^8^Center for Computational Biology and Bioinformatics, ^9^Indiana University Simon Cancer Center, Indianapolis, IN, USA; ^10^Indiana Center for Musculoskeletal Health, Indianapolis, IN, USA

The vast majority of patients with pancreatic ductal adenocarcinoma (PDAC) suffer cachexia, a debilitating condition that reduces performance status, tolerance to anticancer therapies, and eventually affecting the quality of life and reducing survival. Although weight loss in cachexia results from concurrent loss of adipose and muscle tissue, most studies focus on muscle. Emerging data demonstrate prognostic value of fat loss, as well as implicate adipose tissue loss in muscle wasting, suggesting that adipose is an important, active component of cachexia. Here, our objective is to identify the muscle and adipose genes and pathways regulated in cachexia and to validate our findings against available external data sets. Matched rectus abdominis muscle and subcutaneous adipose tissue were obtained at surgery from patients with benign conditions (*n* = 11) and patients with PDAC (*n* = 23). Gene expression was measured by Ion proton sequencing using a panel of well‐characterized probes. Differentially expressed (DE) between controls and cancer were identified. Self‐reported weight loss and body composition measurements defined cachexia status. Ingenuity Pathway Analysis (IPA) were used for pathway analysis. The number of genes in adipose is ~5 times more than in muscle, indicating dynamic changes in adipose and may potentially precede muscle wasting. Most of the genes were unique to adipose and muscle demonstrating a tissue‐specific gene expression pattern. Although there were 9 signaling pathways common between adipose and muscle, the genes involved activating or inhibiting those pathways are predominantly different, emphasizing that adipose and muscle wasting may be mediated through independent mechanisms. For example, the top pathway, EIF2 signaling, had 87 genes from adipose but only 27 from muscle, with 18 genes in common. In both ours and the external data set, many well‐characterized genes in cachexia such as IL6R, ZIP14, and FOXO1 were identified in muscle. This is the first study to perform a matched muscle and adipose gene profiling from a single cancer type and validate the findings in external data set. We observed distinct, tissue‐specific gene expression profiles, providing potential therapeutic opportunities in targeting adipose wasting along with current preclinical and clinical trials that are in various phases for improving muscle wasting.


**Gabriel Pagnotti, PhD**


Indiana University, Indianapolis, IN, USA

DOI: 10.002/jbm4.10310


## Magnitude Mechanical Signals Suppress Bone and Muscle Loss in a Murine Model of Complete Estrogen Deprivation

### GM Pagnotti,^1^ R Pattyn,^1^ RE Wilson,^1^ LE Wright,^1^ T Trupti,^1^ S Murthy,^1^ SK John,^1^ Y She,^1^ WR Thompson,^1^ CT Rubin,^2^ KS Mohammad,^1^ and TA Guise^1^


#### 
^1^Indiana University, School of Medicine, Indianapolis, IN, USA; ^2^Stony Brook University, Department of Biomedical Engineering, Stony Brook, NY, USA

Estrogen (E2) receptor‐positive breast cancer patients are treated by complete E2‐deprivation using aromatase inhibitors to suppress peripheral E2 synthesis, thereby preventing further tumor growth. This effective breast cancer treatment is complicated by bone loss and musculoskeletal side effects. The bisphosphonate zoledronic acid (ZA) prevents bone loss and improves fracture risk in patients but its effects on other musculoskeletal adverse effects are not well studied. Mechanical signals generated during exercise are anabolic to bone and muscle; however, participation in regimented exercise programs by many patients proves difficult due to musculoskeletal weakness. Low‐intensity vibrations (LIV), mechanical signals similar to exercise‐generated stimuli, has prevented bone loss in cancer models and reduced fat accrual in ovariectomy models. We hypothesized that LIV could improve musculoskeletal morbidity in mice undergoing complete E2‐deprivation, such as that in postmenopausal breast cancer patients. To generate this model, 8‐week‐old C57Bl/6 mice ovariectomized were treated with the aromatase inhibitor letrozole daily for 24 weeks and were administered either LIV (*n* = 10; 90 Hz at 0.4 g) or control‐LIV treatment (CTL; *n* = 10) for 4 weeks before and 24 weeks post‐surgery. Whole body DXA scanning performed consecutively every 3 weeks measured lean and fat tissue. LIV significantly increased lean mass (*p* < 0.02) over the 28‐week treatment period relative to baseline, while LIV restricted total fat mass by 41% (*p* < 0.001). Forelimb grip strength was 32% greater (*p* < 0.05) in LIV‐mice versus CTL‐mice at 28 weeks. Histological sections of the quadratus femoris in LIV‐mice showed increased (*p* < 0.05) myofiber cross‐sectional area. High‐resolution (12 μm) ex vivo micro‐computed tomography was performed on lumbar vertebrae, a mechanosensitive load‐bearing site of bone metabolism. BV/TV and connectivity density of L_5_ vertebrae were 22% (*p* < 0.05) and 53% (*p* < 0.05) greater, respectively, after 24 weeks of LIV. Dynamic histomorphometry indicated a 70% (*p* < 0.05) greater BFR/BS and 60% greater trend in MS/BS in response to LIV. Osteoblast numbers were 38% (*p* < 0.01) greater in LIV‐treated L_5_ vertebrae than in CTL sections. TRAP‐stained sections indicated a 41% reduction in osteoclast surface (*p* < 0.01) and 37% (*p* < 0.05) fewer osteoclasts in LIV vertebrae. These data demonstrate the impact of E2‐deprivation on bone, muscle, and fat and the potential for mechanical signals to improve these parameters and reduce fracture risk.


**Lilian Plotkin, PhD**


Indiana University, Indianapolis, IN, USA

DOI: 10.002/jbm4.10311


## Getting Old Together: Interaction Between Bone and Muscle With Aging

### Lilian I Plotkin

#### Department of Anatomy, Cell Biology, and Physiology, Indiana University School of Medicine, and Indiana Center for Musculoskeletal Health, Indianapolis, IN, USA

Osteoporosis and sarcopenia are major complications affecting elderly individuals. However, whether there is a link between the onset of osteoporosis and sarcopenia with aging or whether one precedes the other in older individuals is unclear. Despite this uncertainty, it is clear that bone and skeletal muscle cells secrete factors that bidirectionally affect each other. Thus, myostatin, produced by skeletal muscle, reduces muscle mass and promotes osteoclast activity, although the role of this molecule with aging is unclear. Another muscle‐derived metabolite, β‐aminoisobutyric acid (BAIBA), has positive bone effects, preventing oxidative stress‐induced osteocyte apoptosis. Interestingly, although BAIBA levels are unchanged, the levels of its receptor Mas‐Related G Protein‐Coupled Receptor Type D (MRGPRD) decrease with aging, suggesting the involvement of BAIBA in bone deterioration with aging. Bone cell products can, in turn, affect skeletal muscle. For example, TGFβ, produced mainly by osteoblasts, stored in the bone matrix as inactive form and activated and released upon bone resorption, mediates cancer‐induced muscle wasting. Further, RANKL, a pro‐osteoclastogenic cytokine produced by osteoblasts and osteocytes, activates RANK in skeletal muscle and has been associated with induction of muscle dystrophy in dystrophin‐deficient mice. However, whether these bone‐produced cytokines are involved in the decrease of muscle mass with aging is unclear. Recent work of ours showed the involvement of the receptor for advanced glycation end products (RAGE) in the consequences of aging in the musculoskeletal system. Based on evidence indicating that RAGE expression/activation increases with aging, we administered Azeliragon (AZ), a small molecule that prevents RAGE from binding to its ligands, to aged mice. RAGE inhibition decreased both osteoblastic and osteoclastic parameters, without net changes in bone geometry or mechanical properties. On the other hand, AZ increased lean body mass and gastrocnemius, tibialis anterior, and quadriceps muscle weight. Interestingly, serum from vehicle‐treated aged mice decreased C2C12 myotube diameter when compared with serum from young animals, whereas serum from AZ‐treated old mice did not. Further, AZ directly affected bone cells, but not C2C12 myotubes in vitro, suggesting an indirect effect of RAGE inhibition on skeletal muscle. Whether these effects stem from AZ effects on bone cells in aging remains to be determined.


**Fabrizio Pin, PhD**


Indiana University, Indianapolis, IN, USA

DOI: 10.002/jbm4.10312


## Preservation of Bone Mass by Anti‐Resorptive Treatments Improves Skeletal Muscle Mass and Function in a Non‐metastatic Model of Cancer Cachexia

### Fabrizio Pin,^1^ Joshua Huot,^2^ Alyson L Essex,^1^ Alexander J Jones,^3^ Lynda F Bonewald,^1,4,5^ and Andrea Bonetto^1,2,3,4,5^


#### 
^1^Department of Anatomy and Cell Biology; ^2^Department of Surgery; ^3^Department of Otolaryngology–Head and Neck Surgery; ^4^Indiana Center for Musculoskeletal Health; ^5^Simon Cancer Center, Indiana University School of Medicine, Indianapolis, IN, USA

Cachexia, ie, progressive body weight loss and skeletal muscle depletion, affects up to 80% of all cancer patients. In a mouse model for the study of ovarian cancer (OC), we recently showed that cachexia also associates with marked bone loss, even when bone metastases are not present. Clinical evidence supports the idea that both tumor‐ and bone‐derived factors may directly regulate skeletal muscle size and function in cachexia. Here we aimed to investigate whether preservation of bone mass and/or inhibition of RANKL, a factor responsible for osteoclast‐dependent bone resorption, can improve skeletal muscle wasting during OC‐induced cachexia. NSG mice were injected intraperitoneally with 1 × 107 ES‐2 human high‐grade serous OC cells. Both control and ES‐2 tumor‐bearing mice were treated with zoledronate (ZA), an inhibitor of osteoclast activity (5 μg/kg, every 2 days, s.c.) or anti‐RANKL neutralizing antibody, an inhibitor of osteoclast formation (250 μg per mouse, every 3 days, i.p.). Effects on bone morphometry, as well as on muscle size and strength were assessed. OC growth was accompanied by elevated circulating RANKL (Control: 86 pg/mL; ES‐2: 360 pg/mL) and by marked loss of body weight (−20% versus Control, *p* < 0.01), which remained substantially unchanged after administration of either ZA or anti‐RANKL treatments. None of the drugs affected tumor size (ZA: −4%; anti‐RANKL: −3% versus ES‐2). Conversely, ZA completely restored the bone derangements in the ES‐2 bearers (BV/TV: −50%; Tb.Th: −20%; Tb.Sp: +18%; Tb.N: −25% versus Control, *p* < 0.01), and OC‐induced skeletal muscle atrophy (quadriceps: −31% versus Control, *p* < 0.01) and muscle weakness (−30% versus Control, *p* < 0.01) were significantly improved by both ZA (quadriceps mass: +12%; muscle strength: +12%, *p* < 0.01) and anti‐RANKL (quadriceps: +17%; muscle strength: +20%, *p* < 0.01). Our observations suggest that abnormalities of the muscle/bone cross‐talk play a role in the pathogenesis of cancer cachexia. Specifically, we showed that blockade of cancer‐induced bone resorption, even in the absence of bone metastases, concurrently results in improvement of muscle mass and function. Further investigations are warranted to clarify whether anti‐resorptive treatments could contribute to reduce chemotherapy‐induced musculoskeletal toxicities in combination with routinely prescribed anti‐cancer drugs.


**Matt Prideaux, PhD**


Indiana University, Indianapolis, IN, USA

Relevant Session: Role of Muscle and Bone Factors in Energetics and Metabolism

DOI: 10.002/jbm4.10313


## Taurine Is a Primary Osteocyte‐Synthesized Metabolite That Protects Against Oxidative Stress‐Induced Cell Death and Regulates Sclerostin Expression

### Matthew Prideaux,^1^ Yukiko Kitase,^1^ Morris Kimble,^1^ Thomas M. O'Connell,^1,2^ and Lynda F Bonewald^1,3^


#### 
^1^Indiana Center for Musculoskeletal Health, Indiana University, Indianapolis, IN, USA; ^2^Department of Otolaryngology, Indiana University, Indianapolis, IN, USA; ^3^Department of Orthopedic Surgery, Indiana University, Indianapolis, IN, USA

Osteocytes are long‐lived cells, which are essential for bone remodeling by regulating osteoblasts and osteoclasts. To examine the metabolic processes associated with osteocyte function, we performed an untargeted NMR‐based analysis using primary osteoblasts/osteocytes. Primary cells were isolated from the long bones of 7‐day‐old mice and differentiated into osteoblasts or osteocytes and the culture media and cell lysate were analyzed. One of the most abundant metabolites produced by the primary cells was taurine, a metabolite normally secreted by the liver. Interestingly, no taurine was detected in the culture media, suggesting that endogenous taurine produced by these cells is retained within the cells. Furthermore, taurine levels were significantly higher in the day 28 osteocyte‐like cells compared with the day 7 osteoblastic cells (30%, *p* = <0.05). To further investigate taurine production by osteocytes, we utilized the IDG‐SW3 cell line and found that taurine was also synthesized by the cells but not secreted into the media. Taurine levels increased two‐fold from day 4 to day 18 of differentiation and remained elevated at day 28 (*p* = <0.001). RNA‐Seq and RT‐PCR analysis showed that cysteine dioxygenase (Cdo1) a key enzyme for the taurine biosynthesis was upregulated more than two‐fold during differentiation (*p* = <0.001). To examine the potential role of taurine in osteocytes, we treated mature IDG‐SW3 cells with exogenous taurine (1 to 50 mM). Taurine dose‐dependently inhibited Sost mRNA and sclerostin protein expression up to 70% (*p* = <0.01) and Dkk1 mRNA up to 50% (*p* = <0.05). As taurine is known to activate glycine receptors, we also tested the effects of glycine on the cells. Glycine (1 to 50 mM) dose‐dependently inhibited Sost mRNA and sclerostin protein levels greater than 70% (*p* = <0.001). Additionally, we used the MLO‐Y4 cell line to examine the effects of taurine on cell survival. Taurine at 1 to 50 mM reduced H2O2‐induced cell death by 30% to 60% (*p* < 0.001) and similar effects were found with glycine. Therefore, we have shown that osteocytes synthesize taurine, which may have beneficial effects by promoting cell viability and bone formation. As the effects of taurine are duplicated by glycine, the glycine receptor may be a promising target for the effects of these amino acids on osteocytes.


**Jamie Renbarger, PhD**


Indiana University, Indianapolis, IN, USA

DOI: 10.002/jbm4.10314


## Beneficial Effects of Exercise on Adolescent and Young Adult Cancer Survivors

### Jamie Renbarger

#### Indiana University, Indianapolis, IN, USA

Disease outcomes for adolescents and young adults (AYA: ages 15 to 39 years) with cancer have improved significantly over the past four decades to a current 5‐year survival rate of more than 80%. AYA survivors have decades of life years after completion of cancer treatment and are at very high risk for developing chronic medical and mental health conditions. Moreover, after cancer treatment, adult survivors of pediatric cancers are significantly less active than their sibling controls, and AYA survivors often have impaired balance. It is well established in the general adult population that exercise not only improves fitness but also prevents and attenuates several health conditions and improves health outcomes. Improvement in these health outcomes is associated with changes in key plasma biomarkers of cardiovascular fitness. There is some evidence that structured exercise (prescriptions, programming, and participation) improves strength, fatigue, and maximal aerobic capacity in older adult cancer patients and survivors; however, this structured approach has not been evaluated in AYA cancer survivors. Furthermore, the impact of this approach on long‐term health and healthcare costs is unknown. We are determining the effects of structured exercise in the AYA cancer survivor population on fitness, plasma biomarkers, daily physical activity, fatigue, mental health outcomes, and QOL. We are also evaluating the short‐term benefits of a one‐on‐one community‐based, structured exercise approach that pairs AYA cancer survivors with young adult Clinical Cancer Exercise Specialists. Our early results will be discussed. Our goal is to fill a critical gap by providing the evidence base needed to drive innovative, individualized cancer exercise with an initial focus on AYA survivors as an underserved population with decades of life years after cancer treatment.


**Joseph Rupert, PhD (c)**


Indiana University, Indianapolis, IN, USA

Relevant Session: Biomechanical Relationships Between Muscle and Bone

DOI: 10.002/jbm4.10315


## IL‐6 Trans‐Signaling among Tumor, Muscle and Fat Mediates Pancreatic Cancer Cachexia

### Joseph Rupert,^1^ Andrea Bonetto,^1,4,5^ Ashok Narasimhan,^2^ Leonidas Koniaris,^2,4,5^ and Teresa Zimmers^1,2,3,4,5,6^


#### Departments of ^1^Biochemistry and Molecular Biology, ^2^Surgery, ^3^Anatomy and Cell Biology, and ^4^Otolaryngology Head & Neck Surgery, Indiana University School of Medicine; ^5^IUPUI Center for Cachexia Research, Innovation & Therapy (CCRIT); ^6^IU Simon Cancer Center, Indianapolis, IN, USA

Cachexia, the involuntary loss of fat, muscle, and bone, is associated with pancreatic ductal adenocarcinoma (PDAC) and contributes to the 5‐year mortality of >91%. Interleukin‐6 (IL‐6) is increased in the blood of patients with PDAC and correlates with increased cachexia and reduced survival. IL‐6 has been well documented, yet IL‐6 signaling mechanisms between tissues remain elusive. IL‐6 activates signal transduction by binding with the membrane‐bound IL‐6rα (classical signaling) or the soluble IL‐6rα (sIL‐6rα; trans‐signaling), produced from shedding of the membrane receptor. Here, we investigate the mechanisms of IL‐6 signaling between tumor, fat, and muscle and the subsequent effects on cachexia using isolated tumor cells from the LSL‐KrasG12D:LSL‐Trp53R172H:Pdx1‐Cre (KPC) genetic mouse model of PDAC. We used CRISPR/Cas9 editing to delete IL‐6 expression in KPC tumor cells (KPC IL‐6^KO^) and orthotopically injected mice with KPC or KPC IL‐6^KO^ cells.

KPC tumors robustly expressed IL‐6 in both tumor and stromal cells, while also causing increased plasma IL‐6 and sIL‐6Ra protein levels in mice. KPC tumor mice had increased IL‐6 gene and protein expression in adipose tissue but not muscle. Interestingly, while KPC tumor mice had increased IL‐6Ra gene but not protein expression in muscle, adipose tissue IL‐6Ra gene expression was unchanged while concomitant accumulation of sIL‐6Ra protein (55 kDa) was measured. Furthermore, increased plasma glycerol and fatty acids, augmented fat loss, reduced muscle mass, and increased myosteatosis were observed in KPC tumor mice. Increased cachexia severity was largely due to tumor‐derived IL‐6, since KPC IL‐6^KO^ tumor mice had attenuated cachexia and increased survival without changes in tumor size or the use of an anti‐tumor therapy. Thus, our data implicate a feed‐forward signaling loop in PDAC cachexia, sparked by tumor‐derived IL‐6, resulting in skeletal muscle sIL‐6Ra production, which augments adipose tissue lipolysis via IL‐6 trans‐signaling and promotes lipotoxicity‐induced skeletal muscle wasting in PDAC.


**Alberto Smargiassi, PhD**


Indiana University, Indianapolis, IN, USA

Relevant Session: Role of Soluble Factors in Muscle‐Bone Interactions

DOI: 10.002/jbm4.10316


## The Effect of Contracted Muscle‐Derived Metabolite L‐β‐Aminoisobutyric Acid (L‐Baiba) and Fluid Flow Shear Stress (FFSS) Alone and in Combination on the Osteocyte Cell‐Line MLO‐Y4

### Alberto Smargiassi, Yukiko Kitase, and Lynda Bonewald

#### Indiana Center for Musculoskeletal Health, Indianapolis, IN, USA

L‐BAIBA is a metabolite produced by contracting muscle. Previously we demonstrated that L‐BAIBA attenuated both bone and muscle loss in vivo by hindlimb unloading and in vitro showed the metabolite mediates protective signals in osteocytes through the MAS Related G Protein Receptor Family Member type D, MRGPRD (Kitase et al., Cell Reports, 2018). Recently we have found that L‐BAIBA has no effect on bone formation but enhances the effects of anabolic loading on the bone (ASBMR, 2019). To identify the molecular mechanism responsible for these in vivo results, we utilized the MLO‐Y4 cells as an osteocyte model subjected to loading of 16 dynes/cm^2^ fluid flow shear stress (FFSS) for 2, 6, and 24 hours. In addition to MRGPRD expression we selected, based on previous Rna‐Seq studies conducted on osteocyte isolated from mechanically loaded tibias, markers of osteocytes such as Dmp1 and members of the b‐catenin signaling pathway, Wnt1 and Wnt10b to be examined. Osteocytes have also been shown to express muscle‐related genes such as the Myosin Heavy Chains, Mhy7, Mhy9, and Mhy11. As these genes are regulated by loading in muscle, we sought to determine if they are regulated by loading in osteocytes. By qPCR, MRGPRD gene expression was increased by either FFSS or L‐BAIBA, but the combination was not additive or synergistic. The bone‐related genes Dmp1 and Wnt1 were only regulated by FFSS but not L‐BAIBA. Wnt10b was increased when exposed to FFSS or L‐BAIBA, but again there were no additive or synergistic effects with the combination. Mhy7 expression was only increased with FFSS. Myh11 expression showed a biphasic trend with FFSS initially suppressing its expression at 2‐hour time point but reversing the effect at the 6‐hour time point. Mhy9 was increased with both FFSS or L‐BAIBA but again no interaction of the two stimuli. MRGPRD expression suggests that a sequential effect of loading and BAIBA may be responsible for the in vivo results. Loading may increase receptor expression to provide a more potent effect of BAIBA on some but not all osteogenic genes and potentially myosin genes that may play a role in activating the osteocyte cytoskeleton.


**Hyunggwi Song, PhD**


University of Illinois at Urbana‐Champaign, Urbana, IL, USA

Relevant Session: Biomechanical Relationships Between Muscle and Bone

DOI: 10.002/jbm4.10317


## Effect of Exercise on Three‐Dimensional Subchondral Bone Properties in Sheep

### Hyunggwi Song,^1^ John D Polk,^2^ and Mariana E Kersh^1,3^


#### 
^1^Department of Mechanical Science and Engineering, ^2^Department of Anthropology, ^3^Beckman Institute for Advanced Science and Technology, University of Illinois at Urbana‐Champaign, Urbana, IL, USA

Changes in subchondral properties enable bone to be stiff enough to support loads and compliant enough to avoid high local stresses (Currey, 2002). While many studies have investigated the effects of exercise on bone, it is unclear whether there are local differences across the joint surface associated with joint posture and increased loading. The goal of this study was to identify the effects of exercise on subchondral bone thickness and density throughout the articular surface. Following IACUC approval, 30 healthy juvenile (age 60 days) sheep were divided into three groups: (1) flat treadmill exercise (*n* = 11), (2) incline treadmill exercise (15% grade; *n* = 11), and (3) control (*n* = 8). Exercise occurred twice daily at 1.12 m/s for 20 min/bout for 60 days. Kinematic data were measured with a motion capture system at 160 Hz (Qualisys). After euthanizing, the hindlimbs were harvested and microCT scans of femora were acquired with a resolution of 50 μm (Siemens Inveon). Hydroxyapatite phantoms were used to convert Hounsfield Units to apparent density (CIRS). The femora were segmented semi‐automatically from the CT images (Amira 6.4) and the subchondral surface was reconstructed in 3D. Thickness and density in the subchondral bone surface was measured using Matlab and BoneJ (ImageJ). Exercised sheep experienced 3600 loading cycles more per day than the control. The thickness of flat and incline groups on the anterior region was 31% and 27% greater (*p* < 0.05) than the control group, respectively, while only the density of the flat group in the medial‐anterior and the lateral‐posterior regions was 23% greater than that of control group (*p* < 0.05). Between flat and incline groups, flat exercise resulted in a 27% and 19% greater thickness and density, respectively, than the incline group, but these increases were not isolated to a specific region (*p* < 0.05). Flat exercise resulted in 19% and 21% larger area of significantly increased thickness and density changes than incline group, suggesting that the inclined group might be loaded with a smaller area on the subchondral surface, resulting in smaller differences. Our results suggest that increased posture may result in decreased tibio‐femoral contact and therefore localized adaptation in thickness and density.


**Lester Smith, PhD**


Indiana University, Indianapolis, IN, USA

Relevant Session: Muscle‐Bone Interactions in Orthopedics

DOI: 10.002/jbm4.10318


## Optimizing IDG‐SW3 Spheroids for Bioprintability

### Jane Sterner^1,2^ and Lester Smith^1,2^


#### 
^1^Department of Radiology and Imaging Sciences, Indiana University School of Medicine, Indianapolis, IN, USA; ^2^3D Bioprinting Core, Indiana University School of Medicine, Indianapolis, IN, USA

Cell‐dense microtissue spheroids made from IDG‐SW3 cells are useful as the 3D biofabrication printing medium to make bone tissue constructs. The Kenzan 3D bioprinting method produces tissue by impaling ~400 to 600 micron spheroids onto an array of needles whereby the spheroids fuse to form larger tissues. Since spheroids must be mechanically robust enough to withstand needle placement and IDG‐SW3 cells are highly sensitive to culture conditions, we investigated the culture parameters critical for generating printable spheroids. We compared the influence of centrifugation, cells per well, and culture medium on the generation of bioprintable spheroids. Spheroids were formed by seeding PN 22 IDG‐SW3 cells in ultra‐low adhesion (ULA) 96‐well round‐bottom plates and incubating them at 33°C and 5% CO_2_ for 48 hours. Combinations of the following conditions were compared:Cell number: 30,000 or 40,000 cells per well.Culture medium conditions: Control medium (Alpha MEM, 10% FBS, and 1% antibiotic) or proliferation medium (control medium plus 50 U/mL interferon gamma [IFN‐γ]).Centrifugation conditions: No centrifugation or centrifugation at room temperature and 500 rpm for 5 minutes.


Only incubation of 40,000 cells per well in control medium resulted in printable spheroids, while centrifugation had no influence on printability. Each cell type used in spheroids for bioprinting must be optimized to withstand the printing process. Therefore, ongoing studies are investigating the influence of incubator (ie, CO_2_ and temperature) conditions and which and how spheroid mechanical properties lead to bioprintability.


**Sukanya Suresh, PhD**


Indiana University, Indianapolis, IN, USA

DOI: 10.002/jbm4.10319


## Differential Effects of High‐Fat Diet in Bone, Muscle, and Metabolic Parameters in an Athymic Nude Mice Model

### Sukanya Suresh, Laura E Wright, Sutha John, Yun She, Sreemala Murthy, Trupti Trivedi, Gabriel Pagnotti, Khalid Mohammad, and Theresa Ae

#### Department of Medicine, Division of Endocrinology, Indiana University School of Medicine, Indianapolis, IN, USA

Athymic nude mice are immunodeficient, making them suitable models for tumor xenograft studies. Obesity is associated with increased risk of cancer development, progression, and metastasis. To understand specific effects of excess body fat on tumor development, it is essential to develop immunodeficient animal models that respond to high‐fat diet (HFD)–induced metabolic changes and acquire features similar to the development of obesity in humans. Here, we characterized the impact of HFD on bone, muscle, fat, and other metabolic parameters in 9‐week‐old female athymic nude mice fed a control diet (10% fat) or HFD (60% fat) for 18 weeks. A subset (*n* = 5/group) of mice were analyzed after 4 weeks. At 4 weeks, there was no difference in body weight, fat, or lean mass among the diet groups. Forelimb grip strength was not different, and ex vivo contractility measurement of extensor digitorum longus (EDL) muscle showed increased muscle specific force in HFD‐fed mice (*p* < 0.05).The HFD fed mice had 32% reduced trabecular bone volume fraction (BV/TV) compared with the control diet (*p* < 0.05) and significant induction in marrow adiposity (*p* < 0.05). At 18 weeks, there were no significant differences in the forelimb grip strength or in EDL contractility among the diet groups. Trabecular bone volume fraction increased by 50% in the HFD‐fed mice relative to control diet–fed mice (*p* < 0.05), but there was no difference in marrow adiposity with long‐term HFD feeding. Mechanical testing of the bones did not show any difference in ultimate force among the diet groups at either time points. We observed that in older athymic nude mice (>20 weeks of age), the HFD‐fed group consistently showed lower body weight in comparison with mice on control diet (*p* < 0.05, *p* < 0.01), but there was no difference in fat mass or lean mass among the different diet groups. Analysis of glucose tolerance and insulin sensitivity showed similar trends among control and HFD‐diet groups. Thus athymic nude mice are resistant to HFD‐induced increase in body weight and fat mass. They also do not exhibit impaired glucose tolerance or insulin sensitivity with long‐term high‐fat diet feeding.


**Vidyani Suryadevara, PhD**


Indiana University, Indianapolis, IN, USA

DOI: 10.002/jbm4.10320


## The Unexpected and Distinctive Roles of the Muscle Ring‐Finger (MuRF) Family of Ubiquitin Ligases (MuRF1, MuRF2, and MuRF3) on Bone Microarchitecture

### Vidyani Suryadevara,^1^ Amy Sato,^2^ Katherine Powell,^3^ Joseph Wallace,^3^ Matthew Allen,^2^ Teresita Bellido,^2^ and Monte S Willis^1,4,5^


#### 
^1^Departments of Pathology & Laboratory Medicine, ^2^Anatomy and Cell Biology, ^3^School of Engineering and Technology, Department of Orthopedic Surgery, ^4^Indiana Center for Musculoskeletal Health, ^5^Krannert Institute of Cardiology and Division of Cardiology, Department of Internal Medicine, Indiana University School of Medicine, Indianapolis, IN, USA

People with osteoporosis have an increased risk of fractures and abnormal bone quality due to reduced bone strength. Over the age of 50 years, one in two women and one in four men are prone to bone loss. The ubiquitin‐proteasome system (UPS) is the principal regulator of protein catabolism in the mammalian cytosol and nucleus and plays critical roles in cell biology in all cells of the body. The UPS is involved in osteoblast differentiation and bone formation and osteoclast function (resorption). Ubiquitin ligases (E3s) drive the specificity of protein ubiquitination. The Muscle Ring Finger (MuRF) family of E3s are found exclusively in striated muscle. While recent reports identified MuRF1 mRNA in osteoblasts, the role of MuRF proteins on bone mechanical properties is not known. Since evidence for the muscle‐bone communication during development, sarcopenia, and osteoporosis continues to grow, we investigated the effects of MuRF1, MuRF2, MuRF3 deletion in bone microarchitecture and mechanical properties. Adult MuRF1−/−, MuRF2−/−, and MuRF3−/− mice, along with sibling heterozygote and wild‐type mice, were analyzed at ~3 to 4 months of age, a time point where no cardiac or skeletal muscle phenotype is present. Both femurs from each mouse were dissected, cleaned of soft tissue and wrapped in saline‐soaked gauze, immediately stored at –20°C, and subsequently assessed by microCT (SkyScan 1172) for bone microarchitecture in the distal femur metaphysis (1 mm) and mid‐diaphysis region. Femurs were then challenged to the three‐point bending test (TestResources Inc) to assess the material and structural properties of the bone. We identified significant increases in cortical thickness in the MuRF1−/− and MuRF1+/− compared with wild‐type mice (0.24 ± 0.01, 0.23 ± 0.01, 0.22 ± 0.01, respectively, *p* < 0.05). MuRF1−/− cortical area was significantly higher than WT mice (0.99 ± 0.08, 0.9 ± 0.08, *p* < 0.05). While MuRF2 and MuRF3 depletion did not affect cortical bone parameters, the MuRF2 +/− had altered mechanical properties exhibited by an increase in toughness compared with WT controls (35.58 ± 21.73, 21.08 ± 6.3, respectively, *p* < 0.05). Together these studies illustrate the role of MuRF1 and MuRF2 depletion in protecting against bone loss, which may represent therapeutic targets in age‐related bone loss and osteoporosis paralleling their potential roles in sarcopenia.


**Josephine Tauer, PhD**



**Early Investigator Awardee*


Shriners Hospital for Children, Montreal, Canada

DOI: 10.002/jbm4.10321


## Osteocalcin Deficiency in *Col1a1_Jrt/+_* Mice, a Model of Severe Dominant Osteogenesis Imperfecta, Rescues Metabolic Phenotype

### JT Tauer and SV Komarova

#### Shriners Hospital for Children and Faculty of Dentistry, McGill University, Montreal, Canada

Osteocalcin, an osteoblast‐derived hormone, is among the most abundant proteins in bone and is involved in the regulation of whole‐body metabolism, muscle adaptation, and reproduction. High bone turnover and low bone mass are clinical hallmarks of Osteogenesis Imperfecta (OI), a bone disease mainly caused by mutations in the Collagen‐I gene. Recently, we have shown that growing mice with a severe dominant form of OI, *Col1a1Jrt/+* mice, displayed significantly elevated serum levels of undercarboxylated osteocalcin (uOCN) along with an altered glucose/insulin metabolism and energy expenditure. Further, these mice are protected against high‐fat diet–induced obesity but not insulin resistance. To further confirm the role of uOCN, we crossed *Col1a1Jrt/+* mice (OI) with mice lacking one or both osteocalcin genes (OCN+/−, OCN−/−) to generate OI/OCN mice. At 4 and 8 weeks of age, wild‐type (WT/WT) and OI/OCN mice were phenotypically characterized and random glucose (RG) measurements along with glucose tolerance (GT) test were performed. To generate OI/OCN mice, fertile OCN+/− and OI mice were used. Within the first generation, about 27% of generated mice were WT/OCN+/−, 24% OI/WT, 26% OI/OCN+/−, 22% WT/WT, and 1% were found dead. For further breeding, OI/OCN+/− mice were used and gave birth to pups with a genetic distribution of about 18% OI/OCN+/− or WT/OCN+/−*,* 21% OI/OCN−/−, 12% WT/OCN−/−, 6% OI/WT, and 12% were found dead. Compared with WT/WT, 4‐week‐old mice harboring the genotype OI/WT, OI/OCN+/−*,* and OI/OCN−/− were smaller in size and up to 20% lower in body mass, which declined to about 15% difference at 8 weeks of age. At 4 weeks of age, OI/OCN+/− and OI/OCN−/− mice exhibited lower RG levels than WT/WT littermates (*p* < 0.05). However, only OI/OCN+/− mice revealed improved GT (*p* < 0.01), while OI/OCN−/− mice did not differ from WT/WT. At 8 weeks of age, no significant differences in RG or GT were found in OI/OCN+/− or OI/OCN−/− mice compared with WT/WT. Here we show for the first time that knock‐out of both osteocalcin genes restored glucose tolerance to WT levels in mice with severe dominant OI, strongly supporting the causative role of osteocalcin driving alteration in glucose/insulin metabolism in OI mice.


**Jingjuan (Candice) Chen, BS**


Purdue University, West Lafayette, IN, USA

DOI: 10.002/jbm4.10325


## Function of Two Novel Methyltransferases That Are Uniquely Expressed in Slow and Fast Myofibers in the Skeletal Muscle

### Chao Wang,^1^ Jingjuan Chen,^1^ Bin Zhang,^2^ Anna C Ratliff,^3^ Justine Arrington,^3^ Hannah E Horton,^4^ Yaohui Nie,^1^ Feng Yue,^1^ Yan Xiong,^2^ Keping Hu,^2^ Wen Jin,^2^ W Andy Tao,^5,6^ Christine A Hrycyna,^3,6^ Xiaobo Sun,^2^ and Shihuan Kuang^1,6^


#### 
^1^Department of Animal Sciences, Purdue University, West Lafayette, IN, USA; ^2^Institute of Medicinal Plant Development, Peking Union Medical College and Chinese Academy of Medical Sciences, Beijing, China; ^3^Department of Chemistry, Purdue University, West Lafayette, IN, USA; ^4^Department of Biological Sciences, Purdue University, West Lafayette, IN, USA; ^5^Department of Biochemistry, Purdue University, West Lafayette, IN, USA; ^6^Center for Cancer Research, Purdue University, West Lafayette, IN, USA

Contractile and metabolic functions of skeletal muscles are determined by their heterogeneous myofiber composition. Most mammalian skeletal muscles contain a unique combination of four myofiber types (I, IIa, IIx, IIb) that are sequentially faster in contraction speed and larger in size but lower in mitochondria content and oxidative capacity. What determines the distinct properties of the four types of myofibers is unclear. Here we identify two methyltransferase‐like 21 family members (Mettl21c and Mettl21e) as novel regulators of myofiber heterogeneity. *Mettl21c* and *Mettl21e* genes are located proximately on the same chromosome in mouse genome but are differentially expressed in type I and IIb myofibers, respectively. *Mettl21c* or *Mettl21e* knockout has no effect on muscle development and myofiber patterning, suggesting these methyltransferases mainly function in postnatal muscles. Indeed, the *Mettl21c* knockouts exhibit muscle weakness and impaired mitochondrial function, whereas the *Mettl21e* knockouts have smaller IIb‐myofibers and accelerated denervation‐induced muscle wasting. Tandem affinity purification and mass spectrometry reveal association of Mettl21c with heat shock proteins (HSP) and Mettl21e with 26S proteasome associated proteins. Further analysis indicates that Mettl21c mediates the lysine‐561 trimethylation of Hspa8/Hsp70, a chaperone required for protein import into mitochondria. In contrast, Mettl21e methylates Vcp/p97, a major regulator of ubiquitin‐proteasome system. Mettl21c knockout reduces the trimethylation level and stability of Hspa8, whereas Mettl21e KO increases 26S protease activity. Hence, Mettl21c and Mettl21e function divergently to regulate mitochondrial function and protein degradation in subsets of myofibers. Our results exemplify multiple physiological functions of non‐histone methylation‐mediated by Mettl21 family proteins.


**David Waning, PhD**


Penn State College of Medicine, Hershey, PA, USA

DOI: 10.002/jbm4.10326


## Zoledronic Acid Improves Muscle Function in Mice Treated with Chemotherapy

### Brian A Hain,^1^ Baptiste Jude,^1^ Haifang Xu,^1^ Dallas Smuin,^2^ Edward J Fox,^2^ and David L Waning^1,3^


#### 
^1^Penn State College of Medicine, Department of Cellular & Molecular Physiology, Hershey, PA, USA; ^2^Penn State College of Medicine, Department of Orthopaedics and Rehabilitation, Hershey, PA, USA; ^3^Penn State Cancer Institute, Hershey, PA, USA

Carboplatin, a platinum‐based chemotherapy, causes an array of side effects including loss of bone mass, muscle atrophy, and muscle weakness. The goal of this study was to determine if bone‐muscle cross‐talk was mediating muscle weakness in mice treated with carboplatin. In order to determine the role that bone plays in carboplatin‐induced muscle weakness, female Balb/C mice were treated with carboplatin and the anti‐resorptive bisphosphonate zoledronic acid (ZA). Seven days later, mice were euthanized and whole muscle contractility was measured using the extensor digitorum longus (EDL) muscle. Specific force was significantly lower in carboplatin‐treated mice, which was prevented by the addition of ZA. However, ZA did not rescue the loss of muscle mass or reduction in myofiber cross‐sectional area (CSA), and expression of the muscle‐specific ubiquitin ligase MuRF1 was higher in muscle from mice treated with carboplatin. Carboplatin had severe effects on bone with reduced bone volume fraction (BV/TV), trabecular thickness (Tb.Th), and number (Tb.N), and increases trabecular separation (Tb.Sp) in the tibia and femur. ZA prevented the carboplatin‐induced loss of bone. To establish the direct role of carboplatin on skeletal muscle function in the absence of bone resorption, mice were treated with carboplatin for 1 or 3 days. At these earlier time points, no changes were observed in EDL muscle function, muscle mass, or myofiber CSA, and no changes in bone were observed by microCT. We confirmed carboplatin presence in the EDL muscle by platinum analysis using inductively coupled plasma mass spectrometry (ICP‐MS).

Platinum concentration was at a clinically relevant level of 1.0 ng/mg in muscle tissue 24 hours after injection with a steady decline to (0.6 ng/mg) by 10 days. Our data suggest that carboplatin‐induced muscle weakness is caused by bone resorption, while muscle atrophy is caused by direct effects on muscle. These findings are clinically relevant to prevent muscle weakness in cancer patients and can be readily addressed using available drugs such as ZA.


**Stuart Warden, PT, PhD**


Indiana University, School of Health & Human Sciences, Indianapolis, IN, USA

DOI: 10.002/jbm4.10327


## Heterogeneous Spatial and Strength Adaptation of the Proximal Femur to Physical Activity: A Within‐Subject Controlled Study

### Stuart Warden,^1^ Julio Carballido‐Gamio,^2^ Joyce Keyak,^3^ Alyssa Weatherholt,^4^ Thomas Lang,^5^ Mariana Kersh,^6^ and Robyn Fuchs^7^


#### 
^1^Indiana University, Indianapolis, IN, USA; ^2^University of Colorado Anschutz Medical Campus, Aurora, CO, USA; ^3^University of California Irvine, Irvine, CA, USA; ^4^University of Southern Indiana, Evansville, IN, USA; ^5^University of California San Francisco, San Francisco, CA, USA; ^6^University of Illinois at Urbana‐Champaign, Urbana‐Champaign, IL, USA

Physical activity (PA) enhances proximal femur bone mass, as assessed using projectional imaging techniques. However, these techniques average data over large volumes to obscure spatially heterogeneous adaptations. The current study used quantitative computed tomography, statistical parameter mapping, and subject‐specific finite element (FE) modeling to explore spatial adaptation of the proximal femur to PA. In particular, we were interested in adaptation occurring at the superolateral femoral neck and improving strength to loading in a sideways fall direction. High/long jump athletes (*n* = 16) and baseball pitchers (*n* = 16) were utilized as within‐subject controlled models as they preferentially load their takeoff leg and leg contralateral to their throwing arm, respectively. Controls (*n* = 15) were included but did not show any dominant‐to‐nondominant (D‐to‐ND) leg differences. Jumping athletes showed some D‐to‐ND leg differences but less than pitchers. Pitchers had 5.8% (95% CI, 3.9% to 7.6%) D‐to‐ND leg differences in total hip volumetric bone mineral density (vBMD), with increased vBMD in the cortical compartment of the femoral neck and trochanteric cortical and trabecular compartments. Voxel‐based morphometry analyses and cortical bone mapping showed pitchers had D‐to‐ND leg differences within the regions of the primary compressive trabeculae, inferior femoral neck, and greater trochanter but not the superolateral femoral neck. FE modeling revealed pitchers had 4.1% (95% CI, 1.4% to 6.7%) D‐to‐ND leg differences in strength to single‐leg stance loading but no differences to loading in a sideways fall direction. These data indicate axially directed loading induces proximal femur adaptation in regions associated with weight bearing and muscle contractile forces and increases strength to single‐leg stance loading. The lack of benefit at the superolateral femoral neck and to sideways fall loading raises questions as to whether PA programs that increase proximal femur bone mass improve bone strength to common injurious loading during aging.


**Ashley Weaver, PhD, MS**


Wake Forest School of Medicine, Winston‐Salem, NC, USA

DOI: 10.002/jbm4.10328


## Relationship of Sarcopenia and Osteosarcopenia With Age, Sex, Injury Severity, and Fracture in Seriously Injured Motor Vehicle Crash Occupants

### Ashley A Weaver,^1,2^ Casey Costa,^1,2^ Josh Tan,^2^ Lisa Maez,^2^ Xin Ye,^1,2^ Joel D Stitzel,^1,2^ and Leon Lenchik^2^


#### 
^1^Virginia Tech‐Wake Forest University School of Biomedical Engineering and Sciences; ^2^Wake Forest University School of Medicine

As adults over age 60 years comprise 27% of licensed US drivers, it is important to understand the effects of sarcopenia and osteosarcopenia in motor vehicle crash trauma. This study examined the prevalence of sarcopenia (low muscle mass) and osteosarcopenia (low bone density and muscle mass) in older MVC occupants and the relationship of these conditions with age, sex, injury severity, and fracture. Skeletal muscle cross‐sectional area (CSA) and lumbar BMD was measured from abdominal computed tomography (CT) scans of 61 seriously injured MVC occupants over age 50 years in the Crash Injury Research and Engineering Network (CIREN) database. All visualized muscles at the L_3_ vertebral level were segmented by thresholding muscle from −29 to +150 Hounsfield Units to compute the muscle CSA in cm^2^. Sarcopenia was indicated by the skeletal muscle index, calculated as SMI = muscle CSA/(occupant height)^2^, with thresholds SMI < 52.4 cm^2^/m^2^ for men and SMI < 38.5 cm^2^/m^2^ for women. Lumbar BMD less than 145 mg/cm^3^ indicated osteopenia. Descriptive statistics, *t* tests, and chi‐square tests were performed. The prevalence was 43% for sarcopenia, 25% for osteopenia, and 15% for osteosarcopenia in the MVC occupants. Age, sex, crash speed, and crash mode were similar between sarcopenic and non‐sarcopenic occupants. On average, SMI was 17.1 cm^2^/m^2^ lower in sarcopenic males and 14.7 cm^2^/m^2^ lower in sarcopenic females compared with their non‐sarcopenic counterparts (*p* < 0.001). Lumbar BMD was an average 20.6 mg/cm^3^ lower in sarcopenic occupants (*p* = 0.049). Osteopenia was present in 35% of sarcopenic occupants compared with 17% of non‐sarcopenic occupants. The Injury Severity Score was higher in those with only sarcopenia (mean ± standard error: 22.4 ± 2.3), followed by those with osteosarcopenia (17.9 ± 2.4) and only osteopenia (12.8 ± 1.5). More total fractures were observed in occupants with sarcopenia alone (7.6 ± 1.5) or osteosarcopenia (7.0 ± 2.1) compared with non‐sarcopenic occupants with osteopenia (4.0 ± 2.5). CT assessment of sarcopenia and osteosarcopenia in MVC occupants may explain contributing factors to the causation and mechanisms of injury, and identify patients with a high risk of poor outcomes who may benefit from additional rehabilitation to preserve function.


**Monte S. Willis, MD, PhD, MBA**


Indiana University, Indianapolis, IN, USA

DOI: 10.002/jbm4.10329


## Alzheimer's Disease and the Co‐Morbidities Related to the Musculoskeletal System: Lessons from Patients and Animals

### Monte S Willis

#### Deputy Director, Indiana Center for Musculoskeletal Health Vice Chair of Research, Department of Pathology & Laboratory Medicine, Professor, Krannert Institute of Cardiology, Department of Medicine, Indiana University School of Medicine, Indianapolis, IN, USA

Alzheimer's disease represents a significant threat to the health of the US population. The most common causes of morbidity and mortality in AD patients are associated with muscle function (swallowing), bone and muscle strength (hip fractures), and heart failure severity used in advanced dementia prognostic tools. AD patients experience an underlying sarcopenia and decreased strength increasingly present with AD disease progression after adjusting for multiple covariates and differentially affected women more than men. This reduced muscle strength did not parallel the rate of decline in global cognitive function tests, with underlying mechanisms currently unknown. New conceptual frameworks are needed to address this issue to allow a better understanding of the complexity of aging diseases so that therapeutics targeting multiple systems can be developed. The association with AD and heart failure patients have linked the presence of amyloid beta (Aβ) protein aggregates in the hearts of patients with a primary diagnosis of AD. An increase in cardiac Aβ protein and the degree of diastolic dysfunction before death were correlated, indicating a possible mechanistic link between Aβ protein and cardiac dysfunction. Subsequent echocardiographic studies of AD patients and age‐matched controls with AD‐mutations (ie, PSEN1, PSEN2, APP, and APOE genes) and ECG analysis have found multiple echocardiographic abnormalities, including diastolic dysfunction and low‐voltage QRS complexes and cardiac hypertrophy by electrocardiography (ECG), together illustrating a link between subclinical cardiac disease and Aβ amyloid deposition in AD patients. These findings have led to a concerted effort to phenotype the cardiac and musculoskeletal system in both animal and human models of AD disease in our research group. We focus on both clinically relevant and diverse animal models for muscle, bone, and cardiac alterations driven by multiple known mechanisms, including mutations in Aβ and the microtubule‐associated protein tau (MAPT) and the role of endogenous tau protein and microtubule function throughout the body. We've identified a spectrum of pathologic changes in cardiac, muscle, and bone is seen in almost all AD models we have investigated to date, with deficits getting worse with age. These unexpected findings will allow us to determine both the underlying mechanisms and to test therapeutic interventions for both brain and the musculoskeletal system disease in AD patients. Our goal is to improve the AD patient's quality of life as it relates to the musculoskeletal system while attenuating brain disease as part of a systemic protein quality control disease throughout the body. These will allow us to translate our limited knowledge and fledging understanding of AD patients’ musculoskeletal disease, which is not a critical concern clinically today, given our limited knowledge and fledging understanding of its expression.


**Christopher Wolff**


University of Florida, Gainesville, FL, USA

Relevant Session: Biomechanical Relationships Between Muscle and Bone

DOI: 10.002/jbm4.10330


## Age‐Related Loss of Circadian Robustness in Skeletal Muscle

### Christopher A Wolff, Miguel Gutierrez‐Monreal, Collin M Douglas, Lance A Riley, Denise Kemler, and Karyn A Esser

#### Myology Institute and Department of Physiology and Functional Genomics, College of Medicine, University of Florida

Aging is characterized by a progressive decline in cellular and organismal function and is a primary nonmodifiable risk factor for several chronic diseases. Loss of systemic and tissue‐specific circadian robustness has a negative effect on homeostasis and contributes to an aging phenotype. Reduced molecular clock gene oscillations have been reported in numerous age‐related diseases. However, it is unclear if clock disruptions reported in disease models are due to increasing age or to disease pathophysiology. To determine how non‐pathological aging influences circadian function across the lifespan, we examined molecular clock rhythms in skeletal muscle explants from young (4 to 8 months) and old (22 to 28 months) circadian reporter mice (PER2::LUC). We found that old PER2::LUC tissues had significantly (*p* < 0.05) dampened molecular clock amplitude compared with young animals (67% reduction). To determine the cellular impact of reduced molecular clock amplitude, we completed RNA sequencing on muscle from mice at three ages across the lifespan (3 months, 16 months, and 28 months) at two times of day (CT1 and CT13). We chose CT1 (inactive) and CT13 (active) as these times correspond to the predicted peak and trough of core molecular clock gene expression. After sequencing with an Illumina HiSeq3000, differential gene expression was assessed using DEseq2 with Benjamini‐Hochberg adjusted FDR < 0.05. With these stringent criteria, we found that young muscle had 239 differentially expressed genes between CT1 and CT13, whereas middle‐aged (123) and old (116) skeletal muscle samples had far fewer differentially expressed genes between CT1 and CT13. Furthermore, we compiled a list of 200 skeletal muscle genes that exhibit the strongest circadian oscillation (data from CircaDB). When this list of genes was compared with the differentially expressed genes we found in young, middle‐aged, or old skeletal muscle samples, we found an age‐related decline in molecular clock output. Together, the reduced molecular clock bioluminescence amplitude paired with a lower number of differentially expressed molecular clock components and clock output genes at the two timepoints suggests changes in the molecular clock are occurring at middle age and progress with advancing age. This suggests alterations in the molecular clock could contribute to age‐associated muscle weakness and overall reductions in skeletal muscle function.


**Christian Wright, PhD**


Indiana University, Indianapolis, IN, USA

DOI: 10.002/jbm4.10331


## Osteocyte‐Specific Deletion of the Auxiliary α2δ1 Voltage‐Sensitive Calcium Channel Subunit Impairs Skeletal Strength and Decreases Both Lean and Fat Masses

### Christian S Wright,^1^ Xin Yi,^1^ Artur Schneider,^2^ Molly Pederson,^3^ Mary C Farach‐Carson,^4^ Alexander G Robling,^5^ and William R Thompson^1,5^


#### 
^1^Department of Physical Therapy, School of Health and Rehabilitation Sciences, Indiana University, Indianapolis, IN, USA; ^2^Department of Physiology, College of Osteopathic Medicine, Marian University, Indianapolis, IN, USA; ^3^School of Science, Indiana University‐Purdue University, Indianapolis, IN, USA; ^4^Department of Diagnostic and Biomedical Sciences, School of Dentistry, University of Texas, Health Science Center, Houston, TX, USA; ^5^Department of Anatomy and Cell Biology, School of Medicine, Indiana University, Indianapolis, IN, USA

Osteocytes are the most abundant and mechanosensitive cells in the skeleton. They are essential for sensing and responding to mechanical forces by controlling the activity of other cells. However, the mechanisms by which osteocytes sense force input and transmit signals to other cells remain unclear. Voltage‐sensitive calcium channels (VSCCs) regulate anabolic responses to mechanical loading, and inhibition or deletion of these channels impairs bone accrual, mechanosensation, and skeletal integrity. Activity of VSCCs is regulated by auxiliary subunits, which bind the pore‐forming α1 subunit to influence calcium influx. Through its transmembrane domain and large extracellular region, the α2δ1 auxiliary subunit controls the calcium‐gating kinetics of the α1 channel pore, the interaction with and subsequent response to extracellular ligands, and the forward‐trafficking of the α1 channel pore to the cell membrane. Knockdown of α2δ1 in MLO‐Y4 osteocytes decreases the cell's ability to respond to membrane stretch, and global deletion of α2δ1 in mice results in osteopenia. Therefore, we hypothesized that osteocyte‐specific deletion of α2δ1 would impair skeletal development. Mice (C57BL/6) with LoxP sequences flanking crucial exons of Cacna2d1, the gene encoding α2δ1, were crossed with mice expressing Cre under the control of the Dmp1 promoter (10 Kb). To assess skeletal phenotype, longitudinal whole body and site‐specific DXA and in vivo μCT (10 weeks old) were assessed. Three‐point bending and ex vivo μCT were also conducted after euthanization (20 weeks old). Osteocyte‐specific deletion of α2δ1 in male mice decreased femoral BMC (*p* = 0.0213) by DXA and impaired cancellous bone at the proximal tibia by μCT, showing decreased trabecular thickness (*p* = 0.0097) and a trend for lower BV/TV (*p* = 0.057) at 10 weeks. Cacna2d1f/f, Cre + male mice also displayed reduced ultimate force (*p* = 0.0009) and energy to failure (*p* = 0.0151) with femora three‐point bending, compared with Cacna2d1f/f, Cre‐ controls. In addition to these skeletal outcomes, osteocyte‐specific deletion of α2δ1 decreased total body lean (*p* = 0.0362) and fat (*p* = 0.009) masses by DXA. Collectively, the α2δ1 auxiliary subunit is essential for osteocyte's regulation of trabecular structure and femur strength. Furthermore, these data suggest that the α2δ1 auxiliary VSCC subunit controls release of extracellular signals from osteocytes to regulate body composition.


**Laura Wright, PhD**


Indiana University, Indianapolis, IN, USA

Relevant Session: Muscle‐Bone Interactions in Cancer

DOI: 10.002/jbm4.10332


## The Tamoxifen Metabolite Endoxifen Improved Bone Morphology and Reduced Muscle Function in Ovariectomized Mice

### Laura E Wright,^1^ Jenna N Regan,^1^ Yun She,^1^ Sutha John,^1^ Sremala Murthy,^1^ Khalid S Mohammad,^1^ Alexander G Robling,^2^ and Theresa A Guise^1^


#### 
^1^Department of Medicine, Division of Endocrinology, Indiana University School of Medicine, Indianapolis, IN, USA; ^2^Department of Anatomy and Cell Biology, Indiana University School of Medicine, Indianapolis, IN, USA

Endoxifen, the predominant CYP2D6 metabolite of the selective estrogen receptor modulator (SERM) tamoxifen, is currently being developed as a novel anti‐estrogen therapy for the treatment of estrogen receptor (ER)+ breast cancer. Genetic polymorphism of CYP2D6 is a predictor of response to tamoxifen in patients, implicating endoxifen as one of the most active and relevant tamoxifen metabolites. Breast cancer patients treated with adjuvant endocrine therapies including aromatase inhibitors (AIs) and SERMs often report unmanageable musculoskeletal toxicities that can result in treatment discontinuation. Although ER binding affinity and anti‐tumor effects have been established in preclinical models, the effects of endoxifen on the musculoskeletal system are not fully known. Twenty‐week‐old female C57BL/6 mice underwent sham surgery or ovariectomy (OVX) and were treated daily with vehicle, the AI letrozole, or the SERM endoxifen. Body composition was assessed prospectively by DXA. Bone indices and marrow adipose tissue volume were measured by μCT and muscle contractility of the extensor digitorum longus (EDL) was measured ex vivo. After 8 weeks, trabecular bone volume fraction (BV/TV) decreased by 50% in OVX‐vehicle and OVX‐letrozole mice, whereas BV/TV increased threefold in OVX‐endoxifen mice relative to sham‐vehicle. Biomechanical properties of bone were improved by endoxifen, evidenced by an increase in ultimate force and energy to ultimate force in OVX‐endoxifen femur midshafts relative to OVX‐vehicle and OVX‐letrozole groups. Uterine weight increased significantly in endoxifen‐treated mice relative to all OVX groups, in line with what is typically observed with SERM treatment. Peripheral body fat, bone marrow adipose tissue, and circulating leptin were reduced by endoxifen, suggesting that the drug may elicit positive systemic metabolic effects. At the termination of the study, muscle‐specific force was reduced in OVX‐endoxifen mice relative to sham‐vehicle, OVX‐vehicle, and OVX‐AI mice, despite no change in muscle mass. Although endoxifen shows promise as a potent anti‐estrogen therapy for the treatment of ER+ breast cancer, it may be important to monitor patients for endometrial proliferation and muscle weakness, which could influence drug compliance.


**Chenxi Yan, PhD**


University of Illinois Urbana Champaign, Urbana, Illinois.

Relevant Session: Biomechanical Relationships Between Muscle and Bone

DOI: 10.002/jbm4.10333


## Effect of Fatigue on Impact Stress in Bone

### Chenxi Yan,^1^ Stuart Warden,^2^ and Mariana E Kersh^1^


#### 
^1^University of Illinois Urbana‐Champaign; ^2^Indiana University – Purdue University, Indianapolis

While bone experiences loading via muscles during everyday movement, the fatigue loading of bone is thought to be a primary contributor to stress fractures under strenuous activities. The development of woven bone, in response to fatigue loading, results in an increase in structural properties under bending, but whether woven bone can mitigate impact loading is not known. The purpose of this study was to understand the effect of fatigue loading with different recovery times on mechanical impact properties. Using an established fatigue loading protocol in rats (*n* = 12), the right forelimbs were cyclically loaded in compression using a Haversine waveform of 0.055 N/g body weight at 2 Hz while under anesthesia. Loading was stopped when the peak displacement reached 0.75 ± 0.2 mm. Half of the subjects were allowed 24 hours of rest and the remaining six were allowed 7 days of rest before euthanizing. Contralateral limbs for all subjects served as controls. The ulnae were harvested and micro‐CT scanned (5 micron isotropic) after which they were assembled into custom fixtures designed for impact testing. The impact energy was equal to 17.5% of the potential energy of a rat landing from 0.2 m, and peak impact forces were measured using a load cell. Five of the six ulnae with loading and 1 day of rest experienced catastrophic fracture, whereas only one of the six ulnae with 7 days of rest fractured under impact loading. Micro‐CT analyses revealed noticeable woven bone formation on the periosteal edge of fatigue‐loaded bones with 7 days of rest, and peak measured force was 38.73% lower in the 7‐day rest group (*p* = 0.006). Dynamic finite element analyses simulating the impact testing revealed a 43% decrease in the average tensile stress and a 37% decrease in compressive stress within the 7‐day rest group compared with their contralateral unloaded limb. These data suggest that although materially weaker, the presence of woven bone serves as a shock absorber and may attenuate impact energy via micro‐cracking and highlight the importance of rest after fatigue loading.

DOI: 10.002/jbm4.10334


